# *Lactococcus A* phages predict ACLF while *Enterococcus B* phages predict bacterial infection in decompensated cirrhosis

**DOI:** 10.1016/j.jhepr.2025.101622

**Published:** 2025-10-10

**Authors:** Lore Van Espen, Maximilian Joseph Brol, Lila Close, Robert Schierwagen, Wenyi Gu, Marisa I. Keller, Boglarka Balogh, Anthony Fullam, Lander De Coninck, Tomohiro Nakamura, Michael Kuhn, Peer Bork, Wim Laleman, Jasmohan S. Bajaj, Maria Papp, Bernd Schnabl, Jonel Trebicka, Jelle Matthijnssens, MICROB-PREDICT

**Affiliations:** 1 KU Leuven, Department of Microbiology, Immunology, & Transplantation, Rega Institute, Division of Clinical & Epidemiological Virology, Laboratory of Viral Metagenomics, Belgium; 2 Department of Internal Medicine B, University of Münster, Münster, Germany; 3 Structural and Computational Biology Unit, European Molecular Biology Laboratory, Heidelberg, Germany; 4 Division of Gastroenterology, Department of Internal Medicine, Faculty of Medicine, University of Debrecen, Debrecen, Hungary; 5 Department of Medicine, University of California San Diego, La Jolla, CA, USA; 6 Max Delbrück Centre for Molecular Medicine, Berlin, Germany; 7 Department of Bioinformatics, Biocenter, University of Würzburg, Würzburg, Germany; 8 Department of Gastroenterology & Hepatology, Section of Liver & Biliopancreatic disorders and Liver Transplantation, University Hospitals Leuven, KU Leuven, Leuven, Belgium; 9 Division of Gastroenterology, Hepatology, and Nutrition, Virginia Commonwealth University and Richmond VA Medical Center, Richmond, USA; 10 Department of Clinical Research, Faculty of Health Sciences, University of Southern Denmark, Odense, Denmark; 11 European Foundation for the Study of Chronic Liver Failure, Barcelona, Spain

**Keywords:** acutely decompensated cirrhosis, acute-on-chronic liver failure, fecal virome, fecal microbiome, gut phages, phage host prediction

## Abstract

**Background & Aims:**

As portal hypertension progresses in cirrhosis, bacterial translocation across a compromised gut barrier leads to endotoxemia, systemic inflammation and immune dysfunction. Gut phages play a key role in these processes by influencing bacteria-host interactions. This study explores the role of the human gut virome in acute decompensation of cirrhosis and acute-on-chronic liver failure (ACLF).

**Methods:**

The fecal virome was longitudinally assessed by metagenomic sequencing in two independent cohorts: 93 patients (292 samples) with acute decompensation or ACLF from the PREDICT study, and 94 patients (94 samples) with decompensated cirrhosis undergoing TIPS (transjugular intrahepatic portosystemic shunt) surgery collected in a tertiary care setting. Besides descriptive analysis, phages were grouped according to their predicted bacterial host and lifestyle, and associated with clinical parameters.

**Results:**

Phage alpha-diversity was higher in patients with ACLF and correlated with ACLF severity. In the absence of ACLF, the phageome was dominated by virulent phages, but in ACLF, temperate phages became more prevalent. Genus-level analysis showed that phageomes were highly patient-specific. *Lactococcus A* phages were the only phage-host group predicting ACLF development (odds ratio [OR] = 14; Fisher test *p* = 0.0129). *Enterococcus B* phages (OR = 14.7; *p* = 0.0015; adj. *p* = 0.037) and their bacterial hosts (OR = 2.8; *p* = 0.020) were significantly more prevalent in cases of proven systemic bacterial infection. The presence of both phage families was linked to increased 90-day mortality rates.

**Conclusion:**

ACLF is characterized by increased fecal virome diversity and a shift from virulent toward temperate phages at disease onset. Our study links *Lactococcus A* phages to ACLF development, and *Enterococcus B* phages to bacterial infection, while both are associated with increased 90-day mortality.

**Clinical trial number:**

NCT03056612.

**Impact and implications:**

The human gut virome is a poorly investigated part of the human gut microbiome, especially in the context of decompensated cirrhosis and acute-on-chronic liver failure. This study identified two phage groups (*Lactococcus A* phages and *Enterococcus B* phages) with particular prognostic value. In the future, virome analysis of fecal samples could be useful for patient stratification in clinical practice.

## Highlights

• ACLF is associated with a higher fecal virome alpha-diversity.
• ACLF onset is associated with a shift from virulent to temperate phages.
• *Lactococcus A* phages are associated with disease progression towards ACLF.
• *Enterococcus B* phages are associated with systemic bacterial infections.
• Both *Lactococcus A* and *Enterococcus B* phages are associated with increased 90-day mortality.

## Graphical abstract

**Figure:**
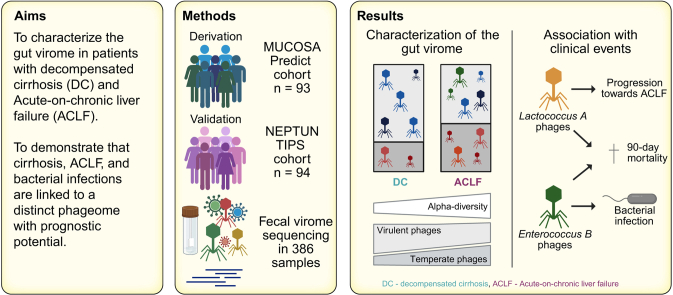

## Introduction

Decompensated cirrhosis and its progression towards acute-on-chronic liver failure (ACLF) are a major cause of death in patients with chronic liver diseases.^1^^,^^2^ The driving mechanisms for decompensation are portal hypertension and systemic inflammation, which are hastened amongst other factors by bacterial infections. The PREDICT study delineated three distinct courses in decompensated cirrhosis that differ significantly in terms of risk for ACLF development and mortality: mortality increases from stable decompensated cirrhosis through unstable decompensated cirrhosis, to pre-ACLF.^3^^,^^4^ Understanding the complex mechanisms underlying the transition from decompensated cirrhosis to ACLF is essential for improving patient outcomes. Bacterial infections and alcohol-associated hepatitis have been identified as the most important triggers of ACLF.^5^ However, in 30-40% of patients who develop ACLF no precipitating event can be detected. In these patients, it is presumed that the trigger for the development of ACLF is the translocation of gut microbes across an impaired gut barrier.^6^

The gut virome refers to the genomes of a diverse community of commensal viruses residing in the gastrointestinal tract, typically comprising a small number of eukaryotic viruses and a larger proportion of bacteriophages that infect the bacterial component of the gut microbiota. Phages can affect the human host through their close interactions with the gut bacteria and are known to be relevant in human health and disease.^7^ For example, phages can alter the composition of the gut bacterial community through bacterial lysis as part of the phage replication cycle.^8^ Temperate phages can mediate horizontal gene transfer between bacteria, potentially shaping the functional properties of the gut bacteria including anti-microbial resistance.^9^ Apart from these indirect effects on human health through the gut bacterial community, phages can also directly engage with the human host via its immune system.^10^^,^^11^ Recent bioinformatic tool developments in the field of viral metagenomics have enabled comprehensive profiling of the gut virome, including better identification of phages^12^^,^^13^ and bacterial host prediction.^14^^,^^15^ Phage host prediction allows for an improved biological interpretation, as changes in phages infecting a distinct group of bacteria can shed light on the specific phage-bacteria dynamics of the gut microbiome.

While extensive research has focused on characterizing alterations of gut bacteria in relation to decompensated cirrhosis and its complications,^16^^,^^17^ little is known about interactions of the virome with progression of decompensated cirrhosis, though the virome has been implicated in other types and stages of chronic liver disease.[18], [19], [20], [21], [22] For example, progression of decompensated cirrhosis is associated with increased abundance of phages infecting *Enterobacteriaceae* and *Lactobacillaceae* bacteria and decreased abundance of crAssphages, a bacteriophage family representing the most abundant viruses in the human gut.^18^ In mice, phages targeting cytolysin-positive *Enterococcus faecalis* reversed the exacerbation of liver disease^23^ and phages targeting ethanol-producing *Klebsiella pneumoniae* prevented the development of liver disease.^24^^,^^25^ Further research into bacterial and viral dysbiosis could help identify other potential targets for phage therapy.^26^ However, it is still unclear which virome perturbations are associated with decompensated cirrhosis and ACLF and whether phages can be used as biomarkers for the onset of ACLF in patients with cirrhosis. This study aims to address this knowledge gap by conducting an extensive analysis of the gut virome in patients with decompensated cirrhosis and ACLF. We hypothesized patients with cirrhosis, ACLF and bacterial infections have a distinct phageome that could be leveraged for prognostication.

## Patients and methods

### Study design, data and sample collection

The samples of the derivation cohort were obtained from 93 patients recruited at the University of Debrecen (Hungary) as part of the PREDICT study (MUCOSA-PREDICT sub-study) (ClinicalTrials.gov number, NCT03056612). The PREDICT study is a European, investigator-initiated, multicenter, prospective, observational study.^54^ The study was approved by the local ethics committee (35281-2/2017/EKU), and all patients signed an informed written consent in accordance with the Helsinki Declaration. Acute decompensation and ACLF were diagnosed as previously described according to the European Association for the Study of the Liver – Chronic Liver Failure (EASL-CLIF) criteria.^1^^,^^27^ Patients were stratified into stable decompensated cirrhosis (n = 53), unstable decompensated cirrhosis (n = 10), pre-ACLF (n = 21) and ACLF (n = 9) based on previously described criteria.^4^ Patients were retrospectively assigned to the pre-ACLF group if they developed ACLF within the follow-up but did not have ACLF at enrollment. Clinical and laboratory data, as well as biological samples, were collected at multiple time points^4^ (Fig. S1). Briefly, patients were stratified according to their Chronic Liver Failure Consortium (CLIF-C) acute decompensation (AD) score (high risk ≥50; low risk <50). The high-risk group had scheduled follow-up after 1, 4, 8 and 12 weeks, while the low-risk group was followed up solely at week 1 and 12. All patients who developed ACLF during the observational period had two additional unscheduled visits (ACLF onset visit and another 7 days later).

The samples for the validation cohort were obtained from 98 patients with decompensated cirrhosis who received a transjugular intrahepatic portosystemic shunt (TIPS) as part of the NEPTUN study (NCT03628807) at the Department of Internal Medicine I, University Clinic Bonn (Germany).[28], [29], [30] The stool samples were collected during inpatient treatment before TIPS placement and stored immediately at -80 °C degrees until analysis. The study was approved by the local ethics committee of the University of Bonn (029/13), and all patients signed an informed written consent in accordance with the Helsinki Declaration. Fecal samples were processed for virome sequencing in the same manner as the MUCOSA-PREDICT samples.^31^ Bioinformatic processing of sequencing reads (including quality control, assembly, viral identification, abundance determination, lifestyle and host prediction) was performed in the same fashion as the MUCOSA-PREDICT samples. Good-quality fecal virome profiles were generated for 94 samples.

### Fecal virome profiling

Viral-like particles were purified from fecal samples and prepared for virome sequencing using the NetoVIR protocol.^31^ Sequencing reads were quality-controlled and assembled, followed by clustering of the resulting scaffolds >1 kb at 95% identity over 85% coverage to remove cross-sample redundancy. Viral scaffolds were identified using a combination of VirSorter2^12^ and similarity to known viruses using DIAMOND^32^ and/or BLASTn,^33^ and their genome completeness was assessed using CheckV.^34^ A horizontal coverage cut-off of 70% was applied to determine the presence of a scaffold in a sample. The host genus of a phage was predicted using a combination of methods (*e.g*. CRISPR similarity, tRNA similarity, prophage similarity, kmer sharing and protein content) relying on a database of bacterial metagenomic-assembled genomes (MAGs) from the same fecal samples and from a publicly available database.^35^

Methods on the profiling of the fecal bacteriome and more details on the virome profiling can be found in the supplementary methods and Fig. S2.

### Ecological analyses, statistical analyses and visualization

Data analysis and visualization were performed in R (v4.3.1).^36^ Statistical significance was defined as *p* <0.1 and correction for multiple hypothesis testing was applied where appropriate using the Benjamini-Hochberg method.^37^

Alpha-diversity (Simpson diversity) was compared between two groups of samples using a two-tailed Wilcoxon rank-sum test. Relative abundance of phages grouped by their lifestyle were compared between and within samples using two-tailed (paired) Wilcoxon rank-sum tests. Associations of alpha-diversity and relative abundances of temperate and virulent phages with disease scores were analyzed using Pearson’s correlation coefficient.

The inter-individual phageome variation was calculated using Bray-Curtis dissimilarity on the phage genus level and visualized using principal coordinate analysis. The contribution of the clinical covariates on the inter-individual phageome variation was determined using univariate distance-based redundancy analysis (dbRDA) using the *capscale* function.^38^ Samples with missing data were excluded for the respective univariate analysis and only covariates with less than 10% missing data were included. Clinical covariates were transformed (log_10_, inverse or square root) to better approach a normal distribution. The cumulative non-redundant contribution of clinical covariates on the phageome variation was determined using stepwise multivariate dbRDA (forward-selection model) using the *ordiR2step* function.^38^ No correction for multiple testing was performed in the multivariate dbRDA since only variables with an adjusted *p* value <0.1 in the univariate dbRDA were included in the multivariate dbRDA. Samples with missing data for any of the significant univariate covariates were excluded.

As phages most likely affect the human host through their bacterial hosts, differential abundance analysis of phages was done by grouping them according to their predicted host genus for a more meaningful biological interpretation. Wilcoxon rank-sum tests were used to compare differences in relative abundance of phage-host groups present in at least five samples of interest. Differences in prevalence were assessed using Fisher’s exact tests. Relative abundances of these phage-host groups were log10-transformed with the addition of a pseudocount (0.000001) for visualization purposes. A log-rank test was used to assess differences in short-term (90-day) survival between patients with and without phage-host groups present at any timepoint in at least five patients.

## Results

### The gut virome of patients with decompensated cirrhosis is dominated by microviruses and caudoviruses

**Fig. 1:**
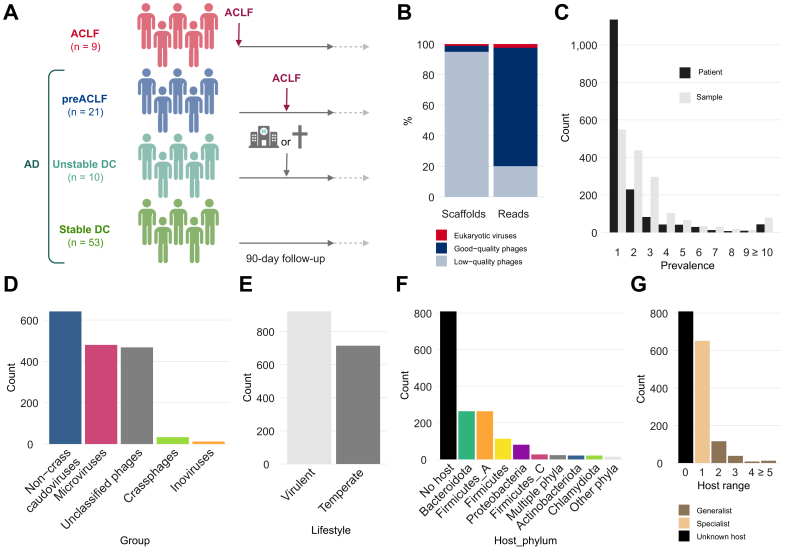
Overview of the clinical cohort and virome dataset. (A) Overview of the clinical cohort. (B) Overview of virome composition at scaffold and read level in all 292 samples. (C) Distribution of good-quality phages (n = 1,635) according to their prevalence (number of patients and samples, respectively). (D-F) Classification of good-quality phages (n = 1,635) according to their predicted taxonomic classification (D), lifestyle (E), bacterial host (F) and host range on the genus level (G). ACLF, acute-on-chronic liver failure; AD, acute decompensation; DC, decompensated cirrhosis.

The MUCOSA-PREDICT cohort consists of 93 patients, 9 of whom had ACLF at enrollment and 84 patients with AD. After a 90-day follow-up period, the patients with AD were retrospectively stratified into three groups: the pre-ACLF group (patients who developed ACLF; n = 21), the unstable decompensated cirrhosis group (patients requiring hospital readmission and/or who died without developing ACLF; n = 10) and stable decompensated cirrhosis (patients who did not have any events during the 90 days; n = 53) (Fig. 1A). Good-quality virome profiles were generated for 292 samples, with an average of three samples per patient. Average disease scores increased from the stable to the unstable decompensated cirrhosis group and further to the pre-ACLF group (Table 1).

**Table 1:** Overview of patient characteristics.

	ACLF	Pre-ACLF	UDC	SDC	*p* value
Patients, n	9	21	10	53	
Samples, n	20	65	37	170	
Samples/Patient, mean ± SD	2.2 ± 0.4	3.1 ± 1.3	3.7 ± 1.2	3.2 ± 0.9	0.004
Sex (Male), n (%)	7 (77.8%)	14 (66.7%)	5 (50.0%)	31 (58.5%)	0.6
Age, mean ± SD	57.4 ± 12.7	63.4 ± 7.7	60.7 ± 13.9	57.6 ± 10.4	0.2
Etiology, n (%)					
Alcohol	9 (100%)	18 (86%)	8 (80%)	47 (89%)	0.6
Hepatitis B virus	0 (0%)	1 (4.8%)	1 (10%)	0 (0%)	0.2
Hepatitis C virus	1 (11%)	1 (4.8%)	0 (0%)	3 (5.7%)	0.7
MASLD	4 (44%)	3 (14%)	0 (0%)	5 (9.4%)	0.03
Cryptogenic	0 (0%)	1 (4.8%)	1 (10%)	1 (1.9%)	0.4
Other	1 (11%)	1 (4.8%)	0 (0%)	3 (5.7%)	0.7

**Baseline**					
Disease scores, mean ± SD [NAs]					
MELD score	19.3 ± 5.9	17.9 ± 6.0	13.9 ± 5.4	13.7 ± 5.1 [1]	0.006
MELD-Na score	22.2 ± 5.7	22.7 ± 7.0	17.7 ± 6.7	16.7 ± 5.7 [1]	0.002
Child-Pugh score	7.8 ± 1.9 [1]	9.4 ± 1.7	9.1 ± 1.7	8.5 ± 1.9 [3]	0.076
CLIF-C ACLF score	43.7 ± 7.3	NA	NA	NA	NA
CLIF-C AD score	NA	57.9 ± 7.8	54.2 ± 7.3	49.1 ± 7.4 [1]	<0.001
Medication, n (%)					
Albumin	1 (11%)	3 (14%)	4 (40%)	5 (9.4%)	0.11
Antibiotics (all)	6 (67%)	10 (48%)	6 (60%)	22 (42%)	0.5
Rifaximin	1 (11%)	1 (4.8%)	0 (0%)	4 (7.5%)	0.8
Lactulose	4 (44%)	2 (9.5%)	1 (10%)	7 (13%)	0.11
Proton pump inhibitors	5 (56%)	7 (33%)	5 (50%)	14 (26%)	0.2

**1 week follow-up** (4-10 days)					
Disease scores, mean ± SD [NAs]					
MELD score	14.1 ± 9.1	17.1 ± 8.5	12.9 ± 5.4	12.4 ± 4.5	0.3
MELD-Na score	17.3 ± 7.9	21.3 ± 7.8	18.2 ± 5.6	15.7 ± 5.7	0.043
Child-Pugh score	7.9 ± 2.0 [2]	8.9 ± 1.8 [7]	8.8 ± 2.0 [1]	7.9 ± 1.6 [11]	0.186
CLIF-C ACLF score	44.5 ± 12.0 [8]	42.8 ± 6.2 [17]	NA	NA	NA
CLIF-C AD score	50.7 ± 3.5 [3]	54.7 ± 7.2 [8]	53.3 ± 7.9	48.5 ± 8.2 [3]	0.033
Medication, n (%) [NAs]					
Albumin	4 (40%)	7 (39%) [3]	3 (30%)	5 (9.4%) [1]	0.11
Antibiotics (all)	9 (90%)	10 (48%) [3]	6 (60%)	22 (42%) [1]	0.5
Rifaximin	1 (11%)	1 (4.8%) [3]	0 (0%)	4 (7.5%) [1]	0.8
Lactulose	4 (44%)	2 (9.5%) [3]	1 (10%)	7 (13%) [1]	0.11
Proton pump inhibitors	7 (70%)	7 (39%) [3]	6 (60%)	19 (37%) [1]	0.12

**All samples from all patients**					
Disease scores, mean ± SD [NAs]					
MELD score	16.6 ± 7.8 [1]	19.3 ± 7.9 [1]	13.1 ± 4.9	12.2 ± 4.4	<0.001
MELD-Na score	20.0 ± 7.0 [1]	23.8 ± 7.4 [1]	17.6 ± 5.8	15.3 ± 5.3	<0.001
Child-Pugh score	7.8 ± 1.8 [4]	9.2 ± 2.0 [5]	8.9 ± 1.8	7.9 ± 1.8 [38]	<0.001
CLIF-C ACLF score	43.5 ± 8.0 [9]	47.8 ± 9.9 [49]	NA	NA	0.5
CLIF-C AD score	51.9 ± 4.6 [12]	57.0 ± 8.4 [17]	52.1 ± 7.3	47.9 ± 7.6	<0.001
Medication, n (%)					
Albumin	5 (25%)	22 (34%)	13 (35%)	9 (5.3%)	<0.001
Antibiotics (all)	15 (75%)	34 (52%)	28 (76%)	70 (41%)	<0.001
Rifaximin	2 (10%)	12 (18%)	3 (8.1%)	12 (7.1%)	0.076
Lactulose	8 (40%)	18 (28%)	6 (16%)	28 (16%)	0.035
Proton pump inhibitors	12 (60%)	21 (32%)	15 (41%)	49 (29%)	0.006

Numbers between squared brackets indicate the number of individuals for whom the variable was NA. Continuous variables are represented as mean ± standard error and compared between groups using Kruskal-Wallis tests. Categorical variables are represented as counts with percentages between brackets and compared between both groups using Fisher’s exact test. ACLF, acute-on-chronic liver failure; AD, acute decompensation; CLIF-C, Chronic Liver Failure Consortium; MASLD, metabolic dysfunction-associated steatotic liver disease; MELD, model for end-stage liver disease; NA, not available; SDC, stable decompensated cirrhosis; UDC, unstable decompensated cirrhosis.

The fecal viromes were dominated by phages, as only 1% of viral scaffolds belonged to eukaryotic viruses, representing 2.5% of the viral reads (Fig. 1B). Further analysis of the virome was focused on the good-quality phages (*i.e.* estimated genome completeness >50%; see supplementary methods) to reduce noise from highly fragmented genomes. These good-quality phages (n = 1,635; 4% of phage scaffolds) represent the majority of phage reads (79%) (Fig. 1B). Most of the phage scaffolds were found in a single patient (69% phage scaffolds) or even a single sample (34% phage scaffolds) (Fig. 1C), while only three phage scaffolds were present in more than half of the patients (“core” phages).^39^

Most of the good-quality phage scaffolds were classified as microviruses (icosahedral single-stranded DNA viruses; 29% phage scaffolds) and caudoviruses (tailed double-stranded DNA viruses; 41% phage scaffolds), including 33 crAssphages (Fig. 1D). The majority of phages (56%) were predicted to be virulent and were therefore unable to integrate their genome into that of their host in contrast to temperate phages (Fig. 1E). Almost a third of the phage scaffolds remained unclassified (29% phage scaffolds), potentially including numerous previously undescribed phages.

Bacteria belonging to the *Bacteroidota* and *Firmicutes C* phyla were the most common predicted hosts of the phages (n = 263 for both phyla; Fig. 1F), which was expected given the dominance of these phyla in the human gut.^40^ Of the 51% of the phages for which a host could be predicted, 174 were linked to multiple bacterial genera (generalist phages; 11% phage scaffolds) compared to 652 specialist phages predicted to infect a single bacterial genus (specialist phages; 40% phage scaffolds) (Fig. 1G). The potential broad host range of human gut phages has been described elsewhere.^41^^,^^42^

### Inter-sample phageome variation is mainly driven by interpersonal differences

**Fig. 2:**
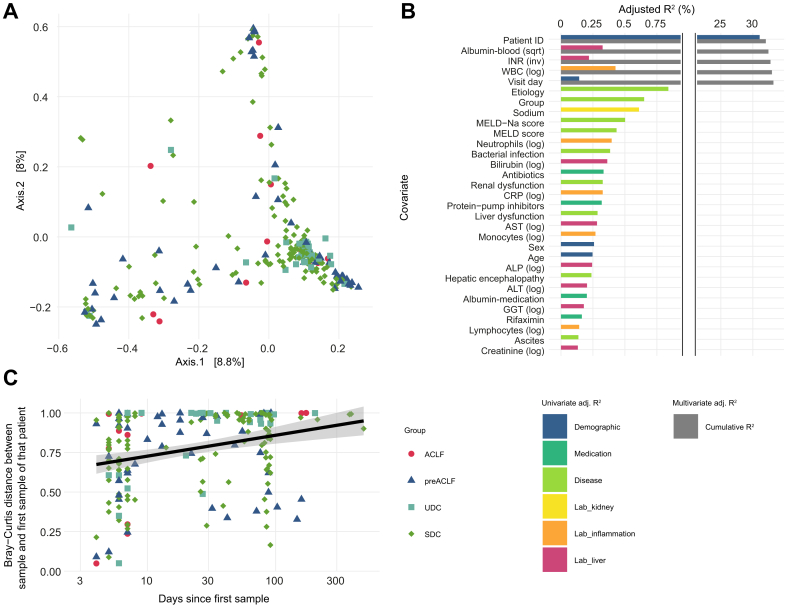
Beta-diversity of the phage community in patients with decompensated cirrhosis and ACLF. (A) PCoA plot of the gut phage community of all samples from all patients colored by disease group (n = 292) (Bray-Curtis distance at the genus level). (B) Individual effect sizes (colored top bars) of covariates on the genus-level phage ordination (univariate dbRDA; n = 31 with FDR <0.1) and cumulative effect sizes (grey bottom bar) of non-redundant covariates (multivariate dbRDA; *p* <0.1) selected by stepwise feature selection based on 268 samples. Covariates are ordered based on cumulative R^2^ for covariates in the multivariate model and decreasing univariate R^2^ for the remaining covariates. (C) Association between days since the first available visit and distance to the first available visit (n = 199, excluding the first available visit (n = 93) of each patient). ACLF, acute-on-chronic liver failure; dbRDA, distance-based redundancy analysis; FDR, false discovery rate; PCoA, principal coordinate analysis; SDC, stable decompensated cirrhosis; UDC, unstable decompensated cirrhosis.

The fecal phage community was profiled at the genus level to explore the inter-sample phageome variation (Bray-Curtis dissimilarity). The limited explained variance on the first two axes of the principal coordinate analysis plot highlight the high inter-sample diversity in this cohort (Fig. 2A). To determine which clinical covariates explained this high inter-sample diversity in phage community, the individual effect size of each covariate (n = 40) was determined using univariate distance-based redundancy analysis (Table S1). The patient identifier was unequivocally the covariate with the most considerable effect on the phage community (univariate R^2^ = 31%). In addition, 30 other covariates such as blood values, disease scores, complications and medication had a limited effect on phageome composition (univariate R^2^ <1%; false discovery rate <0.1; colored bars in Fig. 2B).

All covariates significantly associated with the phage ordination (n = 31/40; false discovery rate <0.1) were subsequently fed into a stepwise forward-selection model to determine the cumulative effect sizes of non-redundant covariates on the phage community composition. For this multivariate analysis, if any of the included covariates were unavailable for a given sample then that sample was removed (n = 24). The removal of these samples resulted in a slightly higher effect of the patient identifier on the phage community of the remaining 268 samples (R^2^ = 32%). Only a few other covariates had a limited additional non-redundant effect on phage community composition. Serum albumin levels (R^2^ = 0.4%), international normalized ratio (R^2^ = 0.3%), and white blood cell count (R^2^ = 0.2%) significantly explained an additional fraction of the phage community variation. Visit day, representing the number of days since the first visit, was the last covariate (R^2^ = 0.2%) that significantly contributed to the cumulative effect of all covariates on the phage community (cumulative R^2^ = 33.2%; grey bars in Fig. 2B).

The impact of time on the phage composition is further highlighted by the correlation between days since the first visit and the distance between the samples and the first available visit of each patient (r_p_ = 0.19; *p* = 0.005; Fig. 2C). This means that the longer the time since the first visit, the more the phage community has diverged from the initial one. Nonetheless, the high inter-sample variation of the phage community remains largely unexplained, as included covariates contributed to only 33.2% of the overall phage community variation.

### Higher phage Simpson diversity associated with ACLF and its mortality

**Fig. 3:**
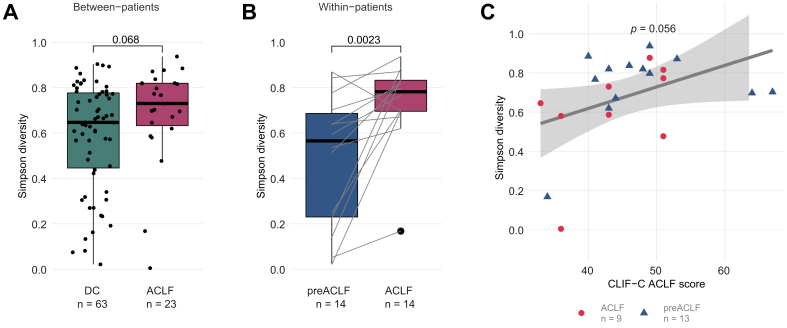
Phage Simpson diversity associated with ACLF progression and its mortality. (A) Inter-individual comparison of phage alpha-diversity between the first sample of patients with stable and unstable decompensated cirrhosis and the first ACLF sample of (pre)-ACLF patients (Wilcoxon rank-sum test). (B) Intra-individual comparison of phage alpha-diversity between the first sample and the first ACLF sample in pre-ACLF patients (Wilcoxon signed-rank test). Lines connect samples from the same patient. (C) Relationship between phage alpha-diversity and CLIF-C ACLF score across the sample of each (pre)ACLF patient at ACLF development (Pearson’s correlation coefficient = 0.41). All samples were derived from the visit of ACLF onset. ACLF, acute-on-chronic liver failure; CLIF-C, Chronic Liver Failure Consortium; DC, decompensated cirrhosis.

We next assessed the changes in phage alpha-diversity (Simpson diversity) in relation to the presence of ACLF. Firstly, alpha-diversity of the phageome tends to be higher in patients with ACLF compared to patients without ACLF (Wilcoxon rank-sum test; *p* = 0.068; Fig. 3A). Here, the first ACLF visits from patients in the ACLF (n = 9) and pre-ACLF groups (n = 14) were compared to the first visits of patients from the stable (n = 10) and unstable (n = 53) decompensated cirrhosis groups. When considering all available samples from all patients regardless of disease group, higher phage diversity was observed in ACLF samples, though not reaching statistical significance (Wilcoxon rank-sum test; *p* = 0.096; Fig. S3). Furthermore, phage alpha-diversity is increased when patients from the pre-ACLF group progress from decompensated cirrhosis to ACLF (Wilcoxon singed-rank test; *p* = 0.023; n = 14; Fig. 3B).

Moreover, alpha-diversity of the gut phageome was positively correlated with the CLIF-C ACLF score, for a surrogate of ACLF-associated mortality, in the sample at ACLF development, without reaching statistical significance (r_p_ = 0.41; *p* = 0.056; n = 22; Fig. 3C) as well as across all ACLF samples from all patients (r_p_ = 0.47; *p* = 0.013; n = 27; Fig. S4A). No association of phage diversity with the model for end-stage liver disease (MELD) score was observed (*p* = 0.61; n = 290; Fig. S4B). Finally, neither the use of proton pump inhibitors nor of antibiotics was associated with statistically significant differences in alpha-diversity in gut viromes of patients with ACLF (*p* = 0.11 and *p* = 0.7, respectively, Fig. S5). These findings indicate that phage alpha-diversity might be elevated in the presence of ACLF and positively linked to the development and severity of ACLF.

### Disease progression associated with increased relative abundance of temperate phages

**Fig. 4:**
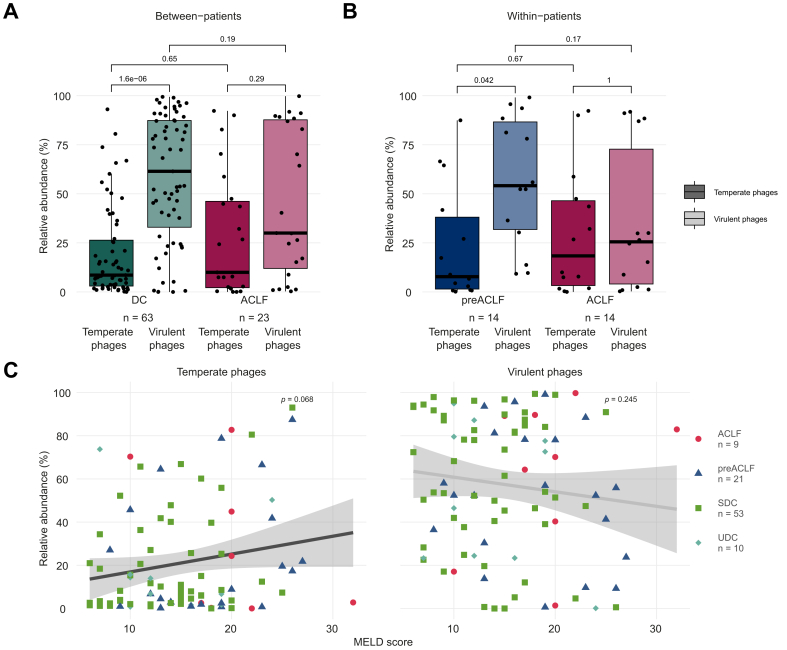
Disease progression is marked by a shift in phageome composition in terms of phage lifestyle. (A) Phageome composition in terms of phage lifestyle in the first sample of patients with stable and unstable decompensated cirrhosis and the first ACLF samples of (pre)-ACLF patients (Wilcoxon rank-sum tests). (B) Phageome composition in terms of phage lifestyle between the first sample and the first ACLF sample of pre-ACLF patients (Wilcoxon signed-rank tests). (C) Relationship between MELD score and relative abundance of temperate (left panel) and virulent (right panel) phages in the first visit of each patient (Pearson’s correlation coefficient = 0.19 and -0.12 (n.s.)). ACLF, acute-on-chronic liver failure; DC, decompensated cirrhosis; MELD, model for end-stage liver disease.

In addition to the observed changes in viral diversity, we assessed whether progression towards ACLF was also associated with the composition of the gut phageome in terms of phage lifestyle. Temperate phages can integrate their genomes into their bacterial host genome and replicate with their host (lysogenic lifecycle), in addition to following a lytic lifecycle. Healthy adult viromes are dominated by virulent phages, which can only replicate using the lytic lifecycle.^43^ Gut phageomes of the first sample of patients with decompensated cirrhosis were likewise dominated by virulent phages, with limited presence of temperate phages (Fig. 4A). In ACLF viromes (first ACLF visit of (pre)ACLF patients), on the other hand, the virulent phages still dominated, but the difference was not statistically significant. The dominance of virulent phages was also observed in pre-ACLF patients before the onset of ACLF (Fig. 4B). At ACLF onset, however, this predominance of virulent phages was again diminished, at the expense of temperate phages. A similar observation was made in all available samples when comparing samples with and without ACLF (Fig. S6).

Although the elevated levels of temperate phages in ACLF viromes are not significantly different from those in non-ACLF viromes (Figs 4A,B, and S6), their relative abundance, although also not significant, correlated positively (r_p_ = 0.19; n = 93; Fig. 4C left) with the MELD score, as a marker for disease severity, in the first sample of each patient. The relative abundance of temperate and virulent phages furthermore associated positively and negatively, respectively, with MELD score when considering all available samples (r_p_ = 0.17 and r_p_ = -0.17; n = 290; Fig. S7A), but not CLIF-C ACLF score (*p* = 0.82 and *p* = 0.56, respectively; n = 27; Fig. S7B). These results suggest that disease progression is associated with a shift from a virulent-dominated gut virome towards a phageome with a lower relative abundance of virulent phages.

### *Lactococcus A* phages predict ACLF development and are associated with higher short-term mortality

**Fig. 5:**
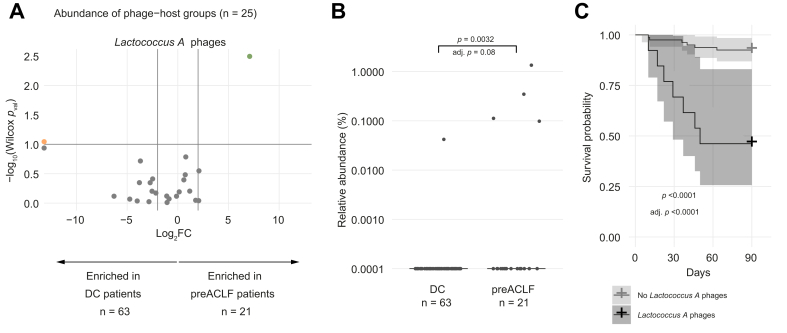
*Lactococcus A* phages associated with pre-ACLF and higher short-term mortality. (A) Volcano plot to visualize changes in abundance of phage-host groups (n = 25) between pre-ACLF and stable and unstable decompensated cirrhosis present in the first sample of at least 5/84 patients (Wilcoxon rank-sum test; orange dots = *p* <0.1; green dots = FDR <0.1). (B) Relative abundance of *Lactococcus A* phages in the first visit of decompensated cirrhosis *vs*. pre-ACLF (Wilcoxon rank-sum test; FDR-adjusted for 25 phage-host groups present in the first sample of at least 5/84 patients). (C) 90-day survival analysis of patients with (n = 14) and without (n = 79) *Lactococcus A* phages (first sample with these phages within 90 days from first sample *vs.* first sample) (Log-rank test; FDR-adjusted for 40 phage-host groups present in at least 5/93 patients at any timepoint). ACLF, acute-on-chronic liver failure; DC, decompensated cirrhosis; FDR, false discovery rate.

Phages likely exert their effects on the human host indirectly through their bacterial hosts; therefore, specific phages have been associated with clinical features by grouping them according to their predicted host genus for a more meaningful biological interpretation. We assessed whether any specific phage-host groups (n = 25) were enriched in patients with pre-ACLF (n = 21) compared to those with stable and unstable decompensated cirrhosis (n = 63) at their first visit (Fig. 5A, Table S2). *Lactococcus A* phages were the only group of phages significantly more abundant in the first samples of patients with pre-ACLF (Fig. 5B). However, their increased prevalence in the first visit of patients with pre-ACLF was no longer significant after correction for multiple hypothesis testing (Fisher test: *p* = 0.01287; adj. *p* = 0.32181; odds ratio [OR] = 14; 1/63 *vs.* 4/21, Table S2). *Lactococcus A* phages were also more abundant when considering all samples of patients with pre-ACLF before ACLF onset (n = 39) compared to all samples from patients with decompensated cirrhosis (n = 207) (*p* <0.0001; Fig. S7). In contrast, the abundance and prevalence of *Lactococcus A* bacteria was not increased in pre-ACLF compared to stable and unstable decompensated cirrhosis (Fig. S9A; Fisher test: *p* = 1; OR = 1; 9/63 *vs.* 3/21).

The group of *Lactococcus A* phages associated with future ACLF development consists of two partial caudoviral genomes predicted to be temperate (Table S3). A potential explanation for the increased abundance of these temperate *Lactococcus A* phages without a simultaneous increase in *Lactococcus A* bacteria is an induction of these phages from their integrated prophage state. One of the *Lactococcus A* phage representatives showed high similarity (99.8%) to a *Lactococcus A* bacterial MAG, assembled from a patient prior to ACLF onset. In that same sample, three fragments of that *Lactococcus A* phage (1.8 kb, 1.9 kb and 6.4 kb) were found to be nearly identical (1 mismatch) to the *Lactococcus A* bacterial MAG, potentially indicating a recent induction, although it cannot be excluded that parts of the prophage were sequenced instead of the extracellular phage. Inflammation levels have previously been associated with the induction of prophages into their extracellular form.^44^ The inflammation preceding and associated with ACLF onset might therefore contribute to the induction of these *Lactococcus A* phages.

Furthermore, the association between short-term (90-day) mortality and the presence of phage-host groups (n = 40) identified two mortality-associated phage-host groups: *Lactococcus A* phages and *Enterococcus B* phages (Table S4). Out of the 13 patients harboring *Lactococcus A* phages in any of the investigated longitudinal samples during the 90-day follow-up period, seven patients died following ACLF development (Fig. 5C). The presence of *Lactococcus A* bacteria was also associated with worse short-term survival (Fig. S9B). Combined with the previous section, these results suggest inflammation-induced activation of prophages might be an important factor implicated in the progression of decompensated cirrhosis. More specifically, *Lactococcus A* phages are an important group of phages in patients prior to ACLF development and a strong marker for poor short-term survival.

### *Enterococcus B* phages are associated with proven bacterial infection and higher short-term mortality

**Fig. 6:**
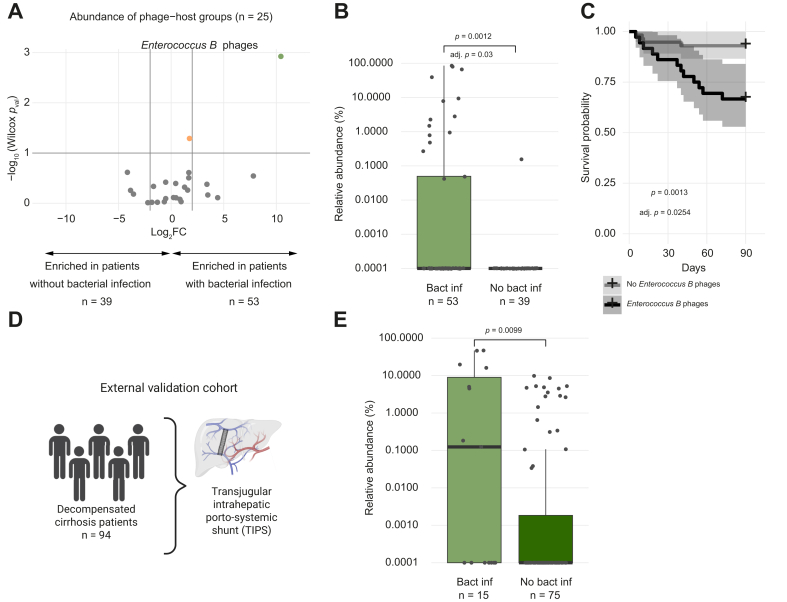
*Enterococcus B* phages are associated with bacterial infection and worse short-term survival. (A) Volcano plot to visualize changes in abundance of phage-host groups (n = 25) between patients with and without bacterial infection present in at least 5/92 samples (first sample with bacterial infection *vs.* first sample of patients without bacterial infection) (Wilcoxon rank-sum test; orange dots = *p* <0.1; green dots = FDR <0.1). (B) Relative abundance of *Enterococcus B* phages in the first sample with bacterial infection *vs*. first sample of patients without bacterial infection (Wilcoxon rank-sum test; FDR-adjusted for 25 phage-host groups present in at least 5/92 samples). (C) 90-day survival analysis of patients with (n = 38) and without (n = 55) *Enterococcus B* phages (first sample with *Enterococcus B* phages within 90 days from first sample *vs.* first sample) (Log-rank test; FDR-adjusted for 40 phage-host groups present in at least 5/93 patients at any time point). (D) External validation cohort of patients with decompensated cirrhosis undergoing a transjugular intrahepatic portosystemic shunt. (E) Relative abundance of *Enterococcus B* phages in patients with and without proven bacterial infection in the validation cohort (Wilcoxon rank-sum test). FDR, false discovery rate.

Bacterial translocation to the portal system and systemic bacterial infections are important precipitating events in the progression of decompensated cirrhosis.^5^ Therefore, we assessed if any phage-host group (n = 25) was enriched in patients with a bacterial infection, given the close interaction between phages and bacteria (Table S5, Fig. 6A). *Enterococcus B* phages were the only phage-host group enriched in the first available sample of patients with proven bacterial infection compared to the first sample of patients without active or future bacterial infection (Fig. 6B). The increased abundance of these phages coincided with elevated levels of *Enterococcus B* bacteria in patients experiencing bacterial infection (Fig. S10A), in line with previous observations.^45^ The relative abundance of *Enterococcus B* is higher in all patients with bacterial infections, reaching significance only in patients with pre-ACLF and stable decompensated cirrhosis (Fig. S10B). Both *Enterococcus B* phages and the bacteria were also more prevalent in these patients (Fisher test phages: *p* = 0.00149; adj. *p* = 0.03734; OR = 14.7; 15/53 *vs.* 1/40; Fisher test bacteria: *p* = 0.02009; OR = 2.8; 34/53 *vs.* 15/39). Most of the *Enterococcus B* phages are predicted to be temperate and therefore theoretically able to integrate into the genomes of their bacterial hosts, mainly *E. faecium*, *E. hirae* and *E. lactis* (Table S6).

Apart from *Lactococcus A* phages, *Enterococcus B* phages were the only other group of phages associated with a worse 90-day survival rate (Fig. 6C). Moreover, the presence of *Enterococcus B* bacteria was associated with worse 90-day survival in these patients (Fig. S10C). In conclusion, *Enterococcus B* phages are strongly associated with bacterial infection and patients harboring these phages have worse short-term survival. No phage-host groups were associated with any other precipitating events (gastrointestinal bleeding, hepatic encephalopathy or severe alcohol-related hepatitis) (Tables S7–9, Fig. S11).

### Validation of the association of *Enterococcus B* phages with proven bacterial infection

The association of *Enterococcus B* phages with bacterial infection was confirmed in a validation cohort of 94 patients with decompensated cirrhosis (Table 2, Fig. 6D). Short-term survival rates were too low to validate this finding. Both the relative abundance and the prevalence of *Enterococcus B* phages were higher in patients with bacterial infection (Fig. 6E; Fisher test: *p* = 0.05996; OR = 3.3; 8/15 *vs*. 19/75; data on bacterial infections was unavailable for four patients). In multivariable analysis, including CLIF-C AD score, ongoing antibiotic treatment and indication for TIPS, only *Enterococcus B* phages were significantly associated with the presence of proven bacterial infection (*p* = 0.041; Table S10). Most of the *Enterococcus B* phages in the validation cohort were also predicted to be temperate phages infecting *E. faecium*, *E. lactis* and *E. hirae* (Table S11), similar to the *Enterococcus B* phages of the MUCOSA-PREDICT cohort (Table S6). These findings highlight the potential importance of these phages, and their bacteria, in the disease course of patients with decompensated cirrhosis.

**Table 2:** Overview of patient demographics and disease scores in validation cohort.

	TIPS validation cohort
Patients, n	94
Samples, n	94
Sex (Male), n (%)	49 (52.1%)
Age, mean ± SD	57.6 ± 13.2
Etiology, n (%)	
Alcohol	53 (56.4%)
Hepatitis C virus	8 (8.5%)
Alcohol + hepatitis C virus	3 (3.2%)
Other	30 (31.9%)
TIPS indication, n (%)	
Variceal bleeding	24 (25.5%)
Ascites	49 (52.1%)
Variceal bleeding + ascites	8 (8.5%)
Portal vein thrombosis	5 (5.3%)
Other	8 (8.5%)
Disease scores, mean ± SD [NAs]	
MELD score	12.5 ± 5.9 [4]
MELD-Na score	14.6 ± 6.5 [4]
Child-Pugh score	7.5 ± 1.8 [5]
CLIF-C AD score	48.4 ± 8.6 [2]

Numbers between squared brackets indicate the number of individuals for whom the variable was NA. Continuous variables are represented as mean ± standard error and categorical variables are represented as counts with percentages between brackets. AD, acute decompensation; CLIF-C, Chronic Liver Failure Consortium; MELD, model for end-stage liver disease; NA, not available; TIPS, transjugular intrahepatic portosystemic shunt.

## Discussion

In the present study, we analyzed the impact of the gut virome on disease progression and prognosis in a large cohort of patients with cirrhosis. We found that *Lactococcus A* phages are associated with ACLF development and that *Enterococcus B* phages are associated with bacterial infections and mortality.

Bacterial infections are the major precipitating event for ACLF, while bacterial translocation from the gut into the liver is thought to be another important trigger. The gut virome plays a pivotal role in maintaining homeostasis in the gut microbial ecosystem, thereby influencing the overall health of an individual.^46^ However, its relevance in the context of decompensated cirrhosis and ACLF has not been studied in detail. In this study, we comprehensively investigated gut virome composition in a well-characterized longitudinal cohort of patients with decompensated cirrhosis and ACLF through optimized sequencing and bioinformatic methods. We demonstrated an association of *Enterococcus B* phages with proven bacterial infection and short-term mortality. In addition, we validated the association of *Enterococcus B* phages with bacterial infection in a similarly large external validation cohort.

The presence of phages targeting *Enterococcus B* bacteria, especially *E. faecium*, *E. hirae* and *E. lactis*, was shown to be associated with mortality and bacterial infection, an important precipitating event in decompensated cirrhosis.^5^ *Enterococcus* phages have previously emerged as key features in the progression and development of alcohol-related hepatitis,^47^^,^^48^ cirrhosis^18^ and ACLF,^17^ especially *E. faecalis*. Moreover, phages targeting cytolysin-positive *E. faecalis* have demonstrated a beneficial effect in alcohol-related hepatitis.^23^ Interestingly, previous studies demonstrated that cytolysin itself, a toxin from *E. faecalis*, was not associated with disease progression.^49^

On the other hand, *Enterococcus* bacteria were found to be associated with disease progression in multiple studies, especially in patients with hepatitis B virus-related ACLF.^50^^,^^51^ *Enterococcus B* phages may serve as a predictive biomarker of systemic bacterial infections. Their association with increased short-term mortality and bacterial infection underscore the need for adequate and timely antibiotic treatment, which is currently the only effective treatment option in patients with ACLF aside from liver transplantation.^52^ Though liver transplantation represents an effective salvage therapy, several risk factors are associated with higher mortality, such as recent infection from multidrug resistant organisms.^53^ In this context, phage characterization might represent an important tool enabling clinicians to select suitable antibiotics in the future. While several precipitants of ACLF have been identified,^5^ there are so far no biomarkers to predict ACLF development. Progression of decompensated cirrhosis is difficult to predict, making it essential to elucidate novel factors contributing to the development of complications. Moreover, in daily clinical practice, ACLF prediction is an unmet need, which is highly relevant given its high mortality rate. Currently available tools have several limitations: Child-Pugh and MELD scores both accurately predict mortality, but not ACLF onset. Moreover, all scores lack in explaining a pathogenic relationship. Our study is the first to identify *Lactococcus A* phages as being associated with both the onset of ACLF and increased 90-day mortality, and they may serve as predictive and prognostic biomarkers. Although this result still requires validation, due to the lack of an appropriate cohort, this finding is important. In 30-40% of patients with ACLF, no precipitating event was identified in the CANONIC and PREDICT studies. These cases might be explained by microbial translocation. In this cohort, *Lactococcus A* phages may play an important role. It is well described, that fecal virulent factors may be modulated.^23^ *Lactococcus A* phages may change the homeostasis of the gut virome, possibly resulting in local and subsequent systemic inflammation. Ultimately, systemic inflammation may induce organ failures, which leads to development of clinically overt ACLF. Identification of patients with pre-ACLF is therefore of major importance for preventing ACLF.

On the level of individual phages and their bacterial hosts, several phage-host groups associated with clinical parameters. *Lactococcus* phages have previously been reported to be associated with alcohol use and severity in metabolic dysfunction-associated steatotic liver disease^26^ and alcohol use disorder.^48^ However, Jiang *et al.* reported a decrease in *Lactococcus* phages in alcohol-related hepatitis.^47^

Collectively, the existing literature and our results indicate that interactions between *Lactococcus* and *Enterococcus* bacteria and their phages are relevant in the development and progression of chronic liver disease. This is further highlighted by the association of *Enterococcus* bacteria in the blood of these same patients with spontaneous bacterial peritonitis and urinary tract infection.^45^ In the same patients, *Lactobacillales* bacteria in the mucosa of the fundus significantly correlated with ACLF severity (data not shown). Whether these specific phage-bacteria interactions contribute to pathogenesis or whether they are only consequences remains a topic for further mechanistic investigations.

The relationship between increased phage diversity and disease severity, as identified in our study, is a topic of debate since changes in viral diversity in human disease are often conflicting.^36^ While some studies have reported increases in viral diversity in colorectal cancer^54^ and childhood obesity,^55^ viral diversity has been reported to be either not associated or negatively associated with liver disease severity.^18^^,^^47^^,^^56^ Bacterial diversity is negatively associated with liver disease severity,^16^^,^^17^ while phage diversity increases in patients with alcohol-related hepatitis compared to alcohol use disorder,^47^ while cirrhosis^18^ and hepatocellular carcinoma^56^ did not affect phage diversity. These discrepancies might be due to sequencing and/or bioinformatical challenges, such as a failure to account for genome fragmentation,^36^ emphasizing the need for careful comparison of virome results. The increased phage diversity observed in patients with ACLF compared to those with decompensated cirrhosis, together with the decreased bacterial diversity,^17^ could, for example, be attributed to the induction of integrated prophages into their extracellular state due to inflammation^44^ or antibiotic usage,^57^ resulting in phage-mediated bacterial lysis and consequently decreased bacterial diversity. However, many other factors might influence this complex interplay between phages and bacteria.

The highly-personalized nature of fecal viromes, together with the slow temporal drift previously observed in healthy individuals,^43^^,^^58^ is extended in our study to a longitudinal cohort of patients with decompensated cirrhosis and ACLF. However, it should be noted that the inclusion of multiple samples per individual might have introduced bias, particularly affecting the significance of the patient identifier, which was identified as the most important covariate explaining beta-diversity variation. Additionally, the association between inter-individual virome variation and proteins produced by the liver, such as albumin and coagulation factors, reflects the potential effect of liver disease severity on gut virome composition. The gut virome might be indirectly influenced by liver disease severity. For example, altered bile acid metabolism might affect gut bacterial composition,^59^ causing subsequent changes in virome composition. Despite the identification of several explanatory factors, a substantial fraction of inter-sample virome variation remains unexplained, as previously described.^42^ Our study contributed novel insights into virome dynamics, more specifically regarding shifts in phage lifestyles observed in chronic liver disease. In other diseases, such as inflammatory bowel disease, increased levels of temperate phages have been reported.^60^^,^^61^ A potential explanation for the reduced dominance of virulent phages in ACLF might be found in the high levels of systemic inflammation in ACLF,^62^ as this might contribute to the induction of dormant prophages into their extracellular form.^44^ The resulting phage-mediated lysis of bacteria by these induced temperate phages might subsequently enhance the inflammatory response and further contribute to intestinal inflammation.^63^ Notably, our study also linked white blood cell counts, as an inflammatory marker, to phageome composition. This expansion of temperate phages highlights the dynamic nature of the gut virome during the progression of chronic liver disease, but future investigations are warranted to elucidate the mechanisms underlying this shift and its implications for liver disease progression.

Our study comprehensively analyzed longitudinal data in decompensated cirrhosis and ACLF, including the gut virome. In addition, the availability of data on the bacterial part of the microbiome allows for a more sensitive host prediction for the phages and to align the observed phage associations with their bacterial associations. However, the relatively small and heterogeneous cohort limited the statistical power. The etiology of cirrhosis is the covariate with the sixth strongest effect size on the phage variation between patients. Given the different courses of alcohol-related and viral-related liver diseases, the results should be interpreted with caution due to the heterogeneity of our cohort. Moreover, prior use of cephalosporins before inclusion in the study, which could not be accounted for in our analysis, may represent a potential source of bias since its use is recommended by clinical guidelines. Despite these limitations, we were able to validate the association of *Enterococcus B* phages with bacterial infection in patients with decompensated cirrhosis. Due to the scarcity of gut virome studies in patients with decompensated cirrhosis and especially ACLF, no suitable cohorts were available to validate the other findings. Of note, the validation cohort also comprised more patients with an alcohol-related etiology and showed higher MELD and CLIF-C AD scores. Further validation cohorts, also including patients with compensated cirrhosis, are warranted in future studies, to better encompass the natural history of disease and to analyze differences in the gut virome in earlier stages of chronic liver disease. Differences in data analysis, especially regarding phage identification and host prediction methods, further complicate the comparison of our findings with existing literature.

In conclusion, our study represents a first effort in unraveling the role of the gut virome in decompensated cirrhosis and ACLF. We have identified significant changes in virome composition and found associations of phage-host groups with progression and outcome. The study underscores the complexity of the gut virome in human health and disease, and highlights the need for a deeper understanding of virome-bacteriome-host interactions to pave the way for novel clinical applications in the field of hepatology.

## Abbreviations

ACLF, acute-on-chronic liver failure; AD, acute decompensation; CLIF-C, Chronic Liver Failure Consortium; dbRDA, distance-based redundancy analysis; DC = decompensated cirrhosis; EASL-CLIF, European Association for the Study of the Liver – Chronic Liver Failure; MELD, model for end-stage liver disease; TIPS, transjugular intrahepatic portosystemic shunt.

## Financial support

The MICROB-PREDICT project has received funding from the 10.13039/501100000780European Union's 10.13039/501100007601Horizon 2020 research and innovation programme (grant agreement: No 825694). Project 138041 was funded by the Ministry of Innovation and Technology, Hungary, with support from the National Research Development and Innovation Fund, Hungary, under the K grant scheme. The TIPS study was supported by the 10.13039/501100009708Novo Nordisk Foundation (Challenge Grant “MicrobLiver”: NNF15OC0016692). LVE was supported by the Research Foundation 10.13039/501100011878Flanders (FWO: 1S25720N). 10.13039/501100003283MJB is supported by the 10.13039/501100001659Deutsche Forschungsgemeinschaft (DFG, 10.13039/501100001659German Research Foundation) – project ID 493624047 (Clinician Scientist CareerS Münster). JT was supported by the 10.13039/501100001659German Research Foundation (DFG) project ID 403224013 – SFB 1382 (A09), by the German 10.13039/501100002347Federal Ministry of Education and Research (10.13039/501100002347BMBF) for the DEEP-HCC project and by the 10.13039/501100003495Hessian Ministry of Higher Education, Research and the Arts (HMWK) for the ACLF-I cluster project. The DECISION (project ID 847949), GALAXY (project ID 668031), LIVERHOPE (project ID 731875), and IHMCSA (project ID 964590) projects have received funding from the 10.13039/501100000780European Union's 10.13039/501100007601Horizon 2020 research and innovation program. TN is supported by 10.13039/501100001691JSPS KAKENHI (Grant No. 23KJ2059). BS is supported by 10.13039/100000062NIDDK grant P30 DK120515. The computational resources were provided by the 10.13039/100019618Flemish Supercomputer Center (VSC) and funded by 10.13039/501100003130FWO and the 10.13039/501100011878Flemish Government Department of Economy, Science, and Innovation. The funders had no role in study design, data collection and interpretation, or the decision to submit the work for publication.

## Authors’ contributions

Study concept and design; JT, JM, MP, PB, WL; Acquisition of data; MP, BB, LVE, LC, WG; Analysis and interpretation of data; LVE, RS, MJB, LDC, MIK, TN, AF, MK, JT, JM, JSB, BS; Drafting of the manuscript; LVE, MJB, RS, MIK, MK, JT, JM; Critical revision of the manuscript for important intellectual content; all authors; Statistical analysis; LVE, LDC, MK; Obtained funding; JT, MP, PB, JM, WL; Administrative, technical, or material support; LC, WG, RS, AF; Study supervision: JT, JM.

## Data availability

Sequencing data are publicly available at the European Nucleotide Archives (ENA). Their accession numbers are listed in Supplementary Table 12. Any additional information required to reanalyze the data reported in this paper is available from the corresponding author upon request.

## Conflict of interest

JT has received speaking and/or consulting fees from Versantis, Gore, Boehringer-Ingelheim, Falk, Grifols, Genfit and CSL Behring. The other authors have reported no conflict of interest related to this work.

Please refer to the accompanying ICMJE disclosure forms for further details.
