# Supplementary material for: Lactococcus A phages predict ACLF while Enterococcus B phages predict bacterial infection in decompensated cirrhosis
Source: JHEP Rep. 2025 Oct 10;8(1):101622. doi: 10.1016/j.jhepr.2025.101622 (PMC12721046; doi:10.1016/j.jhepr.2025.101622)
Supplement: Multimedia component 1 [file mmc1.pdf]

Enterococcus B and *Lactococcus A* phages are associated with increased mortality in patients with decompensated cirrhosis and ACLF

Lore Van Espen, Maximilian Joseph Brol, Lila Close, Robert Schierwagen, Wenyi Gu, Marisa I Keller, Boglarka Balogh, Anthony Fullam, Lander de Coninck, Tomohiro Nakamura, Michael Kuhn, Peer Bork, Wim Laleman, Jasmohan S Bajaj, Maria Papp, Bernd Schnabl, Jonel Trebicka, Jelle Matthijssens

Table of contents

Supplementary Methods 3
Fig. S1 8
Fig. S2 10
Fig. S3 11
Fig. S4 11
Fig. S5 12
Fig. S6 12
Fig. S7 13
Fig. S8 14
Fig. S9 14
Fig. S10 15
Fig. S11 16
Table S1 17
Table S2 18
Table S3 18
Table S4 19
Table S5 20
Table S6 20
Table S7 21
Table S8 21
Table S9 22
Table S10 22
Table S11 23

Table S12 23
Supplementary References 30

Supplementary methods

Sample preparation for virome sequencing

Viral-like particles were purified from fecal samples and prepared for virome sequencing using the NetoVIR protocol[1]. In short, fecal aliquots were homogenized in PBS (10 m/V%) using a Precellys homogenizer (Bertin technologies, 5,000 rpm for 15 s), centrifuged (17,000 g for 3 min), filtered (0.8 µm PES filter, Sartorius) and treated with a mixture of benzonase (Novogen) and micrococcal nuclease (New England Biolabs) to enrich for viral-like particles. Both RNA and DNA were extracted using the Viral RNA Mini kit (Qiagen) without carrier RNA. Extracts were reverse transcribed and randomly amplified (17 cycles) using a modified WTA2 kit (Sigma Aldrich) and purified using the MSB Spin PCRapace purification kit (Stratec). Sequencing libraries were prepared with the Nextera XT DNA Library Preparation kit (Illumina), quantified with the Qubit dsDNA High Sensitivity kit (Thermo Fisher Scientific) and the insert length was determined using the Bioanalyzer2000 and the High Sensitivity DNA kit (Agilent). Libraries were sequenced (2 x 150 bp) on the NextSeq500 Illumina platform (Nucleomics Core facility, KU Leuven, Belgium). Seven samples were not sequenced due to too low concentrations after random amplification or library preparation.

Quality control and *de novo* assembly of virome dataset

Raw reads were trimmed using Trimmomatic (v0.36)[2] to remove primers/adaptors (parameters: 30:10:1:true) and remove bad-quality (parts of) reads (parameters: HEADCROP:19 LEADING:15 TRAILING:15 SLIDINGWINDOW:4:20 MINLEN:50). Quality filtered reads were *de novo* assembled using metaSPAdes (v3.11.1, kmer sizes of 21, 33, 55 and 77)[3]. To overcome the issue of highly covered but fragmented genomes, the *de novo* assembly was performed on subsets of 100%, 10% and 1% of the quality-filtered reads generated by seqkit (v0.10.0)[4]. Per sample, the resulting scaffolds > 1 kb were clustered to remove redundancy across all three assemblies at 99% ANI over 99% coverage using BLASTn (v2.5.0+)[5] and the anicalc.py and aniclust.py scripts as described on the CheckV github (v0.7.0)[6]. Afterwards, all remaining scaffolds were clustered across samples at 95% ANI over 85% coverage to remove cross-sample redundancy.

Identification of viral scaffolds

Human scaffolds were identified by Kraken2 (v2.1.1, database downloaded on 2021/05/20, parameters: --confidence 0.5)[7]. The non-redundant scaffold set was compared against the NCBI nucleotide dataset (version of 2021/05/14) and protein dataset (downloaded on 2021/05/20) using BLASTn (v2.10.0+, parameters: -evalue 1e-10)[5] and DIAMOND (v2.0.9, parameters: --sensitive)[8], respectively. The lowest common ancestor (LCA) of the top nucleotide and protein hits were determined using ktClassifyBLAST from KronaTools (v2.7.1, database downloaded on 2021/05/20)[9]. CheckV (v0.8.1, database v1.0)[6] was used to determine completeness of viral genomes. Scaffolds were classified as eukaryotic viruses if the DIAMOND LCA was a eukaryotic virus. If the DIAMOND LCA was "Root" or if the CheckV completeness estimate was ≥ 50% and the DIAMOND LCA was no virus, the BLASTn LCA was used for annotation instead (if this was a eukaryotic virus). Phages were identified by VirSorter2 (v2.2.2, database downloaded on 2021/02/03)[10] including the ssDNA, dsDNAphage and RNA viral groups (no score cut-off) and required a completeness estimate by CheckV (i.e. not "Not-determined") to be selected. Members of the *Inoviridae* phage family were selected based on DIAMOND LCA as both VirSorter2 and CheckV are less suited to detect this group of viruses well. The analysis of the phageome will focus on the "good-quality" phages (i.e. estimated to be ≥ 50% complete by CheckV[6] or length ≥ 4 kb for phages of the *Inoviridae* family) (4% of phage scaffolds; 79.4% phage reads; Fig. 1B) to reduce the noise resulting from highly fragmented genomes.

Abundance determination for virome dataset

Instead of determining abundances by mapping quality-filtered reads per sample to the entire non-redundant scaffold set, reads were only mapped against the representatives of the clusters containing a scaffold from that sample, to avoid false positive detection of closely related sequences, using BWA-MEM2 (v2.2.1)[11]. A scaffold was assumed to be present if 70% of its length was horizontally covered by reads as determined by samtools depth (v1.7)[12]. Decontam (v1.10.0)[13] was used to identify contaminating scaffolds in the extraction and amplification controls separately, using the prevalence mode (0.5 score threshold) which relies on the assumption that contamination has a higher prevalence in controls than in samples. Afterwards, remaining non-contaminating viral reads were random subsampled to a depth of

329,569 viral reads using the "rarefy_even_depth" function from Phyloseq (v1.34)[14], removing ten samples from subsequent analyses with less viral reads.

Taxonomical classification of viral scaffolds

Since phage taxonomy is a rapidly evolving field and the large majority of phage sequences are unclassified to begin with, "good-quality" phages are clustered into groups that roughly represent genus and family levels based on gene sharing (minimal 20% or 16 genes and 10% or 8 genes, respectively) and average amino-acid identity (minimal 40 and 20%, respectively) as described previously [15]. Open-reading frames used to perform this clustering were predicted using Prodigal (v2.6.3)[16] in metagenomic mode. A taxonomic group was assigned to clusters including RefSeq phage genomes (v209; n = 4,733)[17] (i.e. crassphages, non-crass caudoviruses, inoviruses and microviruses).

Processing of fecal bacteriome dataset

DNA was extracted from stool samples using Qiagen AllPrep Power Fecal DNA/RNA Kit. Metagenomic sequencing libraries were prepared using the NEBNext Ultra II DNA Library Prep kit with a targeted insert size of 350 – 400 bp and Dual Index multiplex oligos. Libraries were prepared using liquid automated systems (Beckman Coulter i7 Series) and sequenced on an Illumina HiSeq 4000 platform (Illumina, San Diego, CA, USA) with 2 x 150 bp paired-end reads. Metagenomic data was processed to remove low quality data using NGLess (v1.1)[18]. Nucleotide calls with a Phred score of less than 25 were removed from the 3' end. Any reads that were less than 45 nucleotides long after removal of low-quality nucleotide calls were discarded. Any reads with greater than 90 % sequence similarity to the human reference genome (hg38) were discarded. Reads were re-classified as paired or as singles, where, respectively, both or only the forward and reverse reads are present in the final dataset.

Phage host and lifestyle prediction

Good-quality phages were linked to putative hosts using a combination of methods relying on two host databases. The first host database contains the metagenome-assembled genomes (MAGs) from whole-genome shotgun metagenomic sequences of the same fecal samples (n = 15,543). Assembly of quality-controlled metagenomic reads was performed using megahit (v1.2.9)[19] followed by binning using metabat (v2.12.1)[20]. The second host database contains bacterial species representatives (n =

4,744) from the Unified Human Gastrointestinal Genomes (UHGG) database (v2.0)[21]. Both databases have been taxonomically annotated using the GTDB taxonomy (v202) using GTDB_Tk (v1.5.0)[22].

Each phage – host genus pair was scored based on prophage matches, CRISPR spacer matches, tRNA matches, PHIST[23] and RaFAH[24] and every pair with a score of three of more (see below) was considered a putative host genus for that phage.

(I) Prophage matches: Phage genomes were compared to both MAG databases using BLASTn (v2.0.5+, parameters: -perc_identity 90)[5]. ANI, query coverage (qcov) and target coverage (tcov) between phage and MAG contig were calculated using the anicalc.py script from the CheckV github[6] and hits ≤ 90% ANI or > 70% tcov were removed (to require at least 30% of the host genome outside of the prophage region to avoid hits to misbinned phage genomes in the MAGs as described in Gregory & Zablocki, 2022[25]). A phage – host genus pair with one prophage match with > 30% qcov received two points, while two matches with > 30% qcov or one match with > 70% qcov received three points.

(II & III) CRISPR & tRNA matches: CRISPR spacers and tRNAs were predicted on both MAG datasets using respectively MinCED (v0.4.2)[26] and Aragorn (v1.2.41)[27]. CRISPR spacers were compared against the phages using BLASTn (v2.0.5+, parameters: --task blastn-short)[5] and tRNAs against the tRNAs predicted on the good-quality phages by Aragorn (v1.2.41)[27] using BLASTn (v2.0.5+)[5]. Only hits without gaps and spanning at least 95% of the CRISPR spacer or 97% of the tRNA gene were considered. A phage – host genus pair with a CRISPR or tRNA match with maximum 1 mismatch received one point, while a CRISPR or tRNA match without mismatch resulted in two points for the CRISPR and tRNA scores respectively.

(IV) PHIST: PHIST (v1.0.0)[23] was run on the good-quality phage contigs and both MAG datasets. A phage – host genus pair with a PHIST adj. p < 0.001 received one point, while a PHIST adj. p < 0.000001 resulted in two points.

(V) RaFAH: RaFAH (v0.3)[24] was run on the good-quality phage contigs (no need for MAG input). The RaFAH genera were converted to GTDB genera to align with the MAG taxonomical classification and every phage – host genus with a RaFAH score > 0.1 received one point, pairs with a RaFAH score > 0.5 received two points and pairs with a RaFAH score > 0.8 received three points.

The lifestyle of the good-quality phages was predicted using BACPHLIP (v0.9.6)[28]. A phage with a BACPHLIP temperate score > 0.5 or a prophage match to a MAG from either datasets (> 90% ANI, ≤ 70% tcov and > 30% qcov) was classified as temperate. Additionally, phages part of predominantly temperate phages with an estimated completeness > 90% will be classified as temperate phages. All other good-quality phages were classified as virulent.

Analysis of fecal bacteriome dataset

Metagenomes were analyzed using mOTUs (v2.5)[29], which requires the identification of at least three marker genes for reliable taxonomic profiling, adhering to NCBI taxonomy. mOTU taxonomical classifications were transformed into GTDB taxonomy to align with the bacterial taxonomy used for phage host prediction. We used GTDB-tk on ProGenomes2 database genomes to assign taxonomy for marker genes defining mOTUs[22,30]. Out of 11,699 clusters, 750 displayed taxonomic inconsistencies at the species level. We set a threshold of 80% consistency for taxonomic assignments within conflict clusters and excluded unknown results. For unresolved cases, we noted multiple genera to mark ambiguous taxonomy.

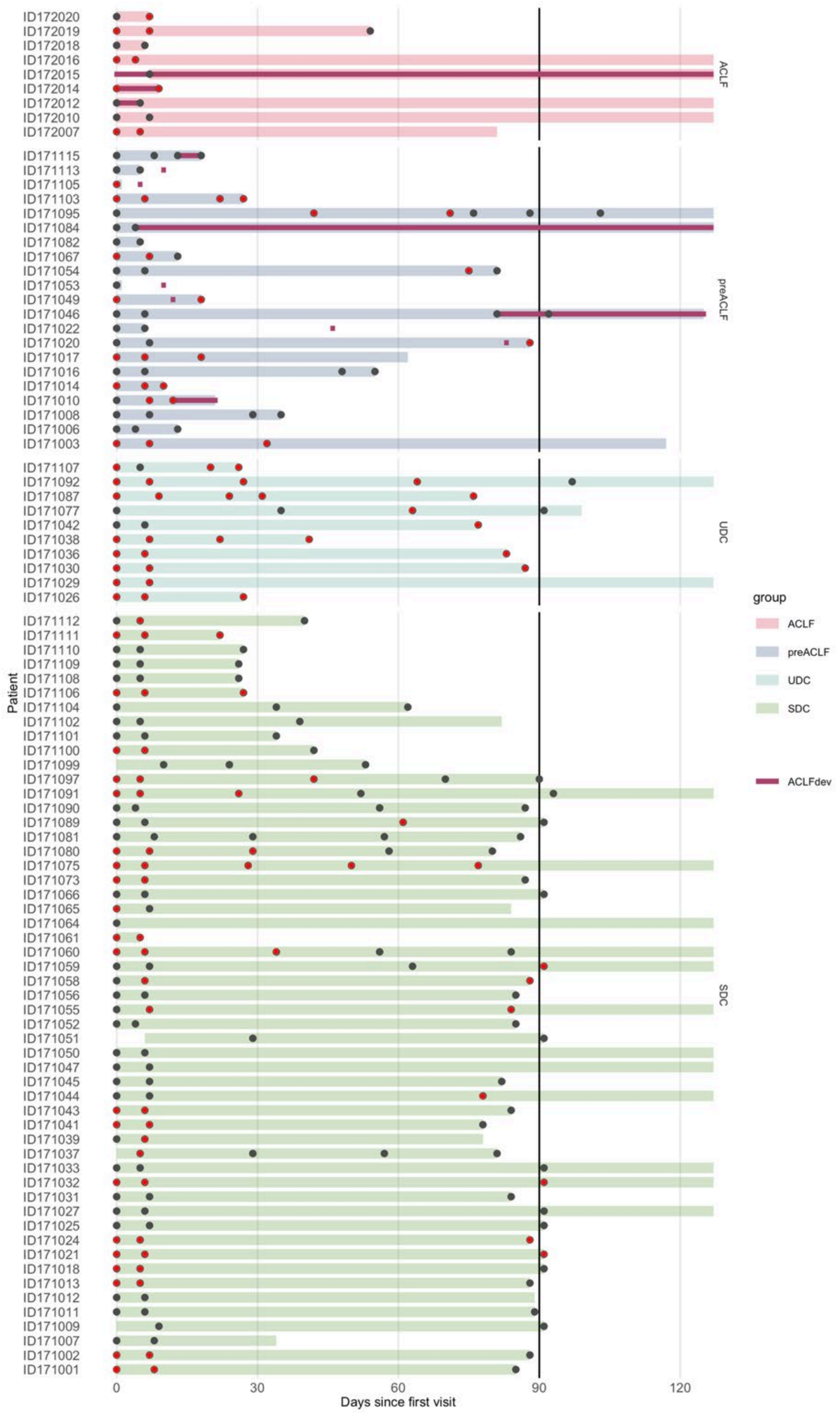
ID172020
ID172019
ID172018
ID172016
ID172015
ID172014
ID172012
ID172010
ID172007
ACLF
ID171115
ID171113
ID171105
ID171103
ID171095
ID171084
ID171082
ID171067
ID171054
ID171053
ID171049
ID171046
ID171022
ID171020
ID171017
ID171016
ID171014
ID171010
ID171008
ID171006
ID171003
preACLF
ID171107
ID171092
ID171087
ID171077
ID171042
ID171038
ID171036
ID171030
ID171029
ID171026
UDC
ID171112
ID171111
ID171110
ID171109
ID171108
ID171106
ID171104
ID171102
ID171101
ID171100
ID171099
ID171097
ID171091
ID171090
ID171089
ID171081
ID171080
ID171075
ID171073
ID171066
ID171065
ID171064
ID171061
ID171060
ID171059
ID171058
ID171056
ID171055
ID171052
ID171051
ID171050
ID171047
ID171045
ID171044
ID171043
ID171041
ID171039
ID171037
ID171033
ID171032
ID171031
ID171027
ID171025
ID171024
ID171021
ID171018
ID171013
ID171012
ID171011
ID171009
ID171007
ID171002
ID171001
SDC
Patient
0
30
60
90
120
Days since first visit
group
ACLF
preACLF
UDC
SDC
ACLFdev

Fig. S1: Overview of longitudinal MUCOSA-PREDICT cohort.

Lines represent the follow-up period of all patients colored by disease group. Dots indicate visits included in virome analysis (visits after 120 days (n = 11) are excluded from this figure for clarity). Purple indicates the presence of ACLF in these patients. Red dots indicate presence of bacterial infection at visit. Black line represents the 90-day follow-up period used to stratify patients with AD into pre-ACLF, unstable and stable decompensated cirrhosis groups. ACLF = acute-on-chronic liver failure; AD = acute decompensation; SDC = stable decompensated cirrhosis; UDC = unstable decompensated cirrhosis.

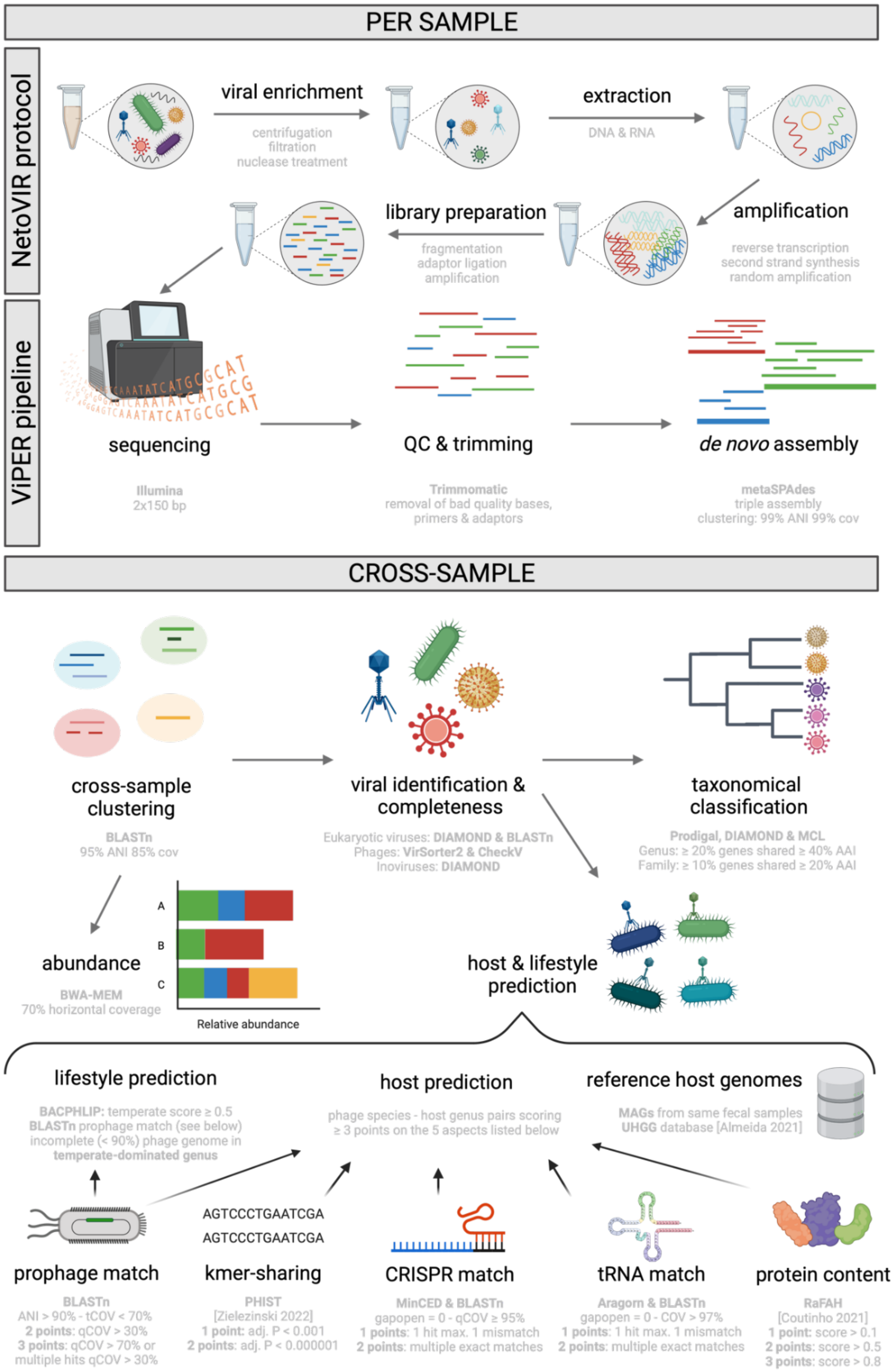

Fig. S2: Overview of fecal sample preparation and bioinformatic processing for virome profiling.

Overview of different steps in the sample preparation, sequencing and bioinformatic processing of the fecal samples for virome profiling. Created with BioRender.

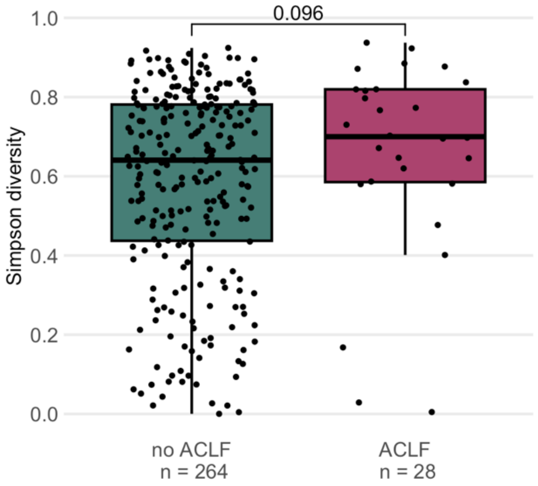

Fig. S3: Phage Simpson diversity associated with ACLF.

Comparison of phage alpha-diversity in samples with and without ACLF (Wilcoxon rank-sum test). ACLF = acute-on-chronic liver failure.

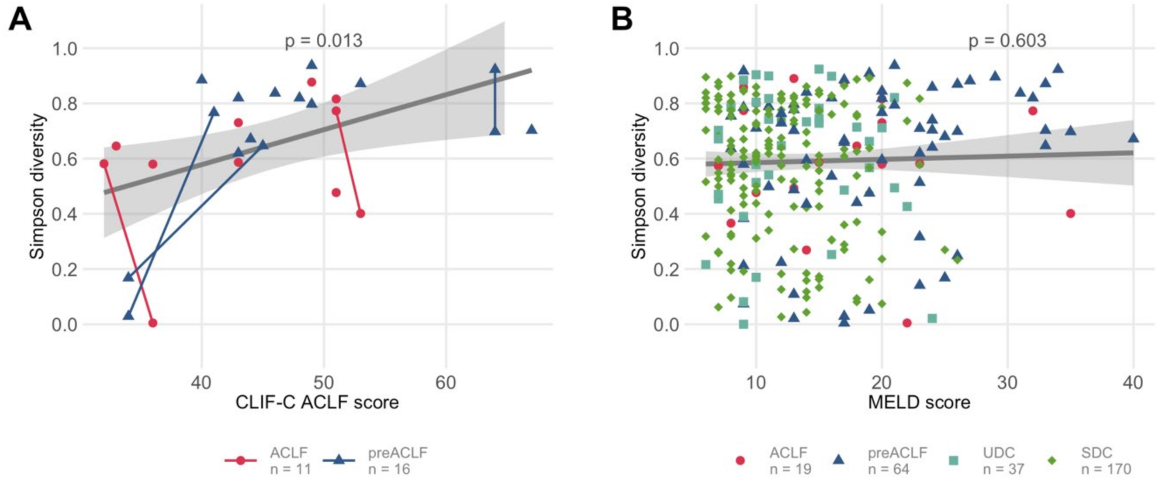

Fig. S4: Phage Simpson diversity associated with CLIF-C ACLF, but not MELD score

(A) Relationship between phage alpha-diversity and CLIF-C ACLF score across all ACLF visits of all (pre)ACLF patients (Pearson's correlation coefficient = 0.47). Samples from the same patient are connected. (B) Relationship between phage alpha-diversity and MELD score across all visits of all patients (Pearson's correlation coefficient = 0.03 (ns)). ACLF = acute-on-chronic liver failure; UDC = unstable decompensated cirrhosis; SDC = stable decompensated cirrhosis.

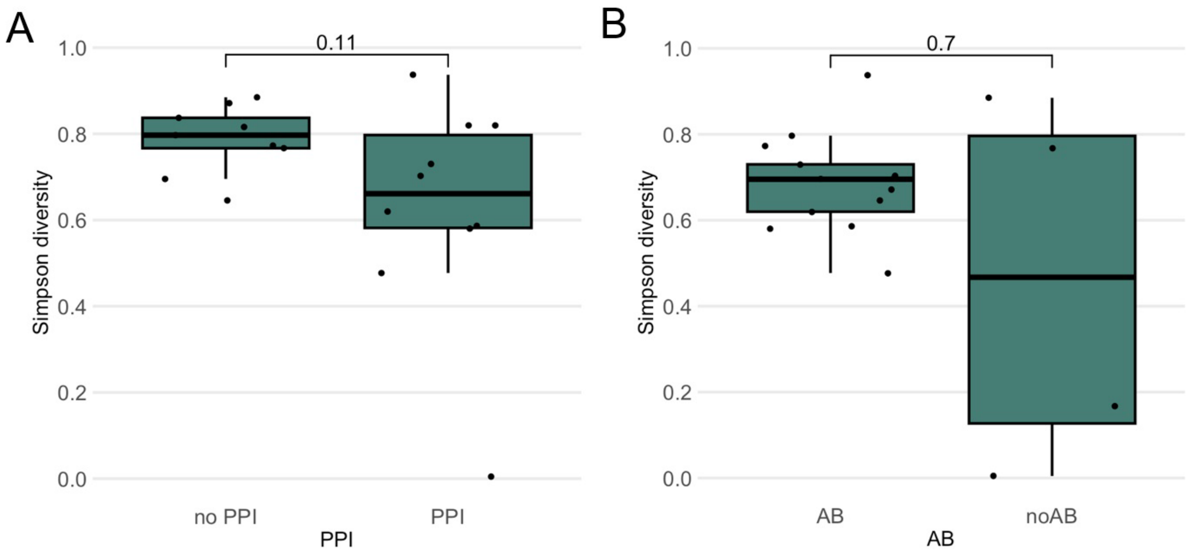

Fig. S5: Phage Simpson diversity in relation to proton pump inhibitor and antibiotic use. (A) Comparison of phage alpha-diversity in samples from patients with ACLF with or without concurrent use of PPI (Wilcoxon rank-sum test). (B) Comparison of phage alpha-diversity in samples from patients with ACLF with or without concurrent use of antibiotics (Wilcoxon rank-sum test). AB = antibiotics; PPI = proton pump inhibitors; ACLF = acute-on-chronic liver failure.

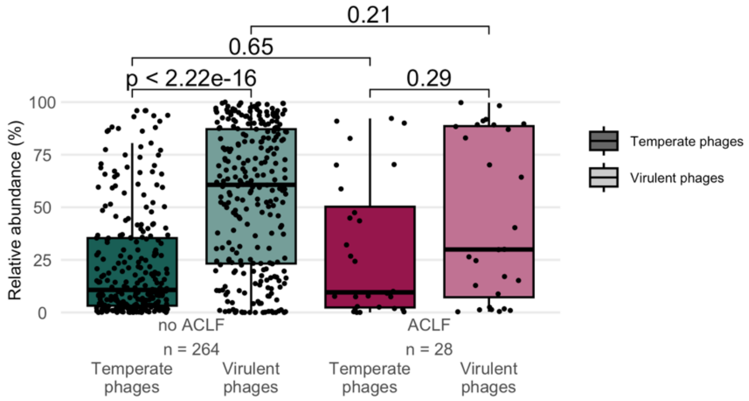

Fig. S6: Phageome composition in decompensated cirrhosis and ACLF. Phageome composition in terms of phage lifestyle in all samples with and without ACLF (Wilcoxon rank-sum test). ACLF = acute-on-chronic liver disease.

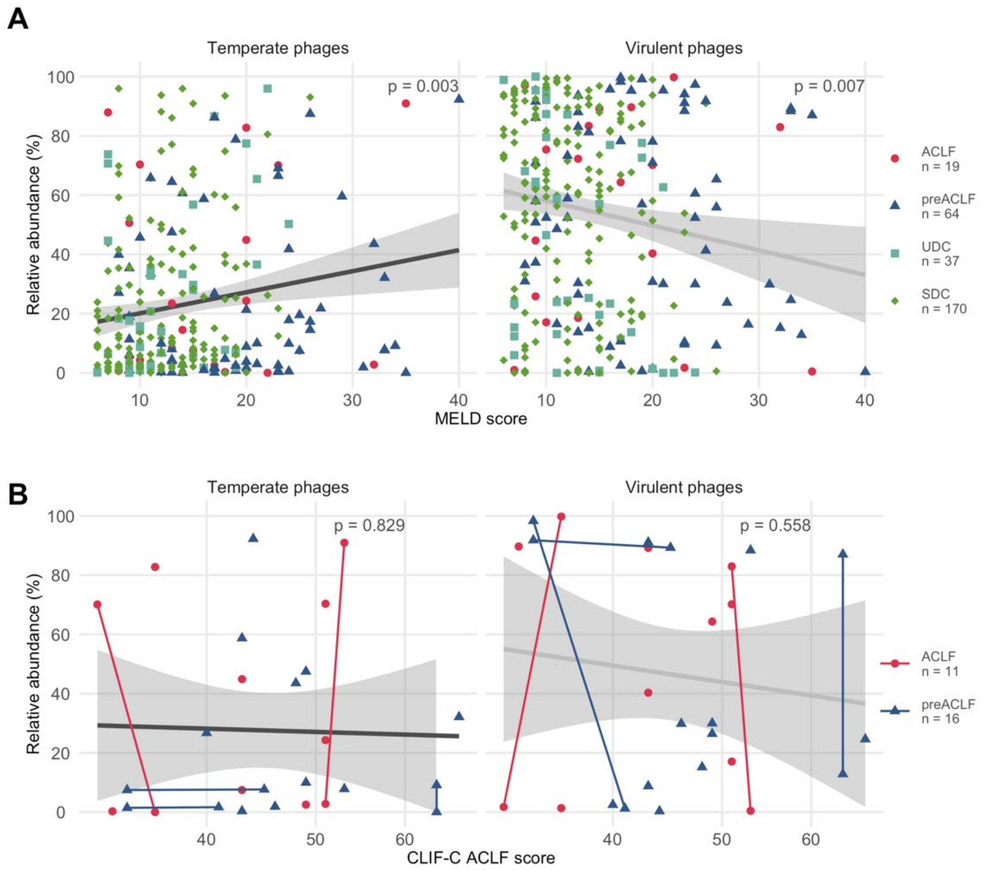

Fig. S7: Phage Simpson diversity associated with CLIF-C ACLF, but not MELD score

(A) Relationship between the relative abundance of temperate and virulent phages and MELD score across all visits of all patients (Pearson's correlation coefficient = 0.17 and -0.17). (B) Relationship between the relative abundance of temperate and virulent phages and CLIF-C ACLF score across all ACLF visits of all (pre)ACLF patients (Pearson's correlation coefficient = -0.04 (ns) and -0.12 (ns)). Samples from the same patient are connected.

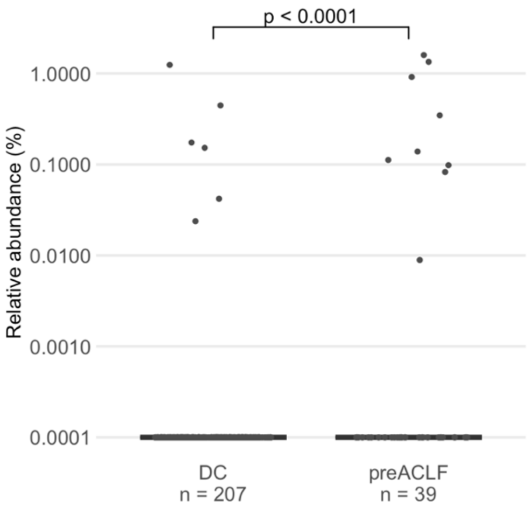

Fig. S8: *Lactococcus A* phages associated with pre-ACLF.

Relative abundance of *Lactococcus A* phages in the all samples of patients with decompensated cirrhosis vs. all samples of pre-ACLF patients before ACLF onset (Wilcoxon rank-sum test). DC = decompensated cirrhosis; ACLF = acute-on-chronic liver failure.

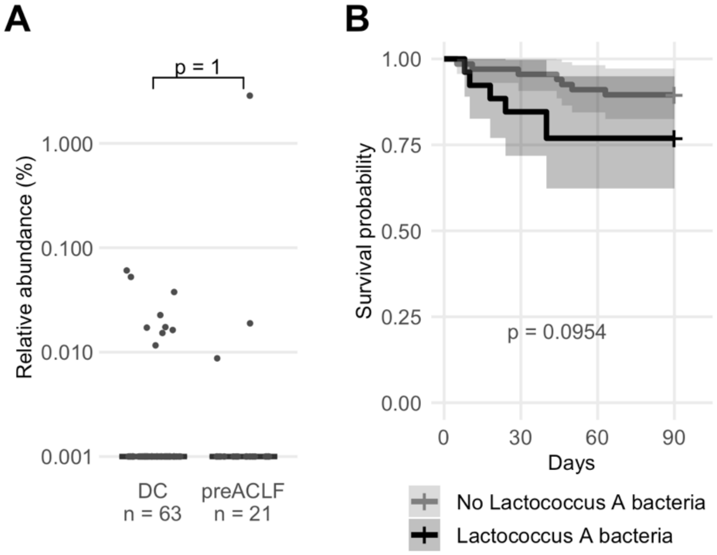

Fig. S9: *Lactococcus A* bacteria associated with higher short-term mortality, but not pre-ACLF.

(A) Relative abundance of *Lactococcus A* bacteria in the first visit of decompensated cirrhosis vs. pre-ACLF patients (Wilcoxon rank-sum test). (B) 90-day survival analysis of patients with and without *Lactococcus A* bacteria (first sample with these bacteria within 90 days from first sample; n = 26 vs.

first sample; n = 67) (Log-rank test). DC = decompensated cirrhosis; ACLF = acute-on-chronic liver failure.

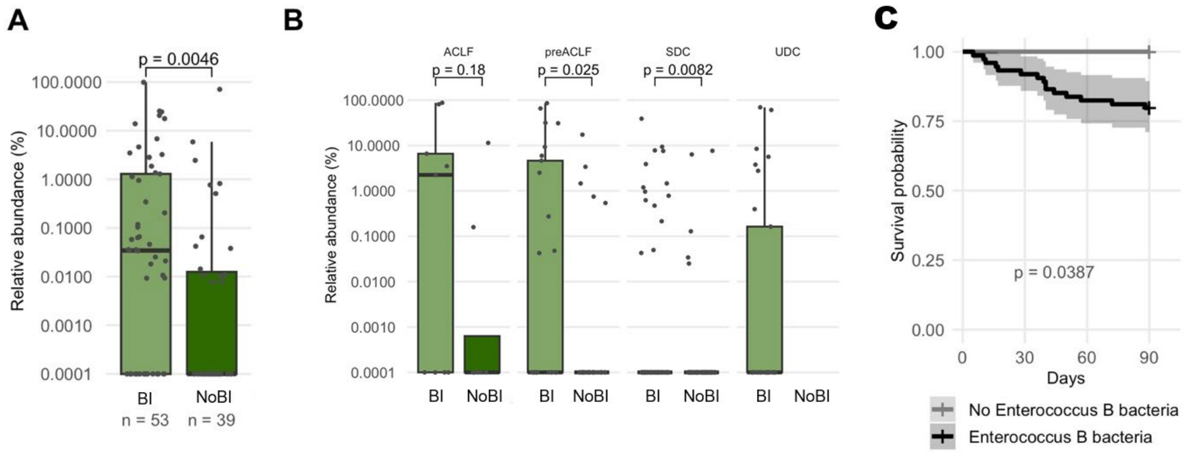

Fig. S10: *Enterococcus B* bacteria associated with bacterial infection and worse short-term survival.

(A) Relative abundance of *Enterococcus B* bacteria in patients with and without bacterial infection (Wilcoxon rank-sum test). (B) Relative abundance of *Enterococcus B* bacteria in patients with and without bacterial infection in patients with ACLF, preACLF, UDC and SDC (ACLF = acute-on-chronic liver failure; UDC = unstable decompensated cirrhosis; SDC = stable decompensated cirrhosis.) (C) 90-day survival analysis of patients with (n = 74) and without (n = 19) *Enterococcus B* bacteria (first sample with these bacteria within 90 days from first sample vs. first sample) (Log-rank test).

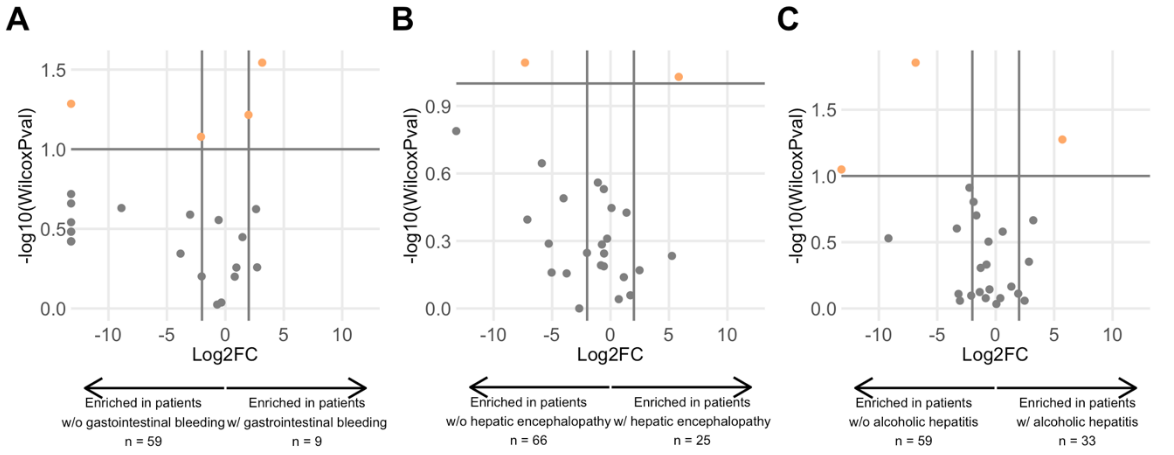

Fig. S11: Phage-host groups are not associated with any other precipitating events.

(A) Volcano plots to visualize changes in abundance of phage-host groups (n = 24) between patients with and without gastrointestinal bleeding present in at least 5/68 samples (first sample with gastrointestinal bleeding vs. first sample of patients without gastrointestinal bleeding). (B)

Volcano plots to visualize changes in abundance of phage-host groups (n = 25) between patients with and without hepatic encephalopathy present in at least 5/91 samples (first sample with hepatic encephalopathy vs. first sample of patients without hepatic encephalopathy). (C) Volcano plots to visualize changes in abundance of phage-host groups (n = 25) between patients with and without alcoholic hepatitis present in at least 5/92 samples (first sample with alcoholic hepatitis vs. first sample of patients without alcoholic hepatitis). (Wilcoxon rank-sum tests; orange dots = $p < 0.1$ and FDR < 0.1).

Supplementary Tables

Variable	Category	Univariate dbRDA					Multivariate dbRDA (n = 268)		
		n	R2	AdjR2	P	AdjP	AdjR2	AdjR2_0	P
ACLF	Disease	292	0.00379472	0.00035953	0.381	0.41189	NA	NA	NA
Active Alcoholism	Demographic	292	0.00332125	-0.0001156	0.64	0.64000	NA	NA	NA
Age	Demographic	292	0.00589354	0.00246558	0.008	**0.01778**	NA	NA	NA
Albumin - blood (sqrt)	Lab_Liver	285	0.00676459	0.00325492	0.001	**0.00400**	0.3244268	0.00411362	0.002
Albumin - medication	Medication	292	0.00545382	0.00202435	0.027	**0.04320**	NA	NA	NA
ALP (log)	Lab_Liver	284	0.00596202	0.00243706	0.019	**0.03455**	NA	NA	NA
ALT (log)	Lab_Liver	286	0.0055382	0.00203657	0.024	**0.04000**	NA	NA	NA
Antibiotics	Medication	292	0.00675498	0.00333	0.002	**0.00533**	NA	NA	NA
Ascites	Disease	292	0.00479708	0.00136535	0.061	**0.08414**	NA	NA	NA
Alcholic steatohepatitis	Disease	292	0.0041072	0.00067309	0.238	0.28000	NA	NA	NA
AST (log)	Lab_Liver	287	0.00630497	0.00281832	0.001	**0.00400**	NA	NA	NA
Bilirubin (log)	Lab_Liver	292	0.00704219	0.0036182	0.002	**0.00533**	NA	NA	NA
Bacterial infection	Disease	292	0.00726626	0.00384304	0.001	**0.00400**	NA	NA	NA
BMI	Demographic	292	0.00393887	0.00050417	0.309	0.35314	NA	NA	NA
Cardiologic failure	Disease	292	0.00382875	0.00039368	0.348	0.38667	NA	NA	NA
Cerebral failure	Disease	292	0.00347652	4.0236E-05	0.535	0.56316	NA	NA	NA
Coagulatory failure	Disease	292	0.00451563	0.00108293	0.126	0.15750	NA	NA	NA
Creatinine (log)	Lab_Liver	291	0.00476581	0.00132209	0.074	**0.09548**	NA	NA	NA
CRP (log)	Lab_Inflammation	292	0.00668853	0.00326332	0.004	**0.01000**	NA	NA	NA
Etiology	Disease	292	0.02543169	0.00839378	0.001	**0.00400**	NA	NA	NA
Gastrointestinal failure	Disease	292	0.00412347	0.00068941	0.23	0.27879	NA	NA	NA
GGT (log)	Lab_Liver	276	0.00541847	0.00178861	0.048	**0.07111**	NA	NA	NA
Group	Disease	292	0.01674557	0.00650334	0.001	**0.00400**	NA	NA	NA
Hepatic encephalopathy	Disease	292	0.00581041	0.00238217	0.011	**0.02095**	NA	NA	NA
PatientID	Demographic	292	0.52857428	0.31062872	0.001	**0.00400**	0.3203132	0.32031318	0.001
INR (inv)	Lab_Liver	290	0.00563943	0.00218679	0.024	**0.04000**	0.3275784	0.00315162	0.014
Liver dysfunction	Disease	292	0.00628629	0.00285969	0.007	**0.01647**	NA	NA	NA
Lymphocytes (log)	Lab_Inflammation	292	0.0048527	0.00142115	0.061	**0.08414**	NA	NA	NA
MELD score	Disease	290	0.00779135	0.00434618	0.002	**0.00533**	NA	NA	NA
MELD-Na score	Disease	290	0.00845563	0.00501277	0.001	**0.00400**	NA	NA	NA
Monocytes (log)	Lab_Inflammation	292	0.00612467	0.00269751	0.011	**0.02095**	NA	NA	NA
Sodium	Lab_Kidney	291	0.00952583	0.00609858	0.001	**0.00400**	NA	NA	NA
Neutrophils (log)	Lab_Inflammation	292	0.00738795	0.00396515	0.002	**0.00533**	NA	NA	NA
Protein-pump inhibitors	Medication	292	0.00661151	0.00318603	0.002	**0.00533**	NA	NA	NA
Renal dysfunction	Disease	291	0.00670037	0.00326334	0.001	**0.00400**	NA	NA	NA
Rifaximin	Medication	292	0.00506184	0.00163101	0.044	**0.06769**	NA	NA	NA
Sex	Demographic	292	0.00601041	0.00258286	0.009	**0.01895**	NA	NA	NA
Lactulose	Medication	292	0.00336686	-6.981E-05	0.613	0.62872	NA	NA	NA
Visit Day	Demographic	292	0.00485722	0.00142569	0.064	**0.08533**	0.3322015	0.00231562	0.049
WBC (log)	Lab_Inflammation	292	0.00768504	0.00426327	0.001	**0.00400**	0.3298859	0.0023075	0.02

Table S1: Individual effect size of each covariate for determing the high inter-sample diversity in phage community using univariate distance-based redundancy analysis (dbRDA).

Host_Genus	Prevalence				Fisher test for equal proportions			Wilcoxon rank-sum test		
	preACLF (n = 21)		SDC & UDC (n = 63)		OR	P	AdjP	Log2FC	P	AdjP
	n	%	n	%						
Alistipes	8	38.1%	22	34.9%	1.145	0.798	1.000	-1.097	0.759	0.976
Bacteroides	10	47.6%	34	54.0%	0.778	0.625	1.000	-2.488	0.388	0.976
Bifidobacterium	4	19.0%	6	9.5%	2.211	0.259	1.000	2.085	0.283	0.976
Blautia_A	0	0.0%	7	11.1%	0.000	0.184	1.000	-Inf	0.116	0.961
Chlamydia	4	19.0%	17	27.0%	0.640	0.570	1.000	-2.168	0.674	0.976
Dysosmobacter	2	9.5%	6	9.5%	1.000	1.000	1.000	-1.064	0.976	0.976
Enterocloster	2	9.5%	11	17.5%	0.501	0.502	1.000	0.593	0.403	0.976
Enterococcus_B	3	14.3%	7	11.1%	1.329	0.705	1.000	-4.746	0.854	0.976
Escherichia	4	19.0%	17	27.0%	0.640	0.570	1.000	1.175	0.625	0.976
Faecalibacterium	5	23.8%	15	23.8%	1.000	1.000	1.000	-2.849	0.945	0.976
Flavonifractor	3	14.3%	8	12.7%	1.144	1.000	1.000	-0.866	0.846	0.976
Fusicatenibacter	1	4.8%	4	6.3%	0.740	1.000	1.000	-6.301	0.762	0.976
Gemmiger	1	4.8%	5	7.9%	0.583	1.000	1.000	0.135	0.644	0.976
Klebsiella	2	9.5%	7	11.1%	0.844	1.000	1.000	2.061	0.908	0.976
Lactococcus	6	28.6%	20	31.7%	0.862	1.000	1.000	1.759	0.895	0.976
Lactococcus_A	4	19.0%	1	1.6%	14.001	0.013	0.322	7.090	0.003	**0.080**
Lawsonibacter	2	9.5%	3	4.8%	2.084	0.595	1.000	-2.733	0.450	0.976
Leuconostoc	5	23.8%	7	11.1%	2.469	0.164	1.000	0.784	0.164	0.961
Mediterraneibacter	0	0.0%	8	12.7%	0.000	0.192	1.000	-Inf	0.090	0.961
Odoribacter	3	14.3%	9	14.3%	1.000	1.000	1.000	-4.011	0.919	0.976
Parabacteroides	8	38.1%	20	31.7%	1.319	0.603	1.000	-0.131	0.763	0.976
Prevotella	2	9.5%	13	20.6%	0.409	0.336	1.000	0.730	0.330	0.976
Ruminococcus_B	1	4.8%	5	7.9%	0.583	1.000	1.000	-2.548	0.627	0.976
Streptococcus	4	19.0%	7	11.1%	1.867	0.455	1.000	-3.794	0.449	0.976
Veillonella	1	4.8%	10	15.9%	0.268	0.277	1.000	-3.696	0.192	0.961

Table S2: Phage-host groups in patients with preACLF compared to patients with SDC and UDC. ACLF = acute-on-chronic liver failure; UDC = unstable decompensated cirrhosis; SDC = stable decompensated cirrhosis.

Length	Estimated completeness	Genus	Family	Host prediction method (exc. Prophage)	Prophage species hits	BACPHLIP temperate	Lifestyle
19 kb	51%	Caudoviricetes	Family_2	PHIST + tRNA		Yes	Temperate
19 kb	56%	Caudoviricetes	Family_2	PHIST	L. laudensis (n = 1)	No	Temperate
* Species level host prediction only possible if prophage hit to a high-quality bacterial MAG.							

Table S3: Temperateness of *Lactococcus A* phages associated with future ACLF development

Host_Genus	Group sizes (n)		Observed events (n)		Expected events (n)		Cox regression model		
	Absent	Present	Absent	Present	Absent	Present	ChiSq	P	AdjP
Alistipes	54	39	7	6	7.454	5.546	0.065	0.799	0.940
Anaerobutyricum	88	5	13	0	12.252	0.748	0.795	0.373	0.638
Anaerostipes	88	5	13	0	12.252	0.748	0.795	0.373	0.638
Bacteroides	31	62	5	8	4.200	8.800	0.225	0.635	0.819
Barnesiella	88	5	12	1	12.347	0.653	0.194	0.659	0.824
Bifidobacterium	78	15	9	4	11.063	1.937	2.590	0.108	0.450
Blautia_A	79	14	12	1	10.906	2.094	0.683	0.409	0.654
Chlamydia	69	24	11	2	9.622	3.378	0.761	0.383	0.638
Clostridium_AQ	84	9	11	3	12.709	1.291	2.499	0.114	0.450
Dorea_A	84	9	11	4	13.659	1.341	5.823	0.016	0.209
Dysosmobacter	83	10	10	3	11.727	1.273	2.604	0.107	0.450
Enterobacter	86	7	13	0	11.953	1.047	1.141	0.285	0.616
Enterocloster	67	26	11	3	9.895	4.105	0.422	0.516	0.774
Enterococcus	88	5	11	2	12.349	0.651	2.951	0.086	0.450
Enterococcus_B	57	36	4	12	10.183	5.817	10.389	0.001	**0.025**
Erysipelatoclostridium	83	10	13	0	11.504	1.496	1.694	0.193	0.515
Escherichia	59	34	9	4	8.077	4.923	0.278	0.598	0.797
Faecalibacterium	64	29	9	4	8.806	4.194	0.013	0.908	0.981
Faecalimonas	85	8	13	0	11.804	1.196	1.321	0.250	0.589
Flavonifractor	71	22	12	1	9.710	3.290	2.140	0.144	0.450
Fusicatenibacter	88	5	12	1	12.252	0.748	0.090	0.764	0.926
Gemmiger	86	7	12	1	12.059	0.941	0.004	0.950	0.981
Hungatella	87	6	11	2	12.222	0.778	2.045	0.153	0.450
Klebsiella	73	20	9	5	11.231	2.769	2.241	0.134	0.450
Lactobacillus	85	8	13	0	11.804	1.196	1.321	0.250	0.589
Lactococcus	33	60	4	10	4.999	9.001	0.311	0.577	0.796
Lactococcus_A	80	13	6	7	11.564	1.436	24.393	0.000	**0.000**
Lawsonibacter	81	12	12	1	11.300	1.700	0.332	0.564	0.796
Leuconostoc	62	31	5	8	8.872	4.128	5.336	0.021	0.209
Massilioclostridium	88	5	11	2	12.453	0.547	4.039	0.044	0.356
Mediterraneibacter	81	12	12	1	11.205	1.795	0.409	0.522	0.774
Odoribacter	73	20	10	3	10.081	2.919	0.003	0.957	0.981
Parabacteroides	56	37	8	5	7.865	5.135	0.006	0.939	0.981
Prevotella	69	24	10	4	10.330	3.670	0.040	0.841	0.961
Roseburia	87	6	11	2	12.222	0.778	2.047	0.152	0.450
Rothia	88	5	13	0	12.252	0.748	0.795	0.373	0.638
Ruminococcus_B	86	7	12	2	13.009	0.991	1.107	0.293	0.616
Ruminococcus_E	87	6	13	0	12.103	0.897	0.966	0.326	0.638
Streptococcus	71	22	8	5	10.114	2.886	1.998	0.158	0.450
Veillonella	71	22	10	3	10.015	2.985	0.000	0.992	0.992

Table S4: Association between short-term (90-day) mortality and the presence of phage-host groups

Host_Genus	Prevalence				Fisher test for equal proportions			Wilcoxon rank-sum test		
	Bacterial infection (n = 53)		No bacterial infection (n = 39)		OR	P	AdjP	Log2FC	P	AdjP
	n	%	n	%						
Alistipes	22	41.5%	8	20.5%	2.72	0.043	0.543	1.741	0.051	0.641
Bacteroides	27	50.9%	22	56.4%	0.804	0.675	1.000	-0.520	0.381	0.975
Bifidobacterium	4	7.5%	6	15.4%	0.453	0.314	1.000	1.679	0.245	0.975
Blautia_A	5	9.4%	2	5.1%	1.914	0.695	1.000	3.400	0.419	0.975
Chlamydia	12	22.6%	6	15.4%	1.602	0.436	1.000	-1.675	0.462	0.975
Dysosmobacter	5	9.4%	3	7.7%	1.247	1.000	1.000	0.222	0.771	0.975
Enterocloster	10	18.9%	5	12.8%	1.574	0.571	1.000	0.433	0.404	0.975
Enterococcus_B	15	28.3%	1	2.6%	14.677	0.001	**0.037**	10.431	0.001	**0.030**
Escherichia	11	20.8%	9	23.1%	0.874	0.803	1.000	-3.859	0.554	0.975
Faecalibacterium	11	20.8%	9	23.1%	0.874	0.803	1.000	0.835	0.835	0.975
Flavonifractor	8	15.1%	4	10.3%	1.548	0.549	1.000	1.276	0.478	0.975
Fusicatenibacter	3	5.7%	2	5.1%	1.109	1.000	1.000	-0.648	0.936	0.975
Gemmiger	3	5.7%	3	7.7%	0.723	0.696	1.000	-3.579	0.658	0.975
Klebsiella	6	11.3%	2	5.1%	2.341	0.459	1.000	1.671	0.312	0.975
Lactococcus	20	37.7%	14	35.9%	1.081	1.000	1.000	-2.218	0.975	0.975
Lactococcus_A	4	7.5%	3	7.7%	0.98	1.000	1.000	-1.829	0.959	0.975
Lawsonibacter	3	5.7%	3	7.7%	0.723	0.696	1.000	3.484	0.685	0.975
Leuconostoc	8	15.1%	7	17.9%	0.815	0.779	1.000	0.462	0.778	0.975
Mediterraneibacter	5	9.4%	3	7.7%	1.247	1.000	1.000	-0.516	0.808	0.975
Odoribacter	8	15.1%	4	10.3%	1.548	0.549	1.000	1.566	0.548	0.975
Parabacteroides	16	30.2%	13	33.3%	0.866	0.822	1.000	0.947	0.931	0.975
Prevotella	6	11.3%	8	20.5%	0.499	0.253	1.000	-4.159	0.242	0.975
Rothia	4	7.5%	1	2.6%	3.069	0.391	1.000	7.815	0.286	0.975
Streptococcus	7	13.2%	5	12.8%	1.034	1.000	1.000	-1.304	0.957	0.975
Veillonella	6	11.3%	4	10.3%	1.116	1.000	1.000	4.423	0.776	0.975

Table S5: Phage-host groups in patients with a bacterial infection

Length	Estimated completeness	Genus	Family	Host prediction method (exc. Prophage)	Prophage species hits	BACPHLIP temperate	Lifestyle
25 kb	64%	Genus_65	Family_2	RaFAH + PHIST	E. faecium (n = 8) & E. hirae (n = 1)		Temperate
32 kb	82%	Genus_65	Family_2	RaFAH + PHIST	E. faecium (n = 6) & E. hirae (n = 1)		Temperate
33 kb	83%	Genus_65	Family_2	RaFAH + PHIST	E. faecium (n = 6) & E. hirae (n = 1)		Temperate
26 kb	66%	Genus_65	Family_2	RaFAH + PHIST	E. faecium (n = 6) & E. lactis (n = 1)		Temperate
26 kb	66%	Genus_65	Family_2	RaFAH + PHIST + tRNA	E. faecium (n = 8) & E. hirae (n = 1)	Yes	Temperate
34 kb	85%	Genus_65	Family_2	RaFAH + PHIST + tRNA	E. faecium (n = 7) & E. hirae (n = 1)	Yes	Temperate
27 kb	69%	Genus_65	Family_2	RaFAH + PHIST	E. faecium (n = 5) & E. lactis (n = 1)		Temperate
32 kb	83%	Genus_67	Family_2	RaFAH + PHIST	E. faecium (n = 16)		Temperate
72 kb	100%	Genus_67	Family_2	RaFAH + PHIST + tRNA	E. faecium (n = 13)	Yes	Temperate
19 kb	50%	Genus_67	Family_2	RaFAH + PHIST	E. faecium (n = 18)		Temperate
32 kb	83%	Genus_67	Family_2	RaFAH + PHIST	E. faecium (n = 17)	Yes	Temperate
42 kb	100%	Genus_67	Family_2	RaFAH + PHIST + tRNA	E. faecium (n = 14)	Yes	Temperate
38 kb	99%	Genus_67	Family_2	RaFAH + PHIST + tRNA	E. faecium (n = 14)	Yes	Temperate
19 kb	100%	Genus_35	Family_23	RaFAH			Virulent
22 kb	54%	Genus_120	Family_33	RaFAH + PHIST	E. durans (n = 1)	Yes	Temperate
26 kb	62%	Genus_27	Family_33	RaFAH + PHIST			Virulent
26 kb	60%	Genus_120	Family_42	RaFAH + PHIST	E. faecium (n = 2) & E. hirae (n = 1)		Temperate
21 kb	50%	Genus_273	Family_61	RaFAH + PHIST			Virulent
* Species level host prediction only possible if prophage hit to a high-quality bacterial MAG.							

Table S6: Lifestyle prediction of *Enterococcus B* phages

Host_Genus	Prevalence				Fisher test for equal proportions			Wilcoxon rank-sum test		
	Gastrointestinal bleeding (n = 9)		No gastrointestinal bleeding (n = 59)		OR	P	AdjP	Log2FC	P	AdjP
	n	%	n	%						
Alistipes	2	22.2%	20	33.9%	0.562	0.707	1.000	-3.834	0.452	0.603
Bacteroides	2	22.2%	33	55.9%	0.230	0.079	0.545	-2.081	0.084	0.501
Bifidobacterium	0	0.0%	6	10.2%	0.000	1.000	1.000	-Inf	0.330	0.535
Blautia_A	2	22.2%	5	8.5%	3.017	0.230	0.907	-3.019	0.257	0.535
Chlamydia	3	33.3%	14	23.7%	1.595	0.680	1.000	2.719	0.552	0.664
Dysosmobacter	0	0.0%	7	11.9%	0.000	0.582	1.000	-Inf	0.287	0.535
Enterocloster	2	22.2%	7	11.9%	2.094	0.340	0.907	1.470	0.357	0.535
Enterococcus_B	4	44.4%	9	15.3%	4.315	0.060	0.545	3.156	0.029	0.487
Escherichia	4	44.4%	14	23.7%	2.530	0.231	0.907	-0.575	0.278	0.535
Faecalibacterium	1	11.1%	16	27.1%	0.340	0.432	1.000	-8.892	0.234	0.535
Flavonifractor	1	11.1%	6	10.2%	1.103	1.000	1.000	-0.339	0.918	0.945
Fusicatenibacter	0	0.0%	5	8.5%	0.000	1.000	1.000	-Inf	0.379	0.535
Gemmiger	0	0.0%	5	8.5%	0.000	1.000	1.000	-Inf	0.379	0.535
Klebsiella	3	33.3%	6	10.2%	4.280	0.091	0.545	1.982	0.061	0.487
Lactococcus	2	22.2%	18	30.5%	0.655	1.000	1.000	-2.024	0.629	0.689
Lawsonibacter	0	0.0%	5	8.5%	0.000	1.000	1.000	-Inf	0.379	0.535
Leuconostoc	1	11.1%	6	10.2%	1.103	1.000	1.000	-0.699	0.945	0.945
Mediterraneibacter	0	0.0%	6	10.2%	0.000	1.000	1.000	-Inf	0.330	0.535
Odoribacter	0	0.0%	9	15.3%	0.000	0.595	1.000	-Inf	0.219	0.535
Parabacteroides	0	0.0%	19	32.2%	0.000	0.053	0.545	-Inf	0.052	0.487
Prevotella	0	0.0%	10	16.9%	0.000	0.336	0.907	-Inf	0.191	0.535
Ruminococcus_B	1	11.1%	4	6.8%	1.703	0.520	1.000	0.806	0.631	0.689
Streptococcus	2	22.2%	9	15.3%	1.575	0.631	1.000	0.945	0.553	0.664
Veillonella	2	22.2%	6	10.2%	2.480	0.285	0.907	2.633	0.238	0.535

Table S7: Association of phage-host groups with gastrointestinal bleeding

Host_Genus	Prevalence				Fisher test for equal proportions			Wilcoxon rank-sum test		
	Hepatic encephalopathy (n = 25)		No hepatic encephalopathy (n = 66)		OR	P	AdjP	Log2FC	P	AdjP
	n	%	n	%						
Alistipes	10	40.0%	20	30.3%	1.526	0.456	1.000	0.082	0.358	0.825
Bacteroides	15	60.0%	34	51.5%	1.406	0.491	1.000	-0.573	0.650	0.825
Bifidobacterium	2	8.0%	8	12.1%	0.633	0.721	1.000	2.480	0.676	0.825
Blautia_A	1	4.0%	6	9.1%	0.420	0.669	1.000	-7.116	0.403	0.825
Chlamydia	6	24.0%	15	22.7%	1.073	1.000	1.000	-2.678	1.000	1.000
Dysosmobacter	1	4.0%	8	12.1%	0.305	0.435	1.000	-5.880	0.226	0.825
Enterocloster	4	16.0%	9	13.6%	1.204	0.747	1.000	1.140	0.726	0.825
Enterococcus_B	1	4.0%	12	18.2%	0.190	0.104	1.000	-7.316	0.081	0.825
Escherichia	7	28.0%	14	21.2%	1.438	0.579	1.000	-0.817	0.643	0.825
Faecalibacterium	4	16.0%	18	27.3%	0.511	0.411	1.000	-0.575	0.295	0.825
Flavonifractor	4	16.0%	7	10.6%	1.596	0.486	1.000	-0.290	0.490	0.825
Fusicatenibacter	0	0.0%	5	7.6%	0.000	0.317	1.000	-Inf	0.163	0.825
Gemmiger	1	4.0%	5	7.6%	0.512	1.000	1.000	-5.288	0.515	0.825
Klebsiella	1	4.0%	8	12.1%	0.305	0.435	1.000	-1.087	0.276	0.825
Lactococcus	9	36.0%	20	30.3%	1.290	0.622	1.000	1.366	0.375	0.825
Lawsonibacter	1	4.0%	5	7.6%	0.512	1.000	1.000	5.255	0.584	0.825
Leuconostoc	3	12.0%	9	13.6%	0.865	1.000	1.000	1.698	0.874	0.947
Mediterraneibacter	1	4.0%	7	10.6%	0.354	0.437	1.000	-4.020	0.324	0.825
Odoribacter	3	12.0%	10	15.2%	0.766	1.000	1.000	-3.754	0.699	0.825
Parabacteroides	7	28.0%	21	31.8%	0.835	0.803	1.000	0.701	0.909	0.947
Prevotella	4	16.0%	12	18.2%	0.859	1.000	1.000	-5.041	0.693	0.825
Rothia	3	12.0%	2	3.0%	4.279	0.125	1.000	5.837	0.093	0.825
Ruminococcus_B	1	4.0%	5	7.6%	0.512	1.000	1.000	-0.555	0.570	0.825
Streptococcus	3	12.0%	11	16.7%	0.685	0.750	1.000	-2.013	0.566	0.825
Veillonella	2	8.0%	9	13.6%	0.554	0.721	1.000	-0.735	0.520	0.825

Table S8: Association of phage-host groups with hepatic encephalopathy

Host_Genus	Prevalence				Fisher test for equal proportions			Wilcoxon rank-sum test		
	Alcoholic hepatitis (n = 33)		No alcoholic hepatitis (n = 59)		OR	P	AdjP	Log2FC	P	AdjP
	n	%	n	%						
Alistipes	9	27.3%	21	35.6%	0.681	0.491	0.876	-1.287	0.494	0.882
Bacteroides	20	60.6%	29	49.2%	1.583	0.384	0.863	-0.617	0.313	0.711
Bifidobacterium	6	18.2%	5	8.5%	2.376	0.193	0.863	-1.657	0.199	0.711
Blautia_A	3	9.1%	4	6.8%	1.370	0.698	1.000	1.338	0.684	0.911
Chlamydia	3	9.1%	18	30.5%	0.231	0.021	0.522	-6.854	0.014	0.349
Dysosmobacter	3	9.1%	4	6.8%	1.370	0.698	1.000	-3.186	0.777	0.911
Enterocloster	5	15.2%	8	13.6%	1.137	1.000	1.000	0.058	0.925	0.925
Enterococcus_B	8	24.2%	6	10.2%	2.792	0.127	0.863	5.702	0.053	0.663
Escherichia	8	24.2%	14	23.7%	1.028	1.000	1.000	1.925	0.773	0.911
Faecalibacterium	7	21.2%	13	22.0%	0.953	1.000	1.000	-1.368	0.752	0.911
Flavonifractor	4	12.1%	7	11.9%	1.024	1.000	1.000	2.472	0.874	0.911
Fusicatenibacter	2	6.1%	3	5.1%	1.202	1.000	1.000	0.394	0.836	0.911
Gemmiger	3	9.1%	2	3.4%	2.815	0.346	0.863	0.598	0.263	0.711
Klebsiella	3	9.1%	7	11.9%	0.745	1.000	1.000	-0.528	0.718	0.911
Lactococcus	12	36.4%	15	25.4%	1.667	0.341	0.863	-0.789	0.466	0.882
Lactococcus_A	2	6.1%	3	5.1%	1.202	1.000	1.000	-0.857	0.836	0.911
Lawsonibacter	0	0.0%	5	8.5%	0.000	0.156	0.863	-Inf	0.089	0.711
Leuconostoc	7	21.2%	5	8.5%	2.871	0.109	0.863	-2.248	0.123	0.711
Mediterraneibacter	1	3.0%	7	11.9%	0.235	0.251	0.863	-1.894	0.157	0.711
Odoribacter	7	21.2%	7	11.9%	1.984	0.243	0.863	3.213	0.216	0.711
Parabacteroides	12	36.4%	17	28.8%	1.406	0.489	0.876	-3.060	0.874	0.911
Prevotella	6	18.2%	9	15.3%	1.232	0.772	1.000	-2.100	0.800	0.911
Ruminococcus_B	1	3.0%	5	8.5%	0.341	0.414	0.863	-9.180	0.296	0.711
Streptococcus	6	18.2%	7	11.9%	1.641	0.534	0.890	2.844	0.444	0.882
Veillonella	2	6.1%	8	13.6%	0.415	0.321	0.863	-3.328	0.249	0.711

Table S9: Association of phage-host groups with alcoholic hepatitis

**Multivariable logistic regression - Bacterial infection in TIPS cohort**					
Coefficients:					
	Estimate	Std. Error	z value	Pr(>|z|)	
(Intercept)	1.737737	1.915298	0.907	0.364	
EntB_TIPS$Abundance	-15.94348	7.801731	-2.044	0.041	*
EntB_TIPS$CLIFCAD	0.006111	0.040486	0.151	0.88	
EntB_TIPS$AB2	-0.592219	0.676295	-0.876	0.381	
EntB_TIPS$Indication1	1.510591	1.114812	1.355	0.175	
Significance code: < 0.05 '*'					
(Dispersion parameter for binomial family taken to be 1)					
Null deviance: 75.694 on 83 degrees of freedom					
Residual deviance: 59.010 on 79 degrees of freedom					
(10 observations deleted due to missingness)					
AIC: 69.01					
Number of Fisher Scoring iterations: 6					

Table S10: Multivariable logistic regression for presence of bacterial infections in the validation cohort.

Length	Estimated completeness	Genus	Family	Host prediction method (exc. Prophage)	Prophage species hits	BACPHLIP temperate	Lifestyle
41 kb	99%	Genus_1082	Family_11	RaFAH + PHIST		Yes	Temperate
29 kb	76%	Genus_1213	Family_11	RaFAH + PHIST + tRNA	E. faecium (n = 2)	Yes	Temperate
36 kb	95%	Genus_1964	Family_11	RaFAH + PHIST		Yes	Temperate
43 kb	100%	Genus_317	Family_11	RaFAH + PHIST + tRNA	E. faecium (n = 1) & E. lactis (n = 1)	Yes	Temperate
48 kb	100%	Genus_317	Family_11	RaFAH + PHIST + tRNA	E. faecium (n = 1) & E. lactis (n = 1)	Yes	Temperate
23 kb	62%	Genus_317	Family_11	RaFAH + PHIST	E. faecium (n = 1)	Yes	Temperate
39 kb	100%	Genus_317	Family_11	RaFAH + PHIST + tRNA		Yes	Temperate
37 kb	100%	Genus_317	Family_11	RaFAH + PHIST + tRNA	E. faecium (n = 1) & E. hirae (n = 1)	Yes	Temperate
28 kb	76%	Genus_317	Family_11	RaFAH + PHIST + tRNA		Yes	Temperate
40 kb	100%	Genus_317	Family_11	RaFAH + PHIST	E. faecium (n = 1) & E. lactis (n = 1)	Yes	Temperate
33 kb	83%	Genus_317	Family_11	RaFAH + PHIST	E. faecium (n = 1) & E. lactis (n = 1)		Temperate
33 kb	84%	Genus_317	Family_11	RaFAH + PHIST + tRNA	E. faecium (n = 2) & E. hirae (n = 1)	Yes	Temperate
28 kb	70%	Genus_317	Family_11	RaFAH + PHIST	E. faecium (n = 1) & E. lactis (n = 2)		Temperate
45 kb	100%	Genus_317	Family_11	RaFAH + PHIST + tRNA	E. hirae (n = 1)	Yes	Temperate
26 kb	60%	Genus_1979	Family_11	RaFAH + PHIST			Virulent
22 kb	54%	Efquatrovirus	Family_127	RaFAH + PHIST			Virulent
22 kb	52%	Genus_899	Family_201	RaFAH + PHIST + tRNA	E. faecium (n = 3) & E. lactis (n = 1)		Temperate
* Species level host prediction only possible if prophage hit to a high-quality bacterial MAG.							

Table S11: Temperateness of *Enterococcus B* phages in the validation cohort

BioProject accession	BioSample accession	Run accession
ERP172927	ERS25304041	ERR15372593
ERP172927	ERS25304042	ERR15372805
ERP172927	ERS25304043	ERR15372796
ERP172927	ERS25304044	ERR15372718
ERP172927	ERS25304045	ERR15372822
ERP172927	ERS25304046	ERR15372740
ERP172927	ERS25304047	ERR15372727
ERP172927	ERS25304048	ERR15372838
ERP172927	ERS25304049	ERR15372751
ERP172927	ERS25304050	ERR15372630
ERP172927	ERS25304051	ERR15372588
ERP172927	ERS25304052	ERR15372799
ERP172927	ERS25304053	ERR15372716
ERP172927	ERS25304054	ERR15372812
ERP172927	ERS25304055	ERR15372738
ERP172927	ERS25304056	ERR15372754
ERP172927	ERS25304057	ERR15372674
ERP172927	ERS25304058	ERR15372652
ERP172927	ERS25304059	ERR15372861
ERP172927	ERS25304060	ERR15372743
ERP172927	ERS25304061	ERR15372801
ERP172927	ERS25304062	ERR15372569
ERP172927	ERS25304063	ERR15372678
ERP172927	ERS25304064	ERR15372810
ERP172927	ERS25304065	ERR15372641
ERP172927	ERS25304066	ERR15372722
ERP172927	ERS25304067	ERR15372821
ERP172927	ERS25304068	ERR15372715
ERP172927	ERS25304069	ERR15372850

ERP172927	ERS25304070	ERR15372612
ERP172927	ERS25304071	ERR15372840
ERP172927	ERS25304072	ERR15372755
ERP172927	ERS25304073	ERR15372582
ERP172927	ERS25304074	ERR15372638
ERP172927	ERS25304075	ERR15372785
ERP172927	ERS25304076	ERR15372592
ERP172927	ERS25304077	ERR15372845
ERP172927	ERS25304078	ERR15372833
ERP172927	ERS25304079	ERR15372573
ERP172927	ERS25304080	ERR15372865
ERP172927	ERS25304081	ERR15372711
ERP172927	ERS25304082	ERR15372784
ERP172927	ERS25304083	ERR15372645
ERP172927	ERS25304084	ERR15372752
ERP172927	ERS25304085	ERR15372766
ERP172927	ERS25304086	ERR15372618
ERP172927	ERS25304087	ERR15372848
ERP172927	ERS25304088	ERR15372611
ERP172927	ERS25304089	ERR15372579
ERP172927	ERS25304090	ERR15372675
ERP172927	ERS25304092	ERR15372683
ERP172927	ERS25304093	ERR15372607
ERP172927	ERS25304094	ERR15372844
ERP172927	ERS25304095	ERR15372590
ERP172927	ERS25304096	ERR15372596
ERP172927	ERS25304097	ERR15372729
ERP172927	ERS25304098	ERR15372717
ERP172927	ERS25304099	ERR15372773
ERP172927	ERS25304100	ERR15372719
ERP172927	ERS25304101	ERR15372581
ERP172927	ERS25304102	ERR15372837
ERP172927	ERS25304103	ERR15372636
ERP172927	ERS25304104	ERR15372650
ERP172927	ERS25304105	ERR15372673
ERP172927	ERS25304106	ERR15372774
ERP172927	ERS25304107	ERR15372798
ERP172927	ERS25304108	ERR15372586
ERP172927	ERS25304109	ERR15372699
ERP172927	ERS25304110	ERR15372760
ERP172927	ERS25304111	ERR15372742
ERP172927	ERS25304112	ERR15372648
ERP172927	ERS25304113	ERR15372666
ERP172927	ERS25304114	ERR15372866

ERP172927	ERS25304115	ERR15372658
ERP172927	ERS25304116	ERR15372827
ERP172927	ERS25304117	ERR15372599
ERP172927	ERS25304118	ERR15372747
ERP172927	ERS25304119	ERR15372728
ERP172927	ERS25304120	ERR15372712
ERP172927	ERS25304121	ERR15372655
ERP172927	ERS25304122	ERR15372775
ERP172927	ERS25304123	ERR15372709
ERP172927	ERS25304124	ERR15372707
ERP172927	ERS25304125	ERR15372772
ERP172927	ERS25304126	ERR15372576
ERP172927	ERS25304127	ERR15372736
ERP172927	ERS25304128	ERR15372620
ERP172927	ERS25304129	ERR15372705
ERP172927	ERS25304130	ERR15372831
ERP172927	ERS25304131	ERR15372830
ERP172927	ERS25304132	ERR15372813
ERP172927	ERS25304133	ERR15372696
ERP172927	ERS25304134	ERR15372841
ERP172927	ERS25304135	ERR15372878
ERP172927	ERS25304136	ERR15372687
ERP172927	ERS25304137	ERR15372698
ERP172927	ERS25304138	ERR15372642
ERP172927	ERS25304139	ERR15372803
ERP172927	ERS25304140	ERR15372748
ERP172927	ERS25304141	ERR15372744
ERP172927	ERS25304142	ERR15372859
ERP172927	ERS25304143	ERR15372594
ERP172927	ERS25304144	ERR15372568
ERP172927	ERS25304145	ERR15372703
ERP172927	ERS25304146	ERR15372591
ERP172927	ERS25304147	ERR15372860
ERP172927	ERS25304149	ERR15372834
ERP172927	ERS25304150	ERR15372587
ERP172927	ERS25304151	ERR15372794
ERP172927	ERS25304152	ERR15372706
ERP172927	ERS25304153	ERR15372677
ERP172927	ERS25304154	ERR15372807
ERP172927	ERS25304155	ERR15372790
ERP172927	ERS25304156	ERR15372828
ERP172927	ERS25304157	ERR15372753
ERP172927	ERS25304158	ERR15372676
ERP172927	ERS25304159	ERR15372634

ERP172927	ERS25304160	ERR15372873
ERP172927	ERS25304161	ERR15372768
ERP172927	ERS25304162	ERR15372809
ERP172927	ERS25304163	ERR15372704
ERP172927	ERS25304164	ERR15372782
ERP172927	ERS25304165	ERR15372572
ERP172927	ERS25304166	ERR15372691
ERP172927	ERS25304167	ERR15372720
ERP172927	ERS25304168	ERR15372580
ERP172927	ERS25304169	ERR15372605
ERP172927	ERS25304170	ERR15372758
ERP172927	ERS25304171	ERR15372684
ERP172927	ERS25304172	ERR15372695
ERP172927	ERS25304173	ERR15372786
ERP172927	ERS25304174	ERR15372872
ERP172927	ERS25304175	ERR15372874
ERP172927	ERS25304176	ERR15372765
ERP172927	ERS25304177	ERR15372868
ERP172927	ERS25304178	ERR15372797
ERP172927	ERS25304179	ERR15372616
ERP172927	ERS25304180	ERR15372791
ERP172927	ERS25304181	ERR15372778
ERP172927	ERS25304182	ERR15372700
ERP172927	ERS25304183	ERR15372864
ERP172927	ERS25304184	ERR15372849
ERP172927	ERS25304185	ERR15372629
ERP172927	ERS25304186	ERR15372854
ERP172927	ERS25304187	ERR15372858
ERP172927	ERS25304189	ERR15372603
ERP172927	ERS25304190	ERR15372852
ERP172927	ERS25304191	ERR15372816
ERP172927	ERS25304192	ERR15372686
ERP172927	ERS25304193	ERR15372633
ERP172927	ERS25304194	ERR15372584
ERP172927	ERS25304195	ERR15372857
ERP172927	ERS25304196	ERR15372731
ERP172927	ERS25304197	ERR15372746
ERP172927	ERS25304198	ERR15372876
ERP172927	ERS25304199	ERR15372606
ERP172927	ERS25304200	ERR15372661
ERP172927	ERS25304201	ERR15372811
ERP172927	ERS25304202	ERR15372776
ERP172927	ERS25304203	ERR15372820
ERP172927	ERS25304204	ERR15372628

ERP172927	ERS25304205	ERR15372670
ERP172927	ERS25304206	ERR15372614
ERP172927	ERS25304207	ERR15372631
ERP172927	ERS25304208	ERR15372624
ERP172927	ERS25304209	ERR15372862
ERP172927	ERS25304210	ERR15372763
ERP172927	ERS25304211	ERR15372867
ERP172927	ERS25304212	ERR15372585
ERP172927	ERS25304213	ERR15372804
ERP172927	ERS25304214	ERR15372646
ERP172927	ERS25304215	ERR15372602
ERP172927	ERS25304216	ERR15372832
ERP172927	ERS25304217	ERR15372597
ERP172927	ERS25304218	ERR15372571
ERP172927	ERS25304219	ERR15372623
ERP172927	ERS25304220	ERR15372737
ERP172927	ERS25304221	ERR15372713
ERP172927	ERS25304222	ERR15372745
ERP172927	ERS25304223	ERR15372800
ERP172927	ERS25304225	ERR15372808
ERP172927	ERS25304226	ERR15372575
ERP172927	ERS25304227	ERR15372651
ERP172927	ERS25304228	ERR15372749
ERP172927	ERS25304229	ERR15372643
ERP172927	ERS25304230	ERR15372759
ERP172927	ERS25304231	ERR15372610
ERP172927	ERS25304232	ERR15372788
ERP172927	ERS25304233	ERR15372644
ERP172927	ERS25304234	ERR15372793
ERP172927	ERS25304235	ERR15372725
ERP172927	ERS25304236	ERR15372853
ERP172927	ERS25304237	ERR15372781
ERP172927	ERS25304238	ERR15372870
ERP172927	ERS25304239	ERR15372787
ERP172927	ERS25304240	ERR15372679
ERP172927	ERS25304242	ERR15372780
ERP172927	ERS25304243	ERR15372627
ERP172927	ERS25304244	ERR15372653
ERP172927	ERS25304245	ERR15372697
ERP172927	ERS25304246	ERR15372665
ERP172927	ERS25304247	ERR15372847
ERP172927	ERS25304248	ERR15372789
ERP172927	ERS25304249	ERR15372734
ERP172927	ERS25304250	ERR15372672

ERP172927	ERS25304251	ERR15372732
ERP172927	ERS25304252	ERR15372735
ERP172927	ERS25304253	ERR15372649
ERP172927	ERS25304254	ERR15372682
ERP172927	ERS25304255	ERR15372818
ERP172927	ERS25304256	ERR15372771
ERP172927	ERS25304257	ERR15372777
ERP172927	ERS25304258	ERR15372730
ERP172927	ERS25304259	ERR15372680
ERP172927	ERS25304260	ERR15372656
ERP172927	ERS25304261	ERR15372842
ERP172927	ERS25304262	ERR15372823
ERP172927	ERS25304263	ERR15372640
ERP172927	ERS25304264	ERR15372815
ERP172927	ERS25304265	ERR15372615
ERP172927	ERS25304266	ERR15372669
ERP172927	ERS25304267	ERR15372681
ERP172927	ERS25304268	ERR15372843
ERP172927	ERS25304269	ERR15372604
ERP172927	ERS25304270	ERR15372617
ERP172927	ERS25304271	ERR15372608
ERP172927	ERS25304272	ERR15372625
ERP172927	ERS25304273	ERR15372689
ERP172927	ERS25304274	ERR15372702
ERP172927	ERS25304275	ERR15372622
ERP172927	ERS25304276	ERR15372814
ERP172927	ERS25304277	ERR15372741
ERP172927	ERS25304278	ERR15372802
ERP172927	ERS25304279	ERR15372871
ERP172927	ERS25304280	ERR15372708
ERP172927	ERS25304281	ERR15372574
ERP172927	ERS25304282	ERR15372756
ERP172927	ERS25304283	ERR15372632
ERP172927	ERS25304284	ERR15372877
ERP172927	ERS25304285	ERR15372726
ERP172927	ERS25304286	ERR15372839
ERP172927	ERS25304287	ERR15372764
ERP172927	ERS25304288	ERR15372767
ERP172927	ERS25304289	ERR15372733
ERP172927	ERS25304290	ERR15372663
ERP172927	ERS25304291	ERR15372826
ERP172927	ERS25304292	ERR15372693
ERP172927	ERS25304293	ERR15372829
ERP172927	ERS25304294	ERR15372577

ERP172927	ERS25304295	ERR15372701
ERP172927	ERS25304296	ERR15372750
ERP172927	ERS25304298	ERR15372770
ERP172927	ERS25304299	ERR15372783
ERP172927	ERS25304300	ERR15372667
ERP172927	ERS25304301	ERR15372819
ERP172927	ERS25304302	ERR15372757
ERP172927	ERS25304303	ERR15372710
ERP172927	ERS25304304	ERR15372637
ERP172927	ERS25304305	ERR15372792
ERP172927	ERS25304306	ERR15372635
ERP172927	ERS25304307	ERR15372578
ERP172927	ERS25304308	ERR15372664
ERP172927	ERS25304310	ERR15372654
ERP172927	ERS25304311	ERR15372668
ERP172927	ERS25304312	ERR15372671
ERP172927	ERS25304313	ERR15372856
ERP172927	ERS25304314	ERR15372639
ERP172927	ERS25304315	ERR15372685
ERP172927	ERS25304316	ERR15372863
ERP172927	ERS25304317	ERR15372595
ERP172927	ERS25304318	ERR15372613
ERP172927	ERS25304320	ERR15372721
ERP172927	ERS25304321	ERR15372657
ERP172927	ERS25304322	ERR15372659
ERP172927	ERS25304323	ERR15372851
ERP172927	ERS25304324	ERR15372855
ERP172927	ERS25304325	ERR15372714
ERP172927	ERS25304326	ERR15372609
ERP172927	ERS25304327	ERR15372589
ERP172927	ERS25304328	ERR15372601
ERP172927	ERS25304329	ERR15372660
ERP172927	ERS25304330	ERR15372836
ERP172927	ERS25304331	ERR15372662
ERP172927	ERS25304332	ERR15372806
ERP172927	ERS25304333	ERR15372583
ERP172927	ERS25304334	ERR15372692
ERP172927	ERS25304335	ERR15372739
ERP172927	ERS25304336	ERR15372835
ERP172927	ERS25304337	ERR15372621
ERP172927	ERS25304338	ERR15372817
ERP172927	ERS25304339	ERR15372869
ERP172927	ERS25304340	ERR15372875
ERP172927	ERS25304341	ERR15372769

ERP172927	ERS25304342	ERR15372761
ERP172927	ERS25304343	ERR15372688
ERP172927	ERS25304344	ERR15372694
ERP172927	ERS25304345	ERR15372762
ERP172927	ERS25304346	ERR15372570
ERP172927	ERS25304347	ERR15372647
ERP172927	ERS25304348	ERR15372619
ERP172927	ERS25304349	ERR15372795

Table S12: Sequencing data are publicly available at: https://www.ebi.ac.uk/ena . The ENA accession numbers are provided in this table. ENA – European Nucleotide Archive.

Supplementary References

[1] **Conceição-Neto, N, Zeller, M**, Lefrère, H, et al. Modular approach to customise sample preparation procedures for viral metagenomics: a reproducible protocol for virome analysis. *Nat. Publ. Group*. 2015

[2] Bolger, AM, Lohse, M, and Usadel, B. Trimmomatic: A flexible trimmer for Illumina sequence data. *Bioinformatics*. 2014;30. 2114–2120

[3] Bankevich, A, Nurk, S, Antipov, D, et al. SPAdes: A New Genome Assembly Algorithm and Its Applications to Single-Cell Sequencing. *J. Comput. Biol.* 2012;19. 455–477

[4] Shen, W, Le, S, Li, Y, et al. SeqKit: A cross-platform and ultrafast toolkit for FASTA/Q file manipulation. *PLoS ONE*. 2016;11. 1–10

[5] Camacho, C, Coulouris, G, Avagyan, V, et al. BLAST+: architecture and applications. *BMC Bioinformatics*. 2009;10. 421

[6] **Nayfach, S, Camargo, AP**, Schulz, F, et al. CheckV assesses the quality and completeness of metagenome-assembled viral genomes. *Nat. Biotechnol.* 2020. 1–8

[7] Wood, DE, Lu, J, and Langmead, B. Improved metagenomic analysis with Kraken 2. *Genome Biol.* 2019;20. 1–13

[8] Buchfink, B, Xie, C, and Huson, DH. Fast and sensitive protein alignment using DIAMOND. *Nat. Methods*. 2014;12. 59–60

[9] Ondov, BD, Bergman, NH, and Phillippy, AM. Interactive metagenomic visualization in a Web browser. *BMC Bioinformatics*. 2011;12. 385

[10] **Guo, J, Bolduc, B**, Zayed, AA, et al. VirSorter2: a multi-classifier, expert-guided approach to detect diverse DNA and RNA viruses. *Microbiome*. 2021. 1–13

[11] Li, H, and Durbin, R. Fast and accurate short read alignment with Burrows-Wheeler transform. *Bioinformatics*. 2009;25. 1754–1760

[12] Li, H, Handsaker, B, Wysoker, A, et al. The Sequence Alignment/Map format and SAMtools. *Bioinformatics*. 2009;25. 2078–2079

[13] Davis, NM, Proctor, DM, Holmes, SP, et al. Simple statistical identification and removal of contaminant sequences in marker-gene and metagenomics data. *Microbiome*. 2018;6. 226

[14] McMurdie, PJ, and Holmes, S. phyloseq: An R package for reproducible interactive analysis and graphics of microbiome census data. *PLoS ONE*. 2013;8. e61217

[15] Nayfach, S, Paez-Espino, D, Strozzi, F, et al. Metegenomic Compendium of 189,680 DNA Viruses From the Human Gut Microbiome. *Nat. Biotechnol.* 2021;39

[16] Hyatt, D, Chen, G-L, LoCascio, PF, et al. Prodigal: prokaryotic gene recognition and translation initiation site identification. *BMC Bioinformatics*. 2010;11. 119

[17] Haft, DH, DiCuccio, M, Badretdin, A, et al. RefSeq: An update on prokaryotic genome annotation and curation. *Nucleic Acids Res.* 2018;46. D851–D860

[18] Coelho, LP, Alves, R, Monteiro, P, et al. NG-meta-profiler: fast processing of metagenomes using NGLess, a domain-specific language. *Microbiome*. 2019;7. 84

[19] Li, D, Liu, C-M, Luo, R, et al. MEGAHIT: an ultra-fast single-node solution for large and complex metagenomics assembly via succinct de Bruijn graph. *Bioinformatics*. 2015;31. 1674–1676

[20] Kang, DD, Li, F, Kirton, E, et al. MetaBAT 2: an adaptive binning algorithm for robust and efficient genome reconstruction from metagenome assemblies. *PeerJ*. 2019;7. e7359

[21] **Almeida, A, Nayfach, S**, Boland, M, et al. A unified catalog of 204,938 reference genomes from the human gut microbiome. *Nat. Biotechnol.*

[22] Chaumeil, P-A, Mussig, AJ, Hugenholtz, P, et al. GTDB-Tk: a toolkit to classify genomes with the Genome Taxonomy Database. *Bioinformatics*. 2019. btz848

[23] Zielezinski, A, Deorowicz, S, and Gudyś, A. PHIST: fast and accurate prediction of prokaryotic hosts from metagenomic viral sequences. *Bioinformatics*. 2022;38. 1447–1449

[24] Coutinho, FH, Zaragoza-Solas, A, López-Pérez, M, et al. RaFAH: Host prediction for viruses of Bacteria and Archaea based on protein content. *Patterns*. 2021;2. 100274

[25] **Gregory, AC, Zablocki, O**, Zayed, AA, et al. The Gut Virome Database Reveals Age-Dependent Patterns of Virome Diversity in the Human Gut. *Cell Host Microbe*. 2020. 1–17

[26] Bland, C, Ramsey, TL, Sabree, F, et al. CRISPR Recognition Tool (CRT): a tool for automatic detection of clustered regularly interspaced palindromic repeats. *BMC Bioinformatics*. 2007;8. 209

[27] Laslett, D, and Canback, B. ARAGORN, a program to detect tRNA genes and tmRNA genes in nucleotide sequences. *Nucleic Acids Res.* 2004;32. 11–16

[28] Hockenberry, AJ, and Wilke, CO. BACPHLIP: predicting bacteriophage lifestyle from conserved protein domains. *PeerJ*. 2021;9. e11396

[29] Ruscheweyh, H-J, Milanese, A, Paoli, L, et al. mOTUs: Profiling Taxonomic Composition, Transcriptional Activity and Strain Populations of Microbial Communities. *Curr. Protoc.* 2021;1. e218

[30] Mende, DR, Letunic, I, Maistrenko, OM, et al. proGenomes2: an improved database for accurate and consistent habitat, taxonomic and functional annotations of prokaryotic genomes. *Nucleic Acids Res.* 2019;48. D621–D625
