# Supplementary material for: Lactococcus A phages predict ACLF while Enterococcus B phages predict bacterial infection in decompensated cirrhosis
Source: JHEP Rep. 2025 Oct 10;8(1):101622. doi: 10.1016/j.jhepr.2025.101622 (PMC12721046; doi:10.1016/j.jhepr.2025.101622)
Supplement: Multimedia component 2 [file mmc2.docx]

**JHEP Reports**

**CTAT methods**

Tables for a “Complete, Transparent, Accurate and Timely account” (CTAT) are now mandatory for all revised submissions. The aim is to enhance the reproducibility of methods.

- Only include the parts relevant to your study
- Refer to the CTAT in the main text as ‘Supplementary CTAT Table’
- Do not add subheadings
- Add as many rows as needed to include all information
- Only include one item per row

**If the CTAT form is not relevant to your study, please outline the reasons why:**

| **N/A** |
| --- |

- 1. **Antibodies**

| **Name** | **Citation** | **Supplier** | **Cat no.** | **Clone no.** |
| --- | --- | --- | --- | --- |
| **N/A** |  |  |  |  |

- 1. **Cell lines**

| **Name** | **Citation** | **Supplier** | **Cat no.** | **Passage no.** | **Authentication test method** |
| --- | --- | --- | --- | --- | --- |
| **N/A** |  |  |  |  |  |

- 1. **Organisms**

| **Name** | **Citation** | **Supplier** | **Strain** | **Sex** | **Age** | **Overall n number** |
| --- | --- | --- | --- | --- | --- | --- |
| **N/A** |  |  |  |  |  |  |

- 1. **Sequence based reagents**

| **Name** | **Sequence** | **Supplier** |
| --- | --- | --- |
| **N/A** |  |  |

- 1. **Biological samples**

| **Description** | **Source** | **Identifier** |
| --- | --- | --- |
| **N/A** |  |  |

- 1. **Deposited data**

| **Name of repository** | **Identifier** | **Link** |
| --- | --- | --- |
| **ENA** | **Suppl. Table 12** | **https://www.ebi.ac.uk/ena/browser/home** |

- 1. **Software**

| **Software name** | **Manufacturer** | **Version** |
| --- | --- | --- |
| **R** | **R Core Team** | **(v4.3.1)** |

- 1. **Other (*e.g*. drugs, proteins, vectors etc.)**

| **N/A** |  |  |
| --- | --- | --- |
|  |  |  |

- 1. **Please provide the details of the corresponding methods author for the manuscript:**

| **Jonel Trebicka**  **Department of Internal Medicine B**  **University of Münster, Münster, Germany.**  **Albert-Schweitzer-Campus 1, Gebäude A1,**  **48149 Münster**  **Telephone number: +49 251 83-59689**  **Fax number: +49 251 83-47570**  **Electronic mail address: Jonel.Trebicka@ukmuenster.de** |
| --- |

**2.0 Please confirm for randomised controlled trials all versions of the clinical protocol are included in the submission. These will be published online as supplementary information.**

| **N/A** |
| --- |
