# Supplementary material for: Lactococcus A phages predict ACLF while Enterococcus B phages predict bacterial infection in decompensated cirrhosis
Source: JHEP Rep. 2025 Oct 10;8(1):101622. doi: 10.1016/j.jhepr.2025.101622 (PMC12721046; doi:10.1016/j.jhepr.2025.101622)
Supplement: Multimedia component 4 [file mmc4.pdf]

JHEP Reports

Lactococcus A phages predict ACLF while *Enterococcus B* phages predict bacterial infection in decompensated cirrhosis

Authors

Lore Van Espen, Maximilian Joseph Brol, Lila Close, …, Bernd Schnabl, Jonel Trebicka, Jelle Matthijnssens

Correspondence

jelle.matthijnssens@kuleuven.be (J. Matthijnssens), Jonel.Trebicka@ukmuenster.de (J. Trebicka).

Graphical abstract

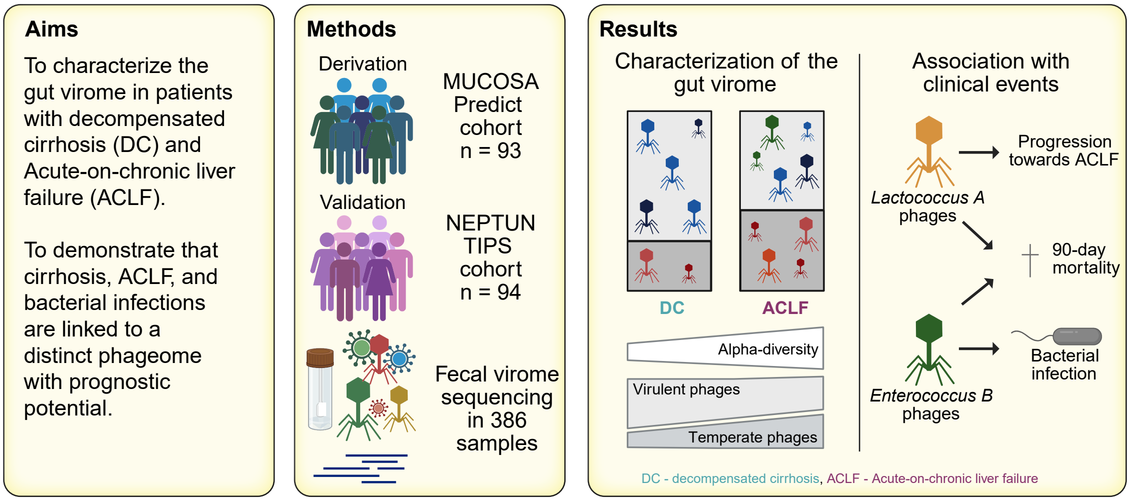

Highlights:

- ACLF is associated with a higher fecal virome alpha-diversity.
- ACLF onset is associated with a shift from virulent to temperate phages.
- *Lactococcus A* phages are associated with disease progression towards ACLF.
- *Enterococcus B* phages are associated with systemic bacterial infections.
- Both *Lactococcus A* and *Enterococcus B* phages are associated with increased 90-day mortality.

Impact and implications:

The human gut virome is a poorly investigated part of the human gut microbiome, especially in the context of decompensated cirrhosis and acute-on-chronic liver failure. This study identified two phage groups (*Lactococcus A* phages and *Enterococcus B* phages) with particular prognostic value. In the future, virome analysis of fecal samples could be useful for patient stratification in clinical practice.

https://doi.org/10.1016/j.jhepr.2025.101622

© 2025 The Author(s). Published by Elsevier B.V. on behalf of European Association for the Study of the Liver (EASL). This is an open access article under the CC BY license (http://creativecommons.org/licenses/by/4.0/).

Lactococcus A phages predict ACLF while *Enterococcus B* phages predict bacterial infection in decompensated cirrhosis

Lore Van Espen[1,†], **Maximilian Joseph Brol**[2,†], Lila Close[1], Robert Schierwagen[2], Wenyi Gu[2], Marisa I. Keller[3], Boglarka Balogh[4], Anthony Fullam[3], Lander De Coninck[1], Tomohiro Nakamura[5], Michael Kuhn[3], Peer Bork[3,6,7], Wim Laleman[2,8], Jasmohan S. Bajaj[9], Maria Papp[4], Bernd Schnabl[5], Jonel Trebicka[2,10,11,*], Jelle Matthijnssens[1,*], on behalf of MICROB-PREDICT

JHEP Reports **2026**. vol. 8 | 1–13

Check for updates

Background & Aims: As portal hypertension progresses in cirrhosis, bacterial translocation across a compromised gut barrier leads to endotoxemia, systemic inflammation and immune dysfunction. Gut phages play a key role in these processes by influencing bacteria-host interactions. This study explores the role of the human gut virome in acute decompensation of cirrhosis and acute-on-chronic liver failure (ACLF).

Methods: The fecal virome was longitudinally assessed by metagenomic sequencing in two independent cohorts: 93 patients (292 samples) with acute decompensation or ACLF from the PREDICT study, and 94 patients (94 samples) with decompensated cirrhosis undergoing TIPS (transjugular intrahepatic portosystemic shunt) surgery collected in a tertiary care setting. Besides descriptive analysis, phages were grouped according to their predicted bacterial host and lifestyle, and associated with clinical parameters.

Results: Phage alpha-diversity was higher in patients with ACLF and correlated with ACLF severity. In the absence of ACLF, the phageome was dominated by virulent phages, but in ACLF, temperate phages became more prevalent. Genus-level analysis showed that phageomes were highly patient-specific. *Lactococcus A* phages were the only phage-host group predicting ACLF development (odds ratio [OR] = 14; Fisher test p = 0.0129). *Enterococcus B* phages (OR = 14.7; p = 0.0015; adj. p = 0.037) and their bacterial hosts (OR = 2.8; p = 0.020) were significantly more prevalent in cases of proven systemic bacterial infection. The presence of both phage families was linked to increased 90-day mortality rates.

Conclusion: ACLF is characterized by increased fecal virome diversity and a shift from virulent toward temperate phages at disease onset. Our study links *Lactococcus A* phages to ACLF development, and *Enterococcus B* phages to bacterial infection, while both are associated with increased 90-day mortality.

Clinical trial number: NCT03056612.

© 2025 The Author(s). Published by Elsevier B.V. on behalf of European Association for the Study of the Liver (EASL). This is an open access article under the CC BY license (http://creativecommons.org/licenses/by/4.0/).

Introduction

Decompensated cirrhosis and its progression towards acute-on-chronic liver failure (ACLF) are a major cause of death in patients with chronic liver diseases.[1,2] The driving mechanisms for decompensation are portal hypertension and systemic inflammation, which are hastened amongst other factors by bacterial infections. The PREDICT study delineated three distinct courses in decompensated cirrhosis that differ significantly in terms of risk for ACLF development and mortality: mortality increases from stable decompensated cirrhosis through unstable decompensated cirrhosis, to pre-ACLF.[3,4] Understanding the complex mechanisms underlying the transition from decompensated cirrhosis to ACLF is essential for improving patient outcomes. Bacterial infections and alcohol-associated hepatitis have been identified as the most important triggers of ACLF.[5] However, in 30-40% of patients who develop ACLF no precipitating event can be detected. In these patients, it is presumed that the trigger for the development of ACLF is the translocation of gut microbes across an impaired gut barrier.[6]

The gut virome refers to the genomes of a diverse community of commensal viruses residing in the gastrointestinal tract, typically comprising a small number of eukaryotic viruses and a larger proportion of bacteriophages that infect the

* Corresponding authors. Addresses: KU Leuven, Department of Microbiology, Immunology, & Transplantation, Rega Institute, Division of Clinical & Epidemiological Virology, Laboratory of Viral Metagenomics, Herestraat 49 box 1040, 3000 Leuven, Belgium. Telephone number: +32 16 321161. Fax number: +32 16 330026. (J. Matthijnssens), or Department of Internal Medicine B, University of Münster, Münster, Germany. Albert-Schweitzer-Campus 1, Gebäude A1, 48149 Münster. Telephone number: +49 251 83-59689. Fax number: +49 251 83-47570. (J. Trebicka).
E-mail addresses: jelle.matthijnssens@kuleuven.be (J. Matthijnssens), Jonel.Trebicka@ukmuenster.de (J. Trebicka).
† Authors contributed equally
https://doi.org/10.1016/j.jhepr.2025.101622

bacterial component of the gut microbiota. Phages can affect the human host through their close interactions with the gut bacteria and are known to be relevant in human health and disease.[7] For example, phages can alter the composition of the gut bacterial community through bacterial lysis as part of the phage replication cycle.[8] Temperate phages can mediate horizontal gene transfer between bacteria, potentially shaping the functional properties of the gut bacteria including anti-microbial resistance.[9] Apart from these indirect effects on human health through the gut bacterial community, phages can also directly engage with the human host via its immune system.[10,11] Recent bioinformatic tool developments in the field of viral metagenomics have enabled comprehensive profiling of the gut virome, including better identification of phages[12,13] and bacterial host prediction.[14,15] Phage host prediction allows for an improved biological interpretation, as changes in phages infecting a distinct group of bacteria can shed light on the specific phage-bacteria dynamics of the gut microbiome.

While extensive research has focused on characterizing alterations of gut bacteria in relation to decompensated cirrhosis and its complications,[16,17] little is known about interactions of the virome with progression of decompensated cirrhosis, though the virome has been implicated in other types and stages of chronic liver disease.[18–22] For example, progression of decompensated cirrhosis is associated with increased abundance of phages infecting *Enterobacteriaceae* and *Lactobacillaceae* bacteria and decreased abundance of crAssphages, a bacteriophage family representing the most abundant viruses in the human gut.[18] In mice, phages targeting cytolysin-positive *Enterococcus faecalis* reversed the exacerbation of liver disease[23] and phages targeting ethanol-producing *Klebsiella pneumoniae* prevented the development of liver disease.[24,25] Further research into bacterial and viral dysbiosis could help identify other potential targets for phage therapy.[26] However, it is still unclear which virome perturbations are associated with decompensated cirrhosis and ACLF and whether phages can be used as biomarkers for the onset of ACLF in patients with cirrhosis. This study aims to address this knowledge gap by conducting an extensive analysis of the gut virome in patients with decompensated cirrhosis and ACLF. We hypothesized patients with cirrhosis, ACLF and bacterial infections have a distinct phageome that could be leveraged for prognostication.

Patients and methods

Study design, data and sample collection

The samples of the derivation cohort were obtained from 93 patients recruited at the University of Debrecen (Hungary) as part of the PREDICT study (MUCOSA-PREDICT sub-study) (ClinicalTrials.gov number, NCT03056612). The PREDICT study is a European, investigator-initiated, multicenter, prospective, observational study.[54] The study was approved by the local ethics committee (35281-2/2017/EKU), and all patients signed an informed written consent in accordance with the Helsinki Declaration. Acute decompensation and ACLF were diagnosed as previously described according to the European Association for the Study of the Liver – Chronic Liver Failure (EASL-CLIF) criteria.[1,27] Patients were stratified into stable decompensated cirrhosis (n = 53), unstable decompensated cirrhosis (n = 10), pre-ACLF (n = 21) and ACLF (n = 9) based on previously described criteria.[4] Patients were retrospectively assigned to the pre-ACLF group if they developed ACLF within the follow-up but did not have ACLF at enrollment. Clinical and laboratory data, as well as biological samples, were collected at multiple time points[4] (Fig. S1). Briefly, patients were stratified according to their Chronic Liver Failure Consortium (CLIF-C) acute decompensation (AD) score (high risk ≥50; low risk <50). The high-risk group had scheduled follow-up after 1, 4, 8 and 12 weeks, while the low-risk group was followed up solely at week 1 and 12. All patients who developed ACLF during the observational period had two additional unscheduled visits (ACLF onset visit and another 7 days later).

The samples for the validation cohort were obtained from 98 patients with decompensated cirrhosis who received a transjugular intrahepatic portosystemic shunt (TIPS) as part of the NEPTUN study (NCT03628807) at the Department of Internal Medicine I, University Clinic Bonn (Germany).[28–30] The stool samples were collected during inpatient treatment before TIPS placement and stored immediately at -80 °C degrees until analysis. The study was approved by the local ethics committee of the University of Bonn (029/13), and all patients signed an informed written consent in accordance with the Helsinki Declaration. Fecal samples were processed for virome sequencing in the same manner as the MUCOSA-PREDICT samples.[31] Bioinformatic processing of sequencing reads (including quality control, assembly, viral identification, abundance determination, lifestyle and host prediction) was performed in the same fashion as the MUCOSA-PREDICT samples. Good-quality fecal virome profiles were generated for 94 samples.

Fecal virome profiling

Viral-like particles were purified from fecal samples and prepared for virome sequencing using the NetoVIR protocol.[31] Sequencing reads were quality-controlled and assembled, followed by clustering of the resulting scaffolds >1 kb at 95% identity over 85% coverage to remove cross-sample redundancy. Viral scaffolds were identified using a combination of VirSorter2[12] and similarity to known viruses using DIAMOND[32] and/or BLASTn,[33] and their genome completeness was assessed using CheckV.[34] A horizontal coverage cut-off of 70% was applied to determine the presence of a scaffold in a sample. The host genus of a phage was predicted using a combination of methods (*e.g.* CRISPR similarity, tRNA similarity, prophage similarity, kmer sharing and protein content) relying on a database of bacterial metagenomic-assembled genomes (MAGs) from the same fecal samples and from a publicly available database.[35]

Methods on the profiling of the fecal bacteriome and more details on the virome profiling can be found in the supplementary methods and Fig. S2.

Ecological analyses, statistical analyses and visualization

Data analysis and visualization were performed in R (v4.3.1).[36] Statistical significance was defined as p <0.1 and correction for multiple hypothesis testing was applied where appropriate using the Benjamini-Hochberg method.[37]

Alpha-diversity (Simpson diversity) was compared between two groups of samples using a two-tailed Wilcoxon rank-sum test. Relative abundance of phages grouped by their lifestyle were compared between and within samples using two-tailed

(paired) Wilcoxon rank-sum tests. Associations of alpha-diversity and relative abundances of temperate and virulent phages with disease scores were analyzed using Pearson's correlation coefficient.

The inter-individual phageome variation was calculated using Bray-Curtis dissimilarity on the phage genus level and visualized using principal coordinate analysis. The contribution of the clinical covariates on the inter-individual phageome variation was determined using univariate distance-based redundancy analysis (dbRDA) using the *capscale* function.[38] Samples with missing data were excluded for the respective univariate analysis and only covariates with less than 10% missing data were included. Clinical covariates were transformed (log_{10}, inverse or square root) to better approach a normal distribution. The cumulative non-redundant contribution of clinical covariates on the phageome variation was determined using stepwise multivariate dbRDA (forward-selection model) using the *ordiR2step* function.[38] No correction for multiple testing was performed in the multivariate dbRDA since only variables with an adjusted *p* value <0.1 in the univariate dbRDA were included in the multivariate dbRDA. Samples with missing data for any of the significant univariate covariates were excluded.

As phages most likely affect the human host through their bacterial hosts, differential abundance analysis of phages was done by grouping them according to their predicted host genus for a more meaningful biological interpretation. Wilcoxon rank-sum tests were used to compare differences in relative abundance of phage-host groups present in at least five samples of interest. Differences in prevalence were assessed using Fisher's exact tests. Relative abundances of these phage-host groups were log10-transformed with the addition of a pseudocount (0.000001) for visualization purposes. A log-rank test was used to assess differences in short-term (90-day) survival between patients with and without phage-host groups present at any timepoint in at least five patients.

Results

The gut virome of patients with decompensated cirrhosis is dominated by microviruses and caudoviruses

The MUCOSA-PREDICT cohort consists of 93 patients, 9 of whom had ACLF at enrollment and 84 patients with AD. After a 90-day follow-up period, the patients with AD were retrospectively stratified into three groups: the pre-ACLF group (patients who developed ACLF; n = 21), the unstable

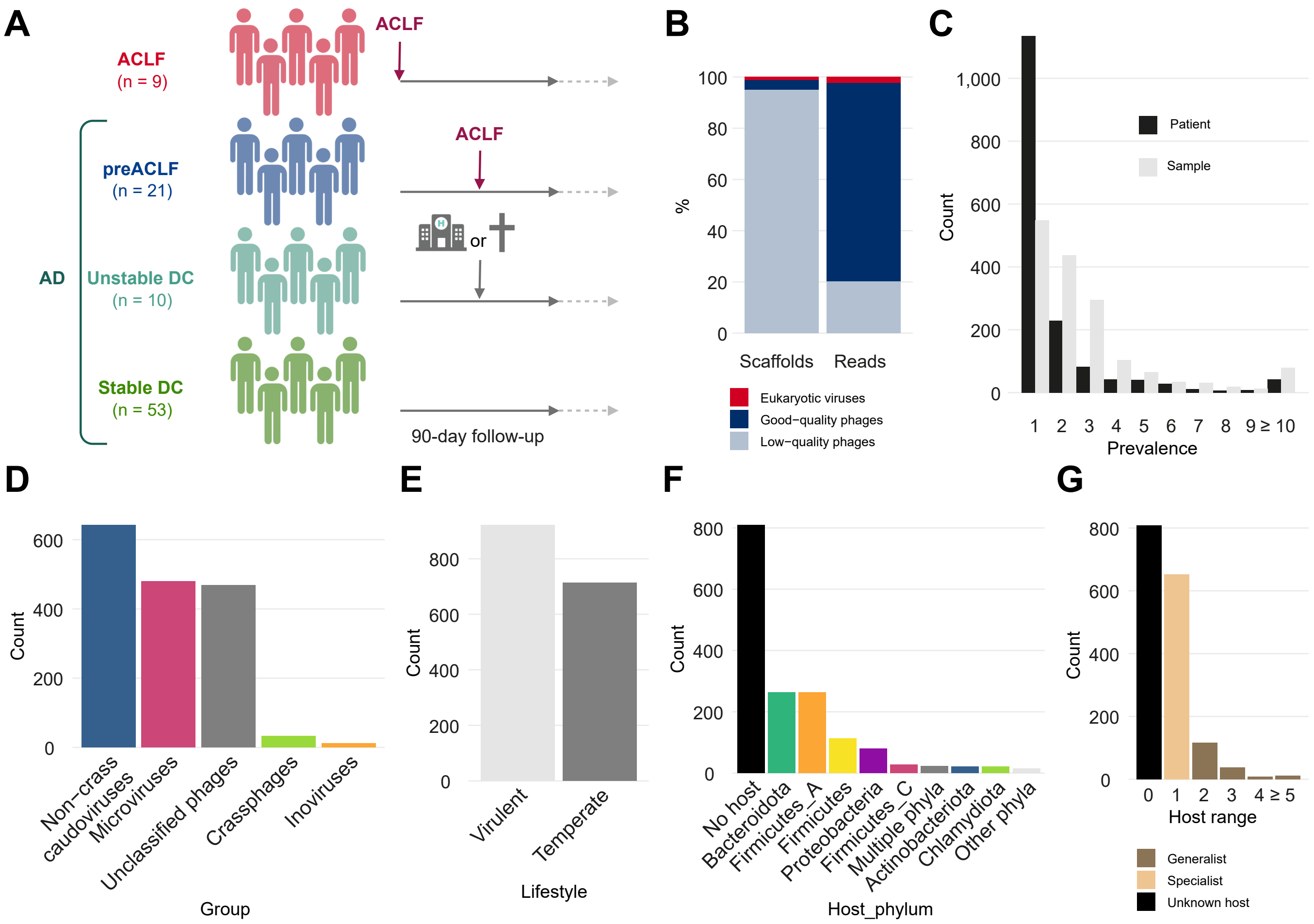

Fig. 1. Overview of the clinical cohort and virome dataset. (A) Overview of the clinical cohort. (B) Overview of virome composition at scaffold and read level in all 292 samples. (C) Distribution of good-quality phages (n = 1,635) according to their prevalence (number of patients and samples, respectively). (D-F) Classification of good-quality phages (n = 1,635) according to their predicted taxonomic classification (D), lifestyle (E), bacterial host (F) and host range on the genus level (G). ACLF, acute-on-chronic liver failure; AD, acute decompensation; DC, decompensated cirrhosis.

decompensated cirrhosis group (patients requiring hospital readmission and/or who died without developing ACLF; n = 10) and stable decompensated cirrhosis (patients who did not have any events during the 90 days; n = 53) (Fig. 1A). Good-quality virome profiles were generated for 292 samples, with an average of three samples per patient. Average disease scores increased from the stable to the unstable decompensated cirrhosis group and further to the pre-ACLF group (Table 1).

The fecal viromes were dominated by phages, as only 1% of viral scaffolds belonged to eukaryotic viruses, representing 2.5% of the viral reads (Fig. 1B). Further analysis of the virome was focused on the good-quality phages (*i.e.* estimated genome completeness >50%; see supplementary methods) to reduce noise from highly fragmented genomes. These good-quality phages (n = 1,635; 4% of phage scaffolds) represent the majority of phage reads (79%) (Fig. 1B). Most of the phage scaffolds were found in a single patient (69% phage scaffolds)

Table 1. Overview of patient characteristics.

	ACLF	Pre-ACLF	UDC	SDC	*p* value
Patients, n	9	21	10	53	
Samples, n	20	65	37	170	
Samples/Patient, mean ± SD	2.2 ± 0.4	3.1 ± 1.3	3.7 ± 1.2	3.2 ± 0.9	0.004
Sex (Male), n (%)	7 (77.8%)	14 (66.7%)	5 (50.0%)	31 (58.5%)	0.6
Age, mean ± SD	57.4 ± 12.7	63.4 ± 7.7	60.7 ± 13.9	57.6 ± 10.4	0.2
Etiology, n (%)					
Alcohol	9 (100%)	18 (86%)	8 (80%)	47 (89%)	0.6
Hepatitis B virus	0 (0%)	1 (4.8%)	1 (10%)	0 (0%)	0.2
Hepatitis C virus	1 (11%)	1 (4.8%)	0 (0%)	3 (5.7%)	0.7
MASLD	4 (44%)	3 (14%)	0 (0%)	5 (9.4%)	0.03
Cryptogenic	0 (0%)	1 (4.8%)	1 (10%)	1 (1.9%)	0.4
Other	1 (11%)	1 (4.8%)	0 (0%)	3 (5.7%)	0.7
**Baseline**					
Disease scores, mean ± SD [NAs]					
MELD score	19.3 ± 5.9	17.9 ± 6.0	13.9 ± 5.4	13.7 ± 5.1 [1]	0.006
MELD-Na score	22.2 ± 5.7	22.7 ± 7.0	17.7 ± 6.7	16.7 ± 5.7 [1]	0.002
Child-Pugh score	7.8 ± 1.9 [1]	9.4 ± 1.7	9.1 ± 1.7	8.5 ± 1.9 [3]	0.076
CLIF-C ACLF score	43.7 ± 7.3	NA	NA	NA	NA
CLIF-C AD score	NA	57.9 ± 7.8	54.2 ± 7.3	49.1 ± 7.4 [1]	<0.001
Medication, n (%)					
Albumin	1 (11%)	3 (14%)	4 (40%)	5 (9.4%)	0.11
Antibiotics (all)	6 (67%)	10 (48%)	6 (60%)	22 (42%)	0.5
Rifaximin	1 (11%)	1 (4.8%)	0 (0%)	4 (7.5%)	0.8
Lactulose	4 (44%)	2 (9.5%)	1 (10%)	7 (13%)	0.11
Proton pump inhibitors	5 (56%)	7 (33%)	5 (50%)	14 (26%)	0.2
**1 week follow-up** (4-10 days)					
Disease scores, mean ± SD [NAs]					
MELD score	14.1 ± 9.1	17.1 ± 8.5	12.9 ± 5.4	12.4 ± 4.5	0.3
MELD-Na score	17.3 ± 7.9	21.3 ± 7.8	18.2 ± 5.6	15.7 ± 5.7	0.043
Child-Pugh score	7.9 ± 2.0 [2]	8.9 ± 1.8 [7]	8.8 ± 2.0 [1]	7.9 ± 1.6 [11]	0.186
CLIF-C ACLF score	44.5 ± 12.0 [8]	42.8 ± 6.2 [17]	NA	NA	NA
CLIF-C AD score	50.7 ± 3.5 [3]	54.7 ± 7.2 [8]	53.3 ± 7.9	48.5 ± 8.2 [3]	0.033
Medication, n (%) [NAs]					
Albumin	4 (40%)	7 (39%) [3]	3 (30%)	5 (9.4%) [1]	0.11
Antibiotics (all)	9 (90%)	10 (48%) [3]	6 (60%)	22 (42%) [1]	0.5
Rifaximin	1 (11%)	1 (4.8%) [3]	0 (0%)	4 (7.5%) [1]	0.8
Lactulose	4 (44%)	2 (9.5%) [3]	1 (10%)	7 (13%) [1]	0.11
Proton pump inhibitors	7 (70%)	7 (39%) [3]	6 (60%)	19 (37%) [1]	0.12
**All samples from all patients**					
Disease scores, mean ± SD [NAs]					
MELD score	16.6 ± 7.8 [1]	19.3 ± 7.9 [1]	13.1 ± 4.9	12.2 ± 4.4	<0.001
MELD-Na score	20.0 ± 7.0 [1]	23.8 ± 7.4 [1]	17.6 ± 5.8	15.3 ± 5.3	<0.001
Child-Pugh score	7.8 ± 1.8 [4]	9.2 ± 2.0 [5]	8.9 ± 1.8	7.9 ± 1.8 [38]	<0.001
CLIF-C ACLF score	43.5 ± 8.0 [9]	47.8 ± 9.9 [49]	NA	NA	0.5
CLIF-C AD score	51.9 ± 4.6 [12]	57.0 ± 8.4 [17]	52.1 ± 7.3	47.9 ± 7.6	<0.001
Medication, n (%)					
Albumin	5 (25%)	22 (34%)	13 (35%)	9 (5.3%)	<0.001
Antibiotics (all)	15 (75%)	34 (52%)	28 (76%)	70 (41%)	<0.001
Rifaximin	2 (10%)	12 (18%)	3 (8.1%)	12 (7.1%)	0.076
Lactulose	8 (40%)	18 (28%)	6 (16%)	28 (16%)	0.035
Proton pump inhibitors	12 (60%)	21 (32%)	15 (41%)	49 (29%)	0.006

Numbers between squared brackets indicate the number of individuals for whom the variable was NA. Continuous variables are represented as mean ± standard error and compared between groups using Kruskal-Wallis tests. Categorical variables are represented as counts with percentages between brackets and compared between both groups using Fisher's exact test. ACLF, acute-on-chronic liver failure; AD, acute decompensation; CLIF-C, Chronic Liver Failure Consortium; MASLD, metabolic dysfunction-associated steatotic liver disease; MELD, model for end-stage liver disease; NA, not available; SDC, stable decompensated cirrhosis; UDC, unstable decompensated cirrhosis.

or even a single sample (34% phage scaffolds) (Fig. 1C), while only three phage scaffolds were present in more than half of the patients ("core" phages).[39]

Most of the good-quality phage scaffolds were classified as microviruses (icosahedral single-stranded DNA viruses; 29% phage scaffolds) and caudoviruses (tailed double-stranded DNA viruses; 41% phage scaffolds), including 33 crAssphages (Fig. 1D). The majority of phages (56%) were predicted to be virulent and were therefore unable to integrate their genome into that of their host in contrast to temperate phages (Fig. 1E). Almost a third of the phage scaffolds remained unclassified (29% phage scaffolds), potentially including numerous previously undescribed phages.

Bacteria belonging to the *Bacteroidota* and *Firmicutes C* phyla were the most common predicted hosts of the phages (n = 263 for both phyla; Fig. 1F), which was expected given the dominance of these phyla in the human gut.[40] Of the 51% of the phages for which a host could be predicted, 174 were linked to multiple bacterial genera (generalist phages; 11% phage scaffolds) compared to 652 specialist phages predicted to infect a single bacterial genus (specialist phages; 40% phage scaffolds) (Fig. 1G). The potential broad host range of human gut phages has been described elsewhere.[41,42]

Inter-sample phageome variation is mainly driven by interpersonal differences

The fecal phage community was profiled at the genus level to explore the inter-sample phageome variation (Bray-Curtis dissimilarity). The limited explained variance on the first two axes of the principal coordinate analysis plot highlight the high inter-sample diversity in this cohort (Fig. 2A). To determine which clinical covariates explained this high inter-sample diversity in phage community, the individual effect size of each covariate (n = 40) was determined using univariate distance-

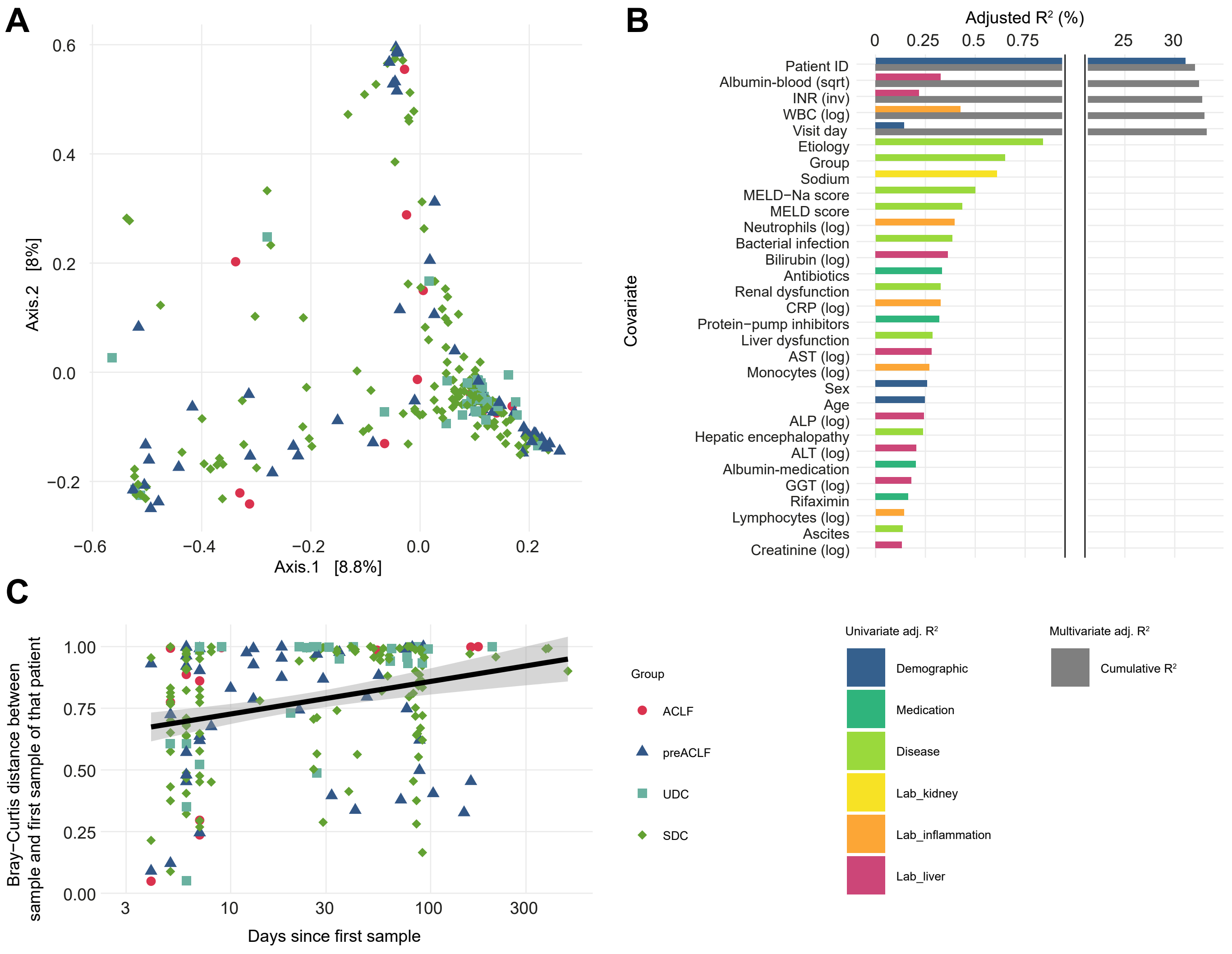

Fig. 2. Beta-diversity of the phage community in patients with decompensated cirrhosis and ACLF. (A) PCoA plot of the gut phage community of all samples from all patients colored by disease group (n = 292) (Bray-Curtis distance at the genus level). (B) Individual effect sizes (colored top bars) of covariates on the genus-level phage ordination (univariate dbRDA; n = 31 with FDR <0.1) and cumulative effect sizes (grey bottom bar) of non-redundant covariates (multivariate dbRDA; *p* <0.1) selected by stepwise feature selection based on 268 samples. Covariates are ordered based on cumulative R^2 for covariates in the multivariate model and decreasing univariate R^2 for the remaining covariates. (C) Association between days since the first available visit and distance to the first available visit (n = 199, excluding the first available visit (n = 93) of each patient). ACLF, acute-on-chronic liver failure; dbRDA, distance-based redundancy analysis; FDR, false discovery rate; PCoA, principal coordinate analysis; SDC, stable decompensated cirrhosis; UDC, unstable decompensated cirrhosis.

based redundancy analysis (Table S1). The patient identifier was unequivocally the covariate with the most considerable effect on the phage community (univariate R^2 = 31%). In addition, 30 other covariates such as blood values, disease scores, complications and medication had a limited effect on phageome composition (univariate R^2 <1%; false discovery rate <0.1; colored bars in Fig. 2B).

All covariates significantly associated with the phage ordination (n = 31/40; false discovery rate <0.1) were subsequently fed into a stepwise forward-selection model to determine the cumulative effect sizes of non-redundant covariates on the phage community composition. For this multivariate analysis, if any of the included covariates were unavailable for a given sample then that sample was removed (n = 24). The removal of these samples resulted in a slightly higher effect of the patient identifier on the phage community of the remaining 268 samples (R^2 = 32%). Only a few other covariates had a limited additional non-redundant effect on phage community composition. Serum albumin levels (R^2 = 0.4%), international normalized ratio (R^2 = 0.3%), and white blood cell count (R^2 = 0.2%) significantly explained an additional fraction of the phage community variation. Visit day, representing the number of days since the first visit, was the last covariate (R^2 = 0.2%) that significantly contributed to the cumulative effect of all covariates on the phage community (cumulative R^2 = 33.2%; grey bars in Fig. 2B).

The impact of time on the phage composition is further highlighted by the correlation between days since the first visit and the distance between the samples and the first available visit of each patient (r_p = 0.19; p = 0.005; Fig. 2C). This means that the longer the time since the first visit, the more the phage community has diverged from the initial one. Nonetheless, the high inter-sample variation of the phage community remains largely unexplained, as included covariates contributed to only 33.2% of the overall phage community variation.

Higher phage Simpson diversity associated with ACLF and its mortality

We next assessed the changes in phage alpha-diversity (Simpson diversity) in relation to the presence of ACLF. Firstly, alpha-diversity of the phageome tends to be higher in patients with ACLF compared to patients without ACLF (Wilcoxon rank-sum test; p = 0.068; Fig. 3A). Here, the first ACLF visits from patients in the ACLF (n = 9) and pre-ACLF groups (n = 14) were compared to the first visits of patients from the stable (n = 10) and unstable (n = 53) decompensated cirrhosis groups. When considering all available samples from all patients regardless of disease group, higher phage diversity was observed in ACLF samples, though not reaching statistical significance (Wilcoxon rank-sum test; p = 0.096; Fig. S3). Furthermore, phage alpha-diversity is increased when patients from the pre-ACLF group progress from decompensated cirrhosis to ACLF (Wilcoxon singed-rank test; p = 0.023; n = 14; Fig. 3B).

Moreover, alpha-diversity of the gut phageome was positively correlated with the CLIF-C ACLF score, for a surrogate of ACLF-associated mortality, in the sample at ACLF development, without reaching statistical significance (r_p = 0.41; p = 0.056; n = 22; Fig. 3C) as well as across all ACLF samples from all patients (r_p = 0.47; p = 0.013; n = 27; Fig. S4A). No association of phage diversity with the model for end-stage liver disease (MELD) score was observed (p = 0.61; n = 290; Fig. S4B). Finally, neither the use of proton pump inhibitors nor of antibiotics was associated with statistically significant differences in alpha-diversity in gut viromes of patients with ACLF (p = 0.11 and p = 0.7, respectively, Fig. S5). These findings indicate that phage alpha-diversity might be elevated in the presence of ACLF and positively linked to the development and severity of ACLF.

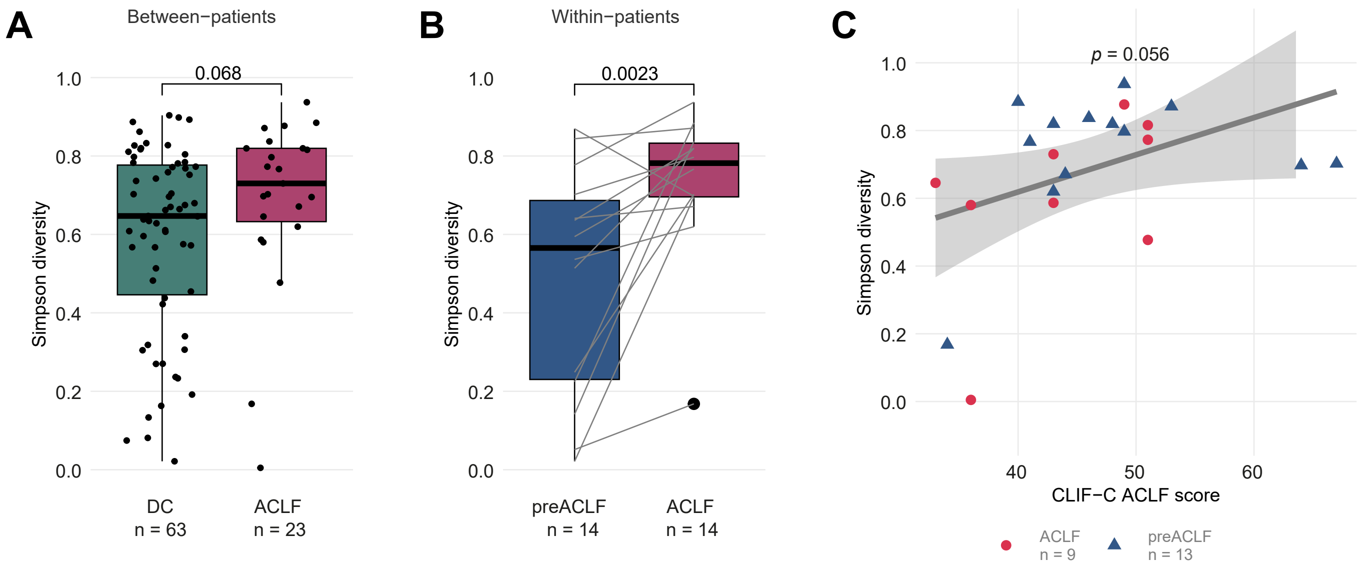

Fig. 3. Phage Simpson diversity associated with ACLF progression and its mortality. (A) Inter-individual comparison of phage alpha-diversity between the first sample of patients with stable and unstable decompensated cirrhosis and the first ACLF sample of (pre)-ACLF patients (Wilcoxon rank-sum test). (B) Intra-individual comparison of phage alpha-diversity between the first sample and the first ACLF sample in pre-ACLF patients (Wilcoxon signed-rank test). Lines connect samples from the same patient. (C) Relationship between phage alpha-diversity and CLIF-C ACLF score across the sample of each (pre)ACLF patient at ACLF development (Pearson's correlation coefficient = 0.41). All samples were derived from the visit of ACLF onset. ACLF, acute-on-chronic liver failure; CLIF-C, Chronic Liver Failure Consortium; DC, decompensated cirrhosis.

Disease progression associated with increased relative abundance of temperate phages

In addition to the observed changes in viral diversity, we assessed whether progression towards ACLF was also associated with the composition of the gut phageome in terms of phage lifestyle. Temperate phages can integrate their genomes into their bacterial host genome and replicate with their host (lysogenic lifecycle), in addition to following a lytic lifecycle. Healthy adult viromes are dominated by virulent phages, which can only replicate using the lytic lifecycle.[43] Gut phageomes of the first sample of patients with decompensated cirrhosis were likewise dominated by virulent phages, with limited presence of temperate phages (Fig. 4A). In ACLF viromes (first ACLF visit of (pre)ACLF patients), on the other hand, the virulent phages still dominated, but the difference was not statistically significant. The dominance of virulent phages was also observed in pre-ACLF patients before the onset of ACLF (Fig. 4B). At ACLF onset, however, this predominance of virulent phages was again diminished, at the expense of temperate phages. A similar observation was made in all available samples when comparing samples with and without ACLF (Fig. S6).

Although the elevated levels of temperate phages in ACLF viromes are not significantly different from those in non-ACLF viromes (Figs 4A,B, and S6), their relative abundance, although also not significant, correlated positively (r_p = 0.19; n = 93; Fig. 4C left) with the MELD score, as a marker for disease severity, in the first sample of each patient. The relative abundance of temperate and virulent phages furthermore associated positively and negatively, respectively, with MELD score when considering all available samples (r_p = 0.17 and

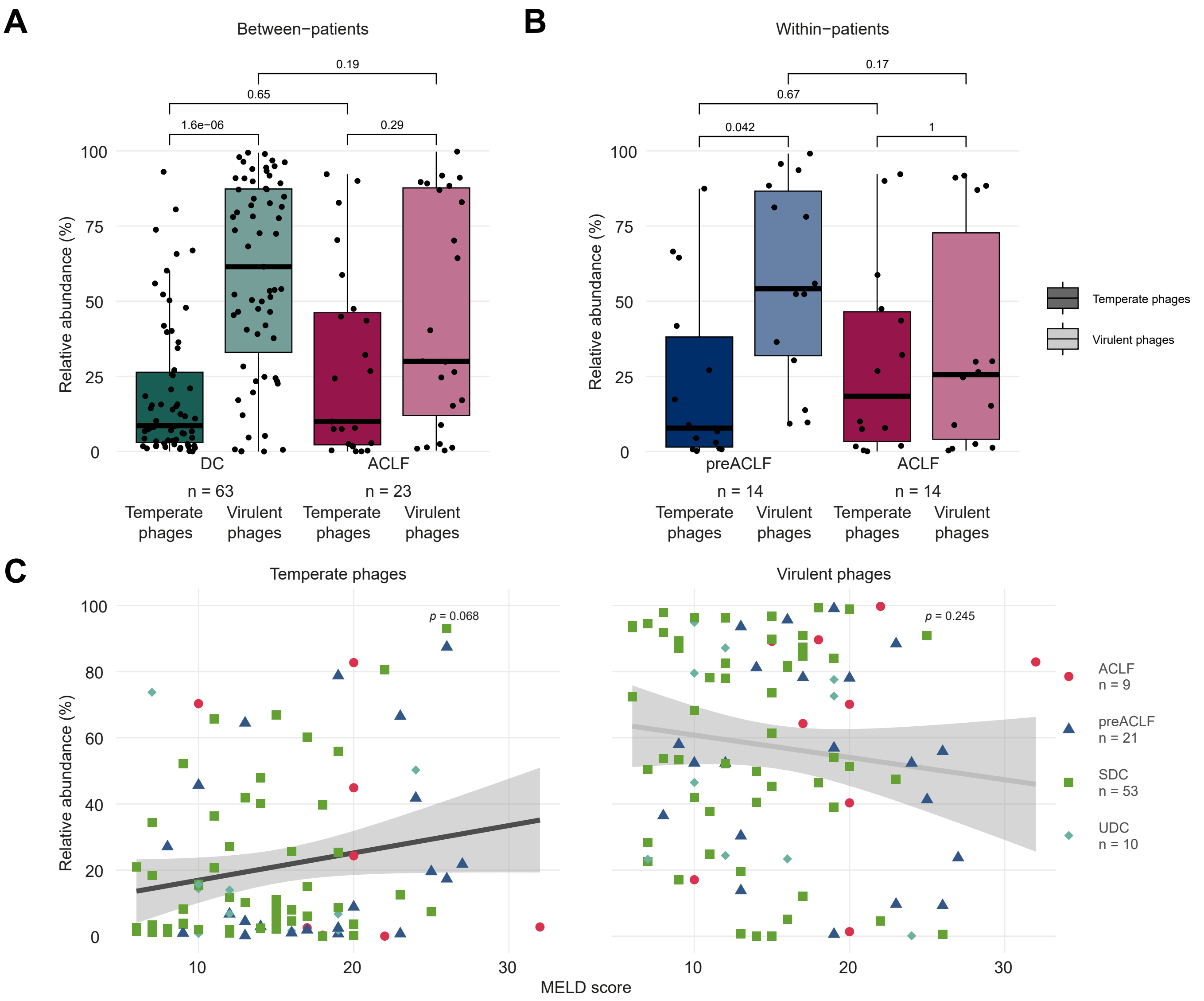

Fig. 4. Disease progression is marked by a shift in phageome composition in terms of phage lifestyle. (A) Phageome composition in terms of phage lifestyle in the first sample of patients with stable and unstable decompensated cirrhosis and the first ACLF samples of (pre)-ACLF patients (Wilcoxon rank-sum tests). (B) Phageome composition in terms of phage lifestyle between the first sample and the first ACLF sample of pre-ACLF patients (Wilcoxon signed-rank tests). (C) Relationship between MELD score and relative abundance of temperate (left panel) and virulent (right panel) phages in the first visit of each patient (Pearson's correlation coefficient = 0.19 and -0.12 (n.s.)). ACLF, acute-on-chronic liver failure; DC, decompensated cirrhosis; MELD, model for end-stage liver disease.

r_p = -0.17; n = 290; Fig. S7A), but not CLIF-C ACLF score (p = 0.82 and p = 0.56, respectively; n = 27; Fig. S7B). These results suggest that disease progression is associated with a shift from a virulent-dominated gut virome towards a phageome with a lower relative abundance of virulent phages.

Lactococcus A phages predict ACLF development and are associated with higher short-term mortality

Phages likely exert their effects on the human host indirectly through their bacterial hosts; therefore, specific phages have been associated with clinical features by grouping them according to their predicted host genus for a more meaningful biological interpretation. We assessed whether any specific phage-host groups (n = 25) were enriched in patients with pre-ACLF (n = 21) compared to those with stable and unstable decompensated cirrhosis (n = 63) at their first visit (Fig. 5A, Table S2). *Lactococcus A* phages were the only group of phages significantly more abundant in the first samples of patients with pre-ACLF (Fig. 5B). However, their increased prevalence in the first visit of patients with pre-ACLF was no longer significant after correction for multiple hypothesis testing (Fisher test: p = 0.01287; adj. p = 0.32181; odds ratio [OR] = 14; 1/63 *vs.* 4/21, Table S2). *Lactococcus A* phages were also more abundant when considering all samples of patients with pre-ACLF before ACLF onset (n = 39) compared to all samples from patients with decompensated cirrhosis (n = 207) (p <0.0001; Fig. S7). In contrast, the abundance and prevalence of *Lactococcus A* bacteria was not increased in pre-ACLF compared to stable and unstable decompensated cirrhosis (Fig. S9A; Fisher test: p = 1; OR = 1; 9/63 *vs.* 3/21).

The group of *Lactococcus A* phages associated with future ACLF development consists of two partial caudoviral genomes predicted to be temperate (Table S3). A potential explanation for the increased abundance of these temperate *Lactococcus A* phages without a simultaneous increase in *Lactococcus A* bacteria is an induction of these phages from their integrated prophage state. One of the *Lactococcus A* phage representatives showed high similarity (99.8%) to a *Lactococcus A* bacterial MAG, assembled from a patient prior to ACLF onset. In that same sample, three fragments of that *Lactococcus A* phage (1.8 kb, 1.9 kb and 6.4 kb) were found to be nearly identical (1 mismatch) to the *Lactococcus A* bacterial MAG, potentially indicating a recent induction, although it cannot be excluded that parts of the prophage were sequenced instead of the extracellular phage. Inflammation levels have previously been associated with the induction of prophages into their extracellular form.[44] The inflammation preceding and associated with ACLF onset might therefore contribute to the induction of these *Lactococcus A* phages.

Furthermore, the association between short-term (90-day) mortality and the presence of phage-host groups (n = 40) identified two mortality-associated phage-host groups: *Lactococcus A* phages and *Enterococcus B* phages (Table S4). Out of the 13 patients harboring *Lactococcus A* phages in any of the investigated longitudinal samples during the 90-day follow-up period, seven patients died following ACLF development (Fig. 5C). The presence of *Lactococcus A* bacteria was also associated with worse short-term survival (Fig. S9B). Combined with the previous section, these results suggest inflammation-induced activation of prophages might be an important factor implicated in the progression of decompensated cirrhosis. More specifically, *Lactococcus A* phages are an important group of phages in patients prior to ACLF development and a strong marker for poor short-term survival.

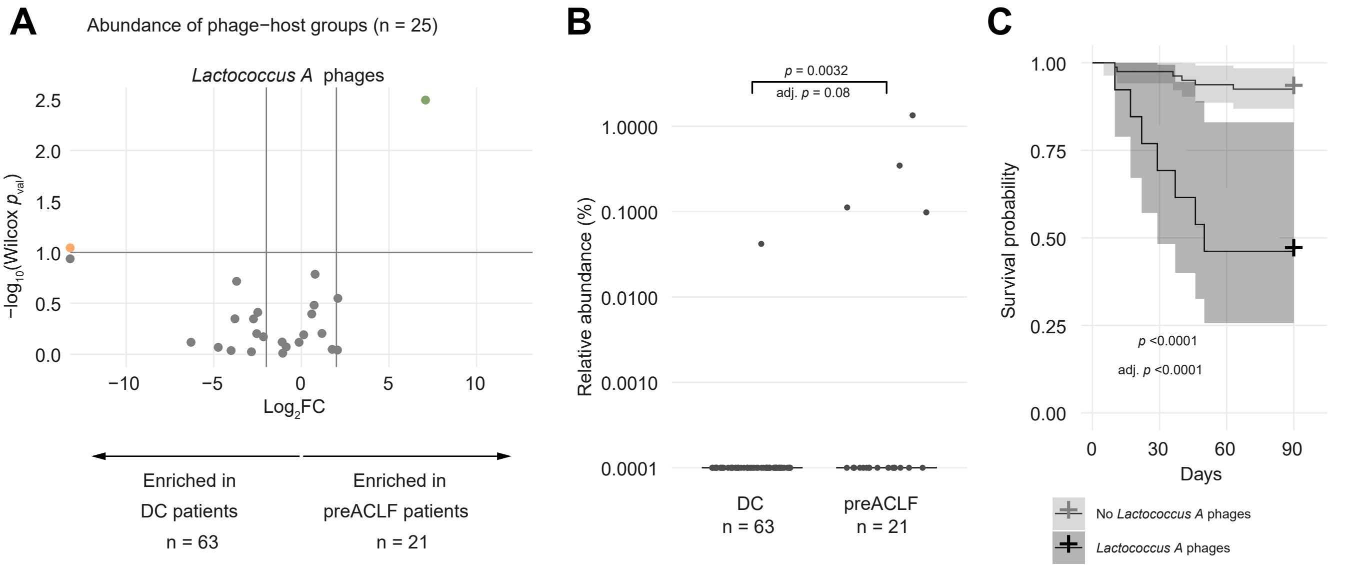

Fig. 5. *Lactococcus A* phages associated with pre-ACLF and higher short-term mortality. (A) Volcano plot to visualize changes in abundance of phage-host groups (n = 25) between pre-ACLF and stable and unstable decompensated cirrhosis present in the first sample of at least 5/84 patients (Wilcoxon rank-sum test; orange dots = p <0.1; green dots = FDR <0.1). (B) Relative abundance of *Lactococcus A* phages in the first visit of decompensated cirrhosis *vs.* pre-ACLF (Wilcoxon rank-sum test; FDR-adjusted for 25 phage-host groups present in the first sample of at least 5/84 patients). (C) 90-day survival analysis of patients with (n = 14) and without (n = 79) *Lactococcus A* phages (first sample with these phages within 90 days from first sample *vs.* first sample) (Log-rank test; FDR-adjusted for 40 phage-host groups present in at least 5/93 patients at any timepoint). ACLF, acute-on-chronic liver failure; DC, decompensated cirrhosis; FDR, false discovery rate.

Enterococcus B phages are associated with proven bacterial infection and higher short-term mortality

Bacterial translocation to the portal system and systemic bacterial infections are important precipitating events in the progression of decompensated cirrhosis.[5] Therefore, we assessed if any phage-host group (n = 25) was enriched in patients with a bacterial infection, given the close interaction between phages and bacteria (Table S5, Fig. 6A). *Enterococcus B* phages were the only phage-host group enriched in the first available sample of patients with proven bacterial infection compared to the first sample of patients without active or future bacterial infection (Fig. 6B). The increased abundance of these phages coincided with elevated levels of *Enterococcus B* bacteria in patients experiencing bacterial infection (Fig. S10A), in line with previous observations.[45] The relative abundance of *Enterococcus B* is higher in all patients with bacterial infections, reaching significance only in patients with pre-ACLF and stable decompensated cirrhosis (Fig. S10B). Both *Enterococcus B* phages and the bacteria were also more prevalent in these patients (Fisher test phages: p = 0.00149; adj. p = 0.03734; OR = 14.7; 15/53 *vs.* 1/40; Fisher test bacteria: p = 0.02009; OR = 2.8; 34/53 *vs.* 15/39). Most of the *Enterococcus B* phages are predicted to be temperate and therefore theoretically able to integrate into the genomes of their bacterial hosts, mainly *E. faecium*, *E. hirae* and *E. lactis* (Table S6).

Apart from *Lactococcus A* phages, *Enterococcus B* phages were the only other group of phages associated with a worse 90-day survival rate (Fig. 6C). Moreover, the presence of *Enterococcus B* bacteria was associated with worse 90-day survival in these patients (Fig. S10C). In conclusion, *Enterococcus B* phages are strongly associated with bacterial

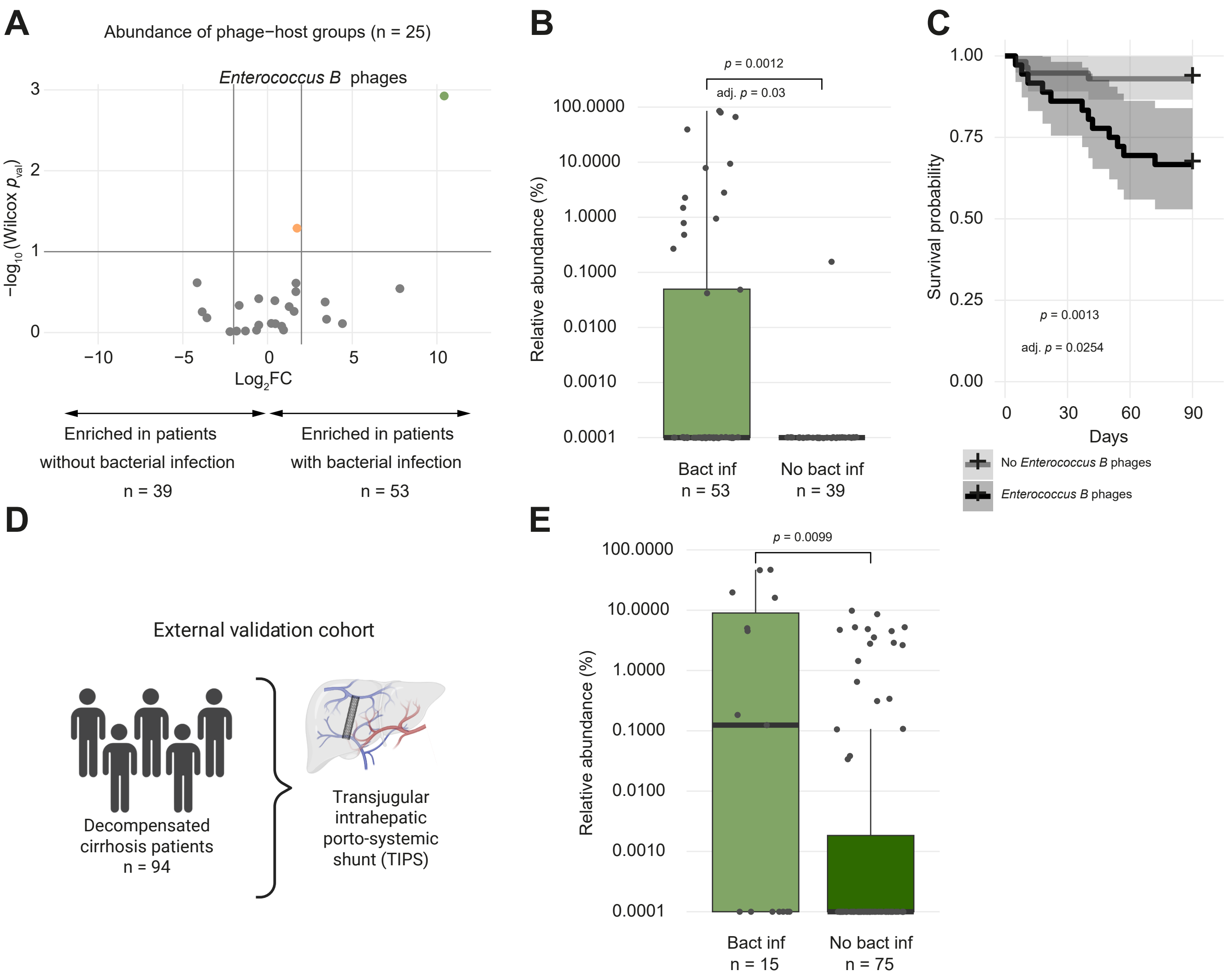

Fig. 6. *Enterococcus B* phages are associated with bacterial infection and worse short-term survival. (A) Volcano plot to visualize changes in abundance of phage-host groups (n = 25) between patients with and without bacterial infection present in at least 5/92 samples (first sample with bacterial infection *vs.* first sample of patients without bacterial infection) (Wilcoxon rank-sum test; orange dots = p <0.1; green dots = FDR <0.1). (B) Relative abundance of *Enterococcus B* phages in the first sample with bacterial infection *vs*. first sample of patients without bacterial infection (Wilcoxon rank-sum test; FDR-adjusted for 25 phage-host groups present in at least 5/92 samples). (C) 90-day survival analysis of patients with (n = 38) and without (n = 55) *Enterococcus B* phages (first sample with *Enterococcus B* phages within 90 days from first sample *vs.* first sample) (Log-rank test; FDR-adjusted for 40 phage-host groups present in at least 5/93 patients at any time point). (D) External validation cohort of patients with decompensated cirrhosis undergoing a transjugular intrahepatic portosystemic shunt. (E) Relative abundance of *Enterococcus B* phages in patients with and without proven bacterial infection in the validation cohort (Wilcoxon rank-sum test). FDR, false discovery rate.

infection and patients harboring these phages have worse short-term survival. No phage-host groups were associated with any other precipitating events (gastrointestinal bleeding, hepatic encephalopathy or severe alcohol-related hepatitis) (Tables S7–9, Fig. S11).

Validation of the association of *Enterococcus B* phages with proven bacterial infection

The association of *Enterococcus B* phages with bacterial infection was confirmed in a validation cohort of 94 patients with decompensated cirrhosis (Table 2, Fig. 6D). Short-term survival rates were too low to validate this finding. Both the relative abundance and the prevalence of *Enterococcus B* phages were higher in patients with bacterial infection (Fig. 6E; Fisher test: p = 0.05996; OR = 3.3; 8/15 *vs.* 19/75; data on bacterial infections was unavailable for four patients). In multivariable analysis, including CLIF-C AD score, ongoing antibiotic treatment and indication for TIPS, only *Enterococcus B* phages were significantly associated with the presence of proven bacterial infection (p = 0.041; Table S10). Most of the *Enterococcus B* phages in the validation cohort were also predicted to be temperate phages infecting *E. faecium*, *E. lactis* and *E. hirae* (Table S11), similar to the *Enterococcus B* phages of the MUCOSA-PREDICT cohort (Table S6). These findings highlight the potential importance of these phages, and their bacteria, in the disease course of patients with decompensated cirrhosis.

Discussion

In the present study, we analyzed the impact of the gut virome on disease progression and prognosis in a large cohort of patients with cirrhosis. We found that *Lactococcus A* phages are associated with ACLF development and that *Enterococcus B* phages are associated with bacterial infections and mortality.

Bacterial infections are the major precipitating event for ACLF, while bacterial translocation from the gut into the liver is thought to be another important trigger. The gut virome plays a pivotal role in maintaining homeostasis in the gut microbial ecosystem, thereby influencing the overall health of an individual.[46] However, its relevance in the context of decompensated cirrhosis and ACLF has not been studied in detail. In this study, we comprehensively investigated gut virome composition in a well-characterized longitudinal cohort of patients with decompensated cirrhosis and ACLF through optimized sequencing and bioinformatic methods. We demonstrated an association of *Enterococcus B* phages with proven bacterial infection and short-term mortality. In addition, we validated the association of *Enterococcus B* phages with bacterial infection in a similarly large external validation cohort.

The presence of phages targeting *Enterococcus B* bacteria, especially *E. faecium*, *E. hirae* and *E. lactis*, was shown to be associated with mortality and bacterial infection, an important precipitating event in decompensated cirrhosis.[5] *Enterococcus* phages have previously emerged as key features in the progression and development of alcohol-related hepatitis,[47,48] cirrhosis[18] and ACLF,[17] especially *E. faecalis*. Moreover, phages targeting cytolysin-positive *E. faecalis* have demonstrated a beneficial effect in alcohol-related hepatitis.[23] Interestingly, previous studies demonstrated that cytolysin itself, a toxin from *E. faecalis*, was not associated with disease progression.[49]

On the other hand, *Enterococcus* bacteria were found to be associated with disease progression in multiple studies, especially in patients with hepatitis B virus-related ACLF.[50,51] *Enterococcus B* phages may serve as a predictive biomarker of systemic bacterial infections. Their association with increased short-term mortality and bacterial infection underscore the need for adequate and timely antibiotic treatment, which is currently the only effective treatment option in patients with ACLF aside from liver transplantation.[52] Though liver transplantation represents an effective salvage therapy, several risk factors are associated with higher mortality, such as recent infection from multidrug resistant organisms.[53] In this context, phage characterization might represent an important tool enabling clinicians to select suitable antibiotics in the future. While several precipitants of ACLF have been identified,[5] there are so far no biomarkers to predict ACLF development. Progression of decompensated cirrhosis is difficult to predict, making it essential to elucidate novel factors contributing to the development of complications. Moreover, in daily clinical practice, ACLF prediction is an unmet need, which is highly relevant given its high mortality rate. Currently available tools have several limitations: Child-Pugh and MELD scores both accurately predict mortality, but not ACLF onset. Moreover, all scores lack in explaining a pathogenic relationship. Our study is the first to identify *Lactococcus A* phages as being associated with both the onset of ACLF and increased 90-day mortality, and they may serve as predictive and prognostic biomarkers. Although this result still requires validation, due to the lack of an appropriate cohort, this finding is important. In 30-40% of patients with ACLF, no precipitating event was identified in the CANONIC and PREDICT studies. These cases might be explained by microbial translocation. In this cohort,

Table 2. Overview of patient demographics and disease scores in validation cohort.

	TIPS validation cohort
Patients, n	94
Samples, n	94
Sex (Male), n (%)	49 (52.1%)
Age, mean ± SD	57.6 ± 13.2
Etiology, n (%)	
Alcohol	53 (56.4%)
Hepatitis C virus	8 (8.5%)
Alcohol + hepatitis C virus	3 (3.2%)
Other	30 (31.9%)
TIPS indication, n (%)	
Variceal bleeding	24 (25.5%)
Ascites	49 (52.1%)
Variceal bleeding + ascites	8 (8.5%)
Portal vein thrombosis	5 (5.3%)
Other	8 (8.5%)
Disease scores, mean ± SD [NAs]	
MELD score	12.5 ± 5.9 [4]
MELD-Na score	14.6 ± 6.5 [4]
Child-Pugh score	7.5 ± 1.8 [5]
CLIF-C AD score	48.4 ± 8.6 [2]

Numbers between squared brackets indicate the number of individuals for whom the variable was NA. Continuous variables are represented as mean ± standard error and categorical variables are represented as counts with percentages between brackets. AD, acute decompensation; CLIF-C, Chronic Liver Failure Consortium; MELD, model for end-stage liver disease; NA, not available; TIPS, transjugular intrahepatic portosystemic shunt.

Lactococcus A phages may play an important role. It is well described, that fecal virulent factors may be modulated.[23] *Lactococcus A* phages may change the homeostasis of the gut virome, possibly resulting in local and subsequent systemic inflammation. Ultimately, systemic inflammation may induce organ failures, which leads to development of clinically overt ACLF. Identification of patients with pre-ACLF is therefore of major importance for preventing ACLF.

On the level of individual phages and their bacterial hosts, several phage-host groups associated with clinical parameters. *Lactococcus* phages have previously been reported to be associated with alcohol use and severity in metabolic dysfunction-associated steatotic liver disease[26] and alcohol use disorder.[48] However, Jiang *et al.* reported a decrease in *Lactococcus* phages in alcohol-related hepatitis.[47]

Collectively, the existing literature and our results indicate that interactions between *Lactococcus* and *Enterococcus* bacteria and their phages are relevant in the development and progression of chronic liver disease. This is further highlighted by the association of *Enterococcus* bacteria in the blood of these same patients with spontaneous bacterial peritonitis and urinary tract infection.[45] In the same patients, *Lactobacillales* bacteria in the mucosa of the fundus significantly correlated with ACLF severity (data not shown). Whether these specific phage-bacteria interactions contribute to pathogenesis or whether they are only consequences remains a topic for further mechanistic investigations.

The relationship between increased phage diversity and disease severity, as identified in our study, is a topic of debate since changes in viral diversity in human disease are often conflicting.[36] While some studies have reported increases in viral diversity in colorectal cancer[54] and childhood obesity,[55] viral diversity has been reported to be either not associated or negatively associated with liver disease severity.[18,47,56] Bacterial diversity is negatively associated with liver disease severity,[16,17] while phage diversity increases in patients with alcohol-related hepatitis compared to alcohol use disorder,[47] while cirrhosis[18] and hepatocellular carcinoma[56] did not affect phage diversity. These discrepancies might be due to sequencing and/or bioinformatical challenges, such as a failure to account for genome fragmentation,[36] emphasizing the need for careful comparison of virome results. The increased phage diversity observed in patients with ACLF compared to those with decompensated cirrhosis, together with the decreased bacterial diversity,[17] could, for example, be attributed to the induction of integrated prophages into their extracellular state due to inflammation[44] or antibiotic usage,[57] resulting in phage-mediated bacterial lysis and consequently decreased bacterial diversity. However, many other factors might influence this complex interplay between phages and bacteria.

The highly-personalized nature of fecal viromes, together with the slow temporal drift previously observed in healthy individuals,[43,58] is extended in our study to a longitudinal cohort of patients with decompensated cirrhosis and ACLF. However, it should be noted that the inclusion of multiple samples per individual might have introduced bias, particularly affecting the significance of the patient identifier, which was identified as the most important covariate explaining beta-diversity variation. Additionally, the association between inter-individual virome variation and proteins produced by the liver, such as albumin and coagulation factors, reflects the potential effect of liver disease severity on gut virome composition. The gut virome might be indirectly influenced by liver disease severity. For example, altered bile acid metabolism might affect gut bacterial composition,[59] causing subsequent changes in virome composition. Despite the identification of several explanatory factors, a substantial fraction of inter-sample virome variation remains unexplained, as previously described.[42] Our study contributed novel insights into virome dynamics, more specifically regarding shifts in phage lifestyles observed in chronic liver disease. In other diseases, such as inflammatory bowel disease, increased levels of temperate phages have been reported.[60,61] A potential explanation for the reduced dominance of virulent phages in ACLF might be found in the high levels of systemic inflammation in ACLF,[62] as this might contribute to the induction of dormant prophages into their extracellular form.[44] The resulting phage-mediated lysis of bacteria by these induced temperate phages might subsequently enhance the inflammatory response and further contribute to intestinal inflammation.[63] Notably, our study also linked white blood cell counts, as an inflammatory marker, to phageome composition. This expansion of temperate phages highlights the dynamic nature of the gut virome during the progression of chronic liver disease, but future investigations are warranted to elucidate the mechanisms underlying this shift and its implications for liver disease progression.

Our study comprehensively analyzed longitudinal data in decompensated cirrhosis and ACLF, including the gut virome. In addition, the availability of data on the bacterial part of the microbiome allows for a more sensitive host prediction for the phages and to align the observed phage associations with their bacterial associations. However, the relatively small and heterogeneous cohort limited the statistical power. The etiology of cirrhosis is the covariate with the sixth strongest effect size on the phage variation between patients. Given the different courses of alcohol-related and viral-related liver diseases, the results should be interpreted with caution due to the heterogeneity of our cohort. Moreover, prior use of cephalosporins before inclusion in the study, which could not be accounted for in our analysis, may represent a potential source of bias since its use is recommended by clinical guidelines. Despite these limitations, we were able to validate the association of *Enterococcus B* phages with bacterial infection in patients with decompensated cirrhosis. Due to the scarcity of gut virome studies in patients with decompensated cirrhosis and especially ACLF, no suitable cohorts were available to validate the other findings. Of note, the validation cohort also comprised more patients with an alcohol-related etiology and showed higher MELD and CLIF-C AD scores. Further validation cohorts, also including patients with compensated cirrhosis, are warranted in future studies, to better encompass the natural history of disease and to analyze differences in the gut virome in earlier stages of chronic liver disease. Differences in data analysis, especially regarding phage identification and host prediction methods, further complicate the comparison of our findings with existing literature.

In conclusion, our study represents a first effort in unraveling the role of the gut virome in decompensated cirrhosis and ACLF. We have identified significant changes in virome composition and found associations of phage-host groups with progression and outcome. The study underscores the

complexity of the gut virome in human health and disease, and highlights the need for a deeper understanding of virome-bacteriome-host interactions to pave the way for novel clinical applications in the field of hepatology.

Affiliations

[1]*KU Leuven, Department of Microbiology, Immunology, & Transplantation, Rega Institute, Division of Clinical & Epidemiological Virology, Laboratory of Viral Metagenomics, Belgium;* [2]*Department of Internal Medicine B, University of Münster, Münster, Germany;* [3]*Structural and Computational Biology Unit, European Molecular Biology Laboratory, Heidelberg, Germany;* [4]*Division of Gastroenterology, Department of Internal Medicine, Faculty of Medicine, University of Debrecen, Debrecen, Hungary;* [5]*Department of Medicine, University of California San Diego, La Jolla, CA, USA;* [6]*Max Delbrück Centre for Molecular Medicine, Berlin, Germany;* [7]*Department of Bioinformatics, Biocenter, University of Würzburg, Würzburg, Germany;* [8]*Department of Gastroenterology & Hepatology, Section of Liver & Biliopancreatic disorders and Liver Transplantation, University Hospitals Leuven, KU Leuven, Leuven, Belgium;* [9]*Division of Gastroenterology, Hepatology, and Nutrition, Virginia Commonwealth University and Richmond VA Medical Center, Richmond, USA;* [10]*Department of Clinical Research, Faculty of Health Sciences, University of Southern Denmark, Odense, Denmark;* [11]*European Foundation for the Study of Chronic Liver Failure, Barcelona, Spain*

Abbreviations

ACLF, acute-on-chronic liver failure; AD, acute decompensation; CLIF-C, Chronic Liver Failure Consortium; dbRDA, distance-based redundancy analysis; DC = decompensated cirrhosis; EASL-CLIF, European Association for the Study of the Liver – Chronic Liver Failure; MELD, model for end-stage liver disease; TIPS, transjugular intrahepatic portosystemic shunt.

Financial support

The MICROB-PREDICT project has received funding from the European Union's Horizon 2020 research and innovation programme (grant agreement: No 825694). Project 138041 was funded by the Ministry of Innovation and Technology, Hungary, with support from the National Research Development and Innovation Fund, Hungary, under the K grant scheme. The TIPS study was supported by the Novo Nordisk Foundation (Challenge Grant "MicrobLiver": NNF15OC0016692). LVE was supported by the Research Foundation Flanders (FWO: 1S25720N). MJB is supported by the Deutsche Forschungsgemeinschaft (DFG, German Research Foundation) – project ID 493624047 (Clinician Scientist CareerS Münster). JT was supported by the German Research Foundation (DFG) project ID 403224013 – SFB 1382 (A09), by the German Federal Ministry of Education and Research (BMBF) for the DEEP-HCC project and by the Hessian Ministry of Higher Education, Research and the Arts (HMWK) for the ACLF-I cluster project. The DECISION (project ID 847949), GALAXY (project ID 668031), LIVERHOPE (project ID 731875), and IHMCSA (project ID 964590) projects have received funding from the European Union's Horizon 2020 research and innovation program. TN is supported by JSPS KAKENHI (Grant No. 23KJ2059). BS is supported by NIDDK grant P30 DK120515. The computational resources were provided by the Flemish Supercomputer Center (VSC) and funded by FWO and the Flemish Government Department of Economy, Science, and Innovation. The funders had no role in study design, data collection and interpretation, or the decision to submit the work for publication.

Conflict of interest

JT has received speaking and/or consulting fees from Versantis, Gore, Boehringer-Ingelheim, Falk, Grifols, Genfit and CSL Behring. The other authors have reported no conflict of interest related to this work.

Please refer to the accompanying ICMJE disclosure forms for further details.

Authors' contributions

Study concept and design; JT, JM, MP, PB, WL; Acquisition of data; MP, BB, LVE, LC, WG; Analysis and interpretation of data; LVE, RS, MJB, LDC, MIK, TN, AF, MK, JT, JM, JSB, BS; Drafting of the manuscript; LVE, MJB, RS, MIK, MK, JT, JM; Critical revision of the manuscript for important intellectual content; all authors; Statistical analysis; LVE, LDC, MK; Obtained funding; JT, MP, PB, JM, WL; Administrative, technical, or material support; LC, WG, RS, AF; Study supervision: JT, JM.

Data availability

Sequencing data are publicly available at the European Nucleotide Archives (ENA). Their accession numbers are listed in Supplementary Table 12. Any additional information required to reanalyze the data reported in this paper is available from the corresponding author upon request.

Supplementary data

Supplementary data to this article can be found online at https://doi.org/10.1016/j.jhepr.2025.101622.

References

Author names in bold designate shared co-first authorship

[1] Moreau R, Jalan R, Gines P, et al. Acute-on-Chronic liver failure is a distinct syndrome that develops in patients with acute decompensation of cirrhosis. Gastroenterology 2013;144:1426–1437.e9.

[2] GBD 2013 DALYs and HALE Collaborators, Murray CJL, Barber RM, et al. Global, regional, and national disability-adjusted life years (DALYs) for 306 diseases and injuries and healthy life expectancy (HALE) for 188 countries, 1990-2013: quantifying the epidemiological transition. Lancet Lond Engl 2015;386:2145–2191.

[3] Ferstl P, Trebicka J. Acute decompensation and acute-on-chronic liver failure. Clin Liver Dis 2021;25:419–430.

[4] Trebicka J, Fernandez J, Papp M, et al. The PREDICT study uncovers three clinical courses of acutely decompensated cirrhosis that have distinct pathophysiology. J Hepatol 2020;73:842–854.

[5] **Trebicka J**, **Fernandez J**, Papp M, et al. PREDICT identifies precipitating events associated with the clinical course of acutely decompensated cirrhosis. J Hepatol 2021 May;74(5):1097–1108.

[6] Trebicka J, Bork P, Krag A, et al. Utilizing the gut microbiome in decompensated cirrhosis and acute-on-chronic liver failure. Nat Rev Gastroenterol Hepatol 2020:1–14.

[7] Chen Y, Zhou J, Wang L. Role and mechanism of gut microbiota in human disease. Front Cell Infect Microbiol 2021;11.

[8] Chevallereau A, Pons BJ, Houte S van, et al. Interactions between bacterial and phage communities in natural environments. Nat Rev Microbiol 2022;20:49–62.

[9] Borodovich T, Shkoporov AN, Ross RP, et al. Phage-mediated horizontal gene transfer and its implications for the human gut microbiome. Gastroenterol Rep 2022;10(goac012).

[10] Van Belleghem JD, Dąbrowska K, Vaneechoutte M, et al. Interactions between bacteriophage, bacteria, and the mammalian immune system. Viruses 2019;11:10.

[11] **Bichet MC**, **Adderley J**, Avellaneda-Franco L, et al. Mammalian cells internalize bacteriophages and use them as a resource to enhance cellular growth and survival. Plos Biol 2023;21:e3002341.

[12] **Guo J**, **Bolduc B**, Zayed AA, et al. VirSorter2: a multi-classifier, expert-guided approach to detect diverse DNA and RNA viruses. Microbiome 2021:1–13.

[13] **Camargo AP**, **Roux S**, Schulz F, et al. Identification of mobile genetic elements with geNomad. Nat Biotechnol 2024 Aug;42(8):1303–1312.

[14] Coutinho FH, Zaragoza-Solas A, López-Pérez M, et al. RaFAH: host prediction for viruses of Bacteria and Archaea based on protein content. Patterns 2021;2:100274.

[15] **Roux S**, **Camargo AP**, Coutinho FH, et al. iPHoP: an integrated machine learning framework to maximize host prediction for metagenome-derived viruses of archaea and bacteria. PLOS Biol 2023;21:e3002083.

[16] Qin N, Yang F, Li A, et al. Alterations of the human gut microbiome in liver cirrhosis. Nature 2014;513:59–64.

[17] **Solé C**, **Guilly S**, Silva KD, et al. Alterations in gut microbiome in cirrhosis as assessed by quantitative metagenomics: relationship with acute-on-chronic liver failure and prognosis. Gastroenterology 2021;160:206–218.e13.

[18] Bajaj JS, Sikaroodi M, Shamsaddini A, et al. Interaction of bacterial metagenome and virome in patients with cirrhosis and hepatic encephalopathy. Hepatology 2020;5.

[19] **Lang S**, **Demir M**, Martin A, et al. Intestinal virome signature associated with severity of nonalcoholic fatty liver disease. Gastroenterology 2020;159:1839–1852.

[20] Jinato T, Sikaroodi M, Fagan A, et al. Alterations in gut virome are associated with cognitive function and minimal hepatic encephalopathy cross-sectionally and longitudinally in cirrhosis. Gut Microbes 2023;15: 2288168.
[21] **Rodríguez MP**, **Fagan A**, Sikaroodi M, et al. Proton pump inhibitor use and complications of cirrhosis are linked with distinct gut microbial bacteriophage and eukaryotic viral like particle signatures in cirrhosis. Clin. Transl. Gastroenterol.; 2023.
[22] Bajaj JS, Peña-Rodriguez M, La Reau A, et al. Longitudinal transkingdom gut microbial approach towards decompensation in outpatients with cirrhosis. Gut 2023;72:759–771.
[23] **Duan Y**, **Llorente C**, Lang S, et al. Bacteriophage targeting of gut bacterium attenuates alcoholic liver disease. Nature 2019 Nov;575(7783):505–511.
[24] Gan L, Feng Y, Du B, et al. Bacteriophage targeting microbiota alleviates non-alcoholic fatty liver disease induced by high alcohol-producing Klebsiella pneumoniae. Nat Commun 2023;14:3215.
[25] Yuan J, Chen C, Cui J, et al. Fatty liver disease caused by high-alcohol-producing Klebsiella pneumoniae. Cell Metab 2019;30:675–688.e7.
[26] Hsu CL, Duan Y, Fouts DE, et al. Intestinal virome and therapeutic potential of bacteriophages in liver disease. J Hepatol 2021;75:1465–1475.
[27] Angeli P, Bernardi M, Villanueva C, et al. EASL Clinical Practice Guidelines for the management of patients with decompensated cirrhosis. J Hepatol 2018;69:406–460.
[28] Lehmann JM, Claus K, Jansen C, et al. Circulating CXCL10 in cirrhotic portal hypertension might reflect systemic inflammation and predict ACLF and mortality. Liver Int 2018;38:875–884.
[29] **Blaya D**, **Pose E**, Coll M, et al. Profiling circulating microRNAs in patients with cirrhosis and acute-on-chronic liver failure. JHEP Rep 2021;3.
[30] **Wirtz TH**, **Reuken PA**, Jansen C, et al. Balance between macrophage migration inhibitory factor and sCD74 predicts outcome in patients with acute decompensation of cirrhosis. JHEP Rep 2021;3.
[31] Conceição-Neto N, Zeller M, Lefrère H, et al. Modular approach to customise sample preparation procedures for viral metagenomics: a reproducible protocol for virome analysis. Sci Rep 2015 Nov 12;5:16532.
[32] Buchfink B, Xie C, Huson DH. Fast and sensitive protein alignment using DIAMOND. Nat Methods 2014;12:59–60.
[33] Camacho C, Coulouris G, Avagyan V, et al. BLAST+: architecture and applications. BMC Bioinformatics 2009;10:421.
[34] **Nayfach S**, **Camargo AP**, Schulz F, et al. CheckV assesses the quality and completeness of metagenome-assembled viral genomes. Nat Biotechnol 2020:1–8.
[35] **Almeida A**, **Nayfach S**, Boland M, et al. A unified catalog of 204,938 reference genomes from the human gut microbiome. Nat Biotechnol 2021 Jan;39(1):105–114.
[36] Jansen D, Matthijnssens J. The emerging role of the gut virome in health and inflammatory bowel disease: challenges, covariates and a viral imbalance. Viruses 2023;15:173.
[37] Benjamini Y, Hochberg Y. Controlling the false discovery rate: a practical and powerful approach to multiple testing. J R Stat Soc Ser B Methodol 1995;57:289–300.
[38] Oksanen J, Blanchet FG, Friendly M, et al. Package “vegan”: community ecology package version 2.5-7. 2020.
[39] Manrique P, Bolduc B, Walk ST, et al. Healthy human gut phageome. Proc Natl Acad Sci 2016;113:10400–10405.
[40] Arumugam M, Raes J, Pelletier E, et al. Enterotypes of the human gut microbiome. Nature 2011;473:174–180.
[41] **Yan M**, **Pratama AA**, Somasundaram S, et al. Interrogating the viral dark matter of the rumen ecosystem with a global virome database. Nat Commun 2023;14:5254.
[42] Nishijima S, Nagata N, Kiguchi Y, et al. Extensive gut virome variation and its associations with host and environmental factors in a population-level cohort. Nat Commun 2022;13:5252.
[43] **Shkoporov AN**, **Clooney AG**, Sutton TDS, et al. The human gut virome is highly diverse, stable, and individual specific. Cell Host & Microbe 2019;26:527–541.
[44] **Diard M**, **Bakkeren E**, Cornuault JK, et al. Inflammation boosts bacteriophage transfer between *Salmonella* spp. Science 2017;355:1211–1215.
[45] Bajaj JS, Rodriguez MP, Fagan A, et al. Impact of bacterial infections and spontaneous bacterial peritonitis prophylaxis on phage-bacterial dynamics in cirrhosis. Hepatology 2022;76:1723.
[46] Tiamani K, Luo S, Schulz S, et al. The role of virome in the gastrointestinal tract and beyond. FEMS Microbiol Rev 2022;46(fuac027).
[47] **Jiang L**, **Lang S**, Duan Y, et al. Intestinal virome in patients with alcoholic hepatitis. Hepatol Baltim Md 2020;72:2182–2196.
[48] Hsu CL, Zhang X, Jiang L, et al. Intestinal virome in patients with alcohol use disorder and after abstinence. Hepatol Commun 2022;00:1–12.
[49] Hartmann P, Lang S, Schierwagen R, et al. Fecal cytolysin does not predict disease severity in acutely decompensated cirrhosis and acute-on-chronic liver failure. Hepatobiliary Pancreat Dis Int HBPD INT 2023;22:474–481.
[50] Wang K, Zhang Z, Mo Z-S, et al. Gut microbiota as prognosis markers for patients with HBV-related acute-on-chronic liver failure. Gut Microbes 2021;13:1–15.
[51] Yao X, Yu H, Fan G, et al. Impact of the gut microbiome on the progression of hepatitis B virus related acute-on-chronic liver failure. Front Cell Infect Microbiol 2021;11:573923.
[52] European Association for the Study of the Liver. Electronic address: easloffice@easloffice.eu, and European Association for the Study of the Liver. EASL Clinical Practice Guidelines on acute-on-chronic liver failure. J Hepatol 2023;79:461–491.
[53] Belli LS, Duvoux C, Artzner T, et al. Liver transplantation for patients with acute-on-chronic liver failure (ACLF) in Europe: results of the ELITA/EF-CLIF collaborative study (ECLIS). J Hepatol 2021;75:610–622.
[54] **Nakatsu G**, **Zhou H**, Wu WKK, et al. Alterations in enteric virome are associated with colorectal cancer and survival outcomes. Gastroenterology 2018;155:529–541.e5.
[55] **Bikel S**, **López-Leal G**, Cornejo-Granados F, et al. Gut dsDNA virome shows diversity and richness alterations associated with childhood obesity and metabolic syndrome. iScience 2021;24.
[56] Zhang F, Gia A, Chen G, et al. Critical assessment of whole genome and viral enrichment shotgun metagenome on the characterization of stool total virome in hepatocellular carcinoma patients. Viruses 2023;15:53.
[57] Sutcliffe SG, Maurice CF. Not just a passing phage. Cell Host Microbe 2019;26:448–449.
[58] **Van Espen L**, **Bak EG**, Beller L, et al. A previously undescribed highly prevalent phage identified in a Danish enteric virome catalog. mSystems 2021;6:e00382. 21.
[59] Zhu F, Zheng S, Zhao M, et al. The regulatory role of bile acid microbiota in the progression of liver cirrhosis. Front Pharmacol 2023;14.
[60] Clooney AG, Sutton TDS, Shkoporov AN, et al. Whole-virome analysis sheds light on viral dark matter in inflammatory bowel disease. Cell Host Microbe 2019 Dec 11;26(6):764–778.e5.
[61] **Jansen D**, **Falony G**, Vieira-Silva S, et al. Community types of the human gut virome are associated with endoscopic outcome in ulcerative colitis. J Crohns Colitis 2023;17:1504–1513.
[62] Laleman W, Claria J, Van Der Merwe S, et al. Systemic inflammation and acute-on-chronic liver failure: too much, not enough. Can J Gastroenterol Hepatol 2018;2018:1–10.
[63] Zuppi M, Hendrickson HL, O’Sullivan JM, et al. Phages in the gut ecosystem. Front Cell Infect Microbiol 2022;11.

Keywords: acutely decompensated cirrhosis; acute-on-chronic liver failure; fecal virome; fecal microbiome; gut phages; phage host prediction.

Received 3 April 2025; received in revised form 23 September 2025; accepted 29 September 2025; Available online 10 October 2025

Journal of Hepatology, Volume *8*

Supplemental information

Lactococcus A phages predict ACLF while *Enterococcus B* phages predict bacterial infection in decompensated cirrhosis

Lore Van Espen, Maximilian Joseph Brol, Lila Close, Robert Schierwagen, Wenyi Gu, Marisa I. Keller, Boglarka Balogh, Anthony Fullam, Lander De Coninck, Tomohiro Nakamura, Michael Kuhn, Peer Bork, Wim Laleman, Jasmohan S. Bajaj, Maria Papp, Bernd Schnabl, Jonel Trebicka, Jelle Matthijnssens, and on behalf of MICROB-PREDICT

Enterococcus B and *Lactococcus A* phages are associated with increased mortality in patients with decompensated cirrhosis and ACLF

Lore Van Espen, Maximilian Joseph Brol, Lila Close, Robert Schierwagen, Wenyi Gu, Marisa I Keller, Boglarka Balogh, Anthony Fullam, Lander de Coninck, Tomohiro Nakamura, Michael Kuhn, Peer Bork, Wim Laleman, Jasmohan S Bajaj, Maria Papp, Bernd Schnabl, Jonel Trebicka, Jelle Matthijssens

Table of contents

Supplementary Methods 3
Fig. S1 8
Fig. S2 10
Fig. S3 11
Fig. S4 11
Fig. S5 12
Fig. S6 12
Fig. S7 13
Fig. S8 14
Fig. S9 14
Fig. S10 15
Fig. S11 16
Table S1 17
Table S2 18
Table S3 18
Table S4 19
Table S5 20
Table S6 20
Table S7 21
Table S8 21
Table S9 22
Table S10 22
Table S11 23

Table S12 23
Supplementary References 30

Supplementary methods

Sample preparation for virome sequencing

Viral-like particles were purified from fecal samples and prepared for virome sequencing using the NetoVIR protocol[1]. In short, fecal aliquots were homogenized in PBS (10 m/V%) using a Precellys homogenizer (Bertin technologies, 5,000 rpm for 15 s), centrifuged (17,000 g for 3 min), filtered (0.8 µm PES filter, Sartorius) and treated with a mixture of benzonase (Novogen) and micrococcal nuclease (New England Biolabs) to enrich for viral-like particles. Both RNA and DNA were extracted using the Viral RNA Mini kit (Qiagen) without carrier RNA. Extracts were reverse transcribed and randomly amplified (17 cycles) using a modified WTA2 kit (Sigma Aldrich) and purified using the MSB Spin PCRapace purification kit (Stratec). Sequencing libraries were prepared with the Nextera XT DNA Library Preparation kit (Illumina), quantified with the Qubit dsDNA High Sensitivity kit (Thermo Fisher Scientific) and the insert length was determined using the Bioanalyzer2000 and the High Sensitivity DNA kit (Agilent). Libraries were sequenced (2 x 150 bp) on the NextSeq500 Illumina platform (Nucleomics Core facility, KU Leuven, Belgium). Seven samples were not sequenced due to too low concentrations after random amplification or library preparation.

Quality control and *de novo* assembly of virome dataset

Raw reads were trimmed using Trimmomatic (v0.36)[2] to remove primers/adaptors (parameters: 30:10:1:true) and remove bad-quality (parts of) reads (parameters: HEADCROP:19 LEADING:15 TRAILING:15 SLIDINGWINDOW:4:20 MINLEN:50). Quality filtered reads were *de novo* assembled using metaSPAdes (v3.11.1, kmer sizes of 21, 33, 55 and 77)[3]. To overcome the issue of highly covered but fragmented genomes, the *de novo* assembly was performed on subsets of 100%, 10% and 1% of the quality-filtered reads generated by seqkit (v0.10.0)[4]. Per sample, the resulting scaffolds > 1 kb were clustered to remove redundancy across all three assemblies at 99% ANI over 99% coverage using BLASTn (v2.5.0+)[5] and the anicalc.py and aniclust.py scripts as described on the CheckV github (v0.7.0)[6]. Afterwards, all remaining scaffolds were clustered across samples at 95% ANI over 85% coverage to remove cross-sample redundancy.

Identification of viral scaffolds

Human scaffolds were identified by Kraken2 (v2.1.1, database downloaded on 2021/05/20, parameters: --confidence 0.5)[7]. The non-redundant scaffold set was compared against the NCBI nucleotide dataset (version of 2021/05/14) and protein dataset (downloaded on 2021/05/20) using BLASTn (v2.10.0+, parameters: -evalue 1e-10)[5] and DIAMOND (v2.0.9, parameters: --sensitive)[8], respectively. The lowest common ancestor (LCA) of the top nucleotide and protein hits were determined using ktClassifyBLAST from KronaTools (v2.7.1, database downloaded on 2021/05/20)[9]. CheckV (v0.8.1, database v1.0)[6] was used to determine completeness of viral genomes. Scaffolds were classified as eukaryotic viruses if the DIAMOND LCA was a eukaryotic virus. If the DIAMOND LCA was "Root" or if the CheckV completeness estimate was ≥ 50% and the DIAMOND LCA was no virus, the BLASTn LCA was used for annotation instead (if this was a eukaryotic virus). Phages were identified by VirSorter2 (v2.2.2, database downloaded on 2021/02/03)[10] including the ssDNA, dsDNAphage and RNA viral groups (no score cut-off) and required a completeness estimate by CheckV (i.e. not "Not-determined") to be selected. Members of the *Inoviridae* phage family were selected based on DIAMOND LCA as both VirSorter2 and CheckV are less suited to detect this group of viruses well. The analysis of the phageome will focus on the "good-quality" phages (i.e. estimated to be ≥ 50% complete by CheckV[6] or length ≥ 4 kb for phages of the *Inoviridae* family) (4% of phage scaffolds; 79.4% phage reads; Fig. 1B) to reduce the noise resulting from highly fragmented genomes.

Abundance determination for virome dataset

Instead of determining abundances by mapping quality-filtered reads per sample to the entire non-redundant scaffold set, reads were only mapped against the representatives of the clusters containing a scaffold from that sample, to avoid false positive detection of closely related sequences, using BWA-MEM2 (v2.2.1)[11]. A scaffold was assumed to be present if 70% of its length was horizontally covered by reads as determined by samtools depth (v1.7)[12]. Decontam (v1.10.0)[13] was used to identify contaminating scaffolds in the extraction and amplification controls separately, using the prevalence mode (0.5 score threshold) which relies on the assumption that contamination has a higher prevalence in controls than in samples. Afterwards, remaining non-contaminating viral reads were random subsampled to a depth of

329,569 viral reads using the "rarefy_even_depth" function from Phyloseq (v1.34)[14], removing ten samples from subsequent analyses with less viral reads.

Taxonomical classification of viral scaffolds

Since phage taxonomy is a rapidly evolving field and the large majority of phage sequences are unclassified to begin with, "good-quality" phages are clustered into groups that roughly represent genus and family levels based on gene sharing (minimal 20% or 16 genes and 10% or 8 genes, respectively) and average amino-acid identity (minimal 40 and 20%, respectively) as described previously [15]. Open-reading frames used to perform this clustering were predicted using Prodigal (v2.6.3)[16] in metagenomic mode. A taxonomic group was assigned to clusters including RefSeq phage genomes (v209; n = 4,733)[17] (i.e. crassphages, non-crass caudoviruses, inoviruses and microviruses).

Processing of fecal bacteriome dataset

DNA was extracted from stool samples using Qiagen AllPrep Power Fecal DNA/RNA Kit. Metagenomic sequencing libraries were prepared using the NEBNext Ultra II DNA Library Prep kit with a targeted insert size of 350 – 400 bp and Dual Index multiplex oligos. Libraries were prepared using liquid automated systems (Beckman Coulter i7 Series) and sequenced on an Illumina HiSeq 4000 platform (Illumina, San Diego, CA, USA) with 2 x 150 bp paired-end reads. Metagenomic data was processed to remove low quality data using NGLess (v1.1)[18]. Nucleotide calls with a Phred score of less than 25 were removed from the 3' end. Any reads that were less than 45 nucleotides long after removal of low-quality nucleotide calls were discarded. Any reads with greater than 90 % sequence similarity to the human reference genome (hg38) were discarded. Reads were re-classified as paired or as singles, where, respectively, both or only the forward and reverse reads are present in the final dataset.

Phage host and lifestyle prediction

Good-quality phages were linked to putative hosts using a combination of methods relying on two host databases. The first host database contains the metagenome-assembled genomes (MAGs) from whole-genome shotgun metagenomic sequences of the same fecal samples (n = 15,543). Assembly of quality-controlled metagenomic reads was performed using megahit (v1.2.9)[19] followed by binning using metabat (v2.12.1)[20]. The second host database contains bacterial species representatives (n =

4,744) from the Unified Human Gastrointestinal Genomes (UHGG) database (v2.0)[21]. Both databases have been taxonomically annotated using the GTDB taxonomy (v202) using GTDB_Tk (v1.5.0)[22].

Each phage – host genus pair was scored based on prophage matches, CRISPR spacer matches, tRNA matches, PHIST[23] and RaFAH[24] and every pair with a score of three of more (see below) was considered a putative host genus for that phage.

(I) Prophage matches: Phage genomes were compared to both MAG databases using BLASTn (v2.0.5+, parameters: -perc_identity 90)[5]. ANI, query coverage (qcov) and target coverage (tcov) between phage and MAG contig were calculated using the anicalc.py script from the CheckV github[6] and hits ≤ 90% ANI or > 70% tcov were removed (to require at least 30% of the host genome outside of the prophage region to avoid hits to misbinned phage genomes in the MAGs as described in Gregory & Zablocki, 2022[25]). A phage – host genus pair with one prophage match with > 30% qcov received two points, while two matches with > 30% qcov or one match with > 70% qcov received three points.

(II & III) CRISPR & tRNA matches: CRISPR spacers and tRNAs were predicted on both MAG datasets using respectively MinCED (v0.4.2)[26] and Aragorn (v1.2.41)[27]. CRISPR spacers were compared against the phages using BLASTn (v2.0.5+, parameters: --task blastn-short)[5] and tRNAs against the tRNAs predicted on the good-quality phages by Aragorn (v1.2.41)[27] using BLASTn (v2.0.5+)[5]. Only hits without gaps and spanning at least 95% of the CRISPR spacer or 97% of the tRNA gene were considered. A phage – host genus pair with a CRISPR or tRNA match with maximum 1 mismatch received one point, while a CRISPR or tRNA match without mismatch resulted in two points for the CRISPR and tRNA scores respectively.

(IV) PHIST: PHIST (v1.0.0)[23] was run on the good-quality phage contigs and both MAG datasets. A phage – host genus pair with a PHIST adj. $p < 0.001$ received one point, while a PHIST adj. $p < 0.000001$ resulted in two points.

(V) RaFAH: RaFAH (v0.3)[24] was run on the good-quality phage contigs (no need for MAG input). The RaFAH genera were converted to GTDB genera to align with the MAG taxonomical classification and every phage – host genus with a RaFAH score > 0.1 received one point, pairs with a RaFAH score > 0.5 received two points and pairs with a RaFAH score > 0.8 received three points.

The lifestyle of the good-quality phages was predicted using BACPHLIP (v0.9.6)[28]. A phage with a BACPHLIP temperate score > 0.5 or a prophage match to a MAG from either datasets (> 90% ANI, ≤ 70% tcov and > 30% qcov) was classified as temperate. Additionally, phages part of predominantly temperate phages with an estimated completeness > 90% will be classified as temperate phages. All other good-quality phages were classified as virulent.

Analysis of fecal bacteriome dataset

Metagenomes were analyzed using mOTUs (v2.5)[29], which requires the identification of at least three marker genes for reliable taxonomic profiling, adhering to NCBI taxonomy. mOTU taxonomical classifications were transformed into GTDB taxonomy to align with the bacterial taxonomy used for phage host prediction. We used GTDB-tk on ProGenomes2 database genomes to assign taxonomy for marker genes defining mOTUs[22,30]. Out of 11,699 clusters, 750 displayed taxonomic inconsistencies at the species level. We set a threshold of 80% consistency for taxonomic assignments within conflict clusters and excluded unknown results. For unresolved cases, we noted multiple genera to mark ambiguous taxonomy.

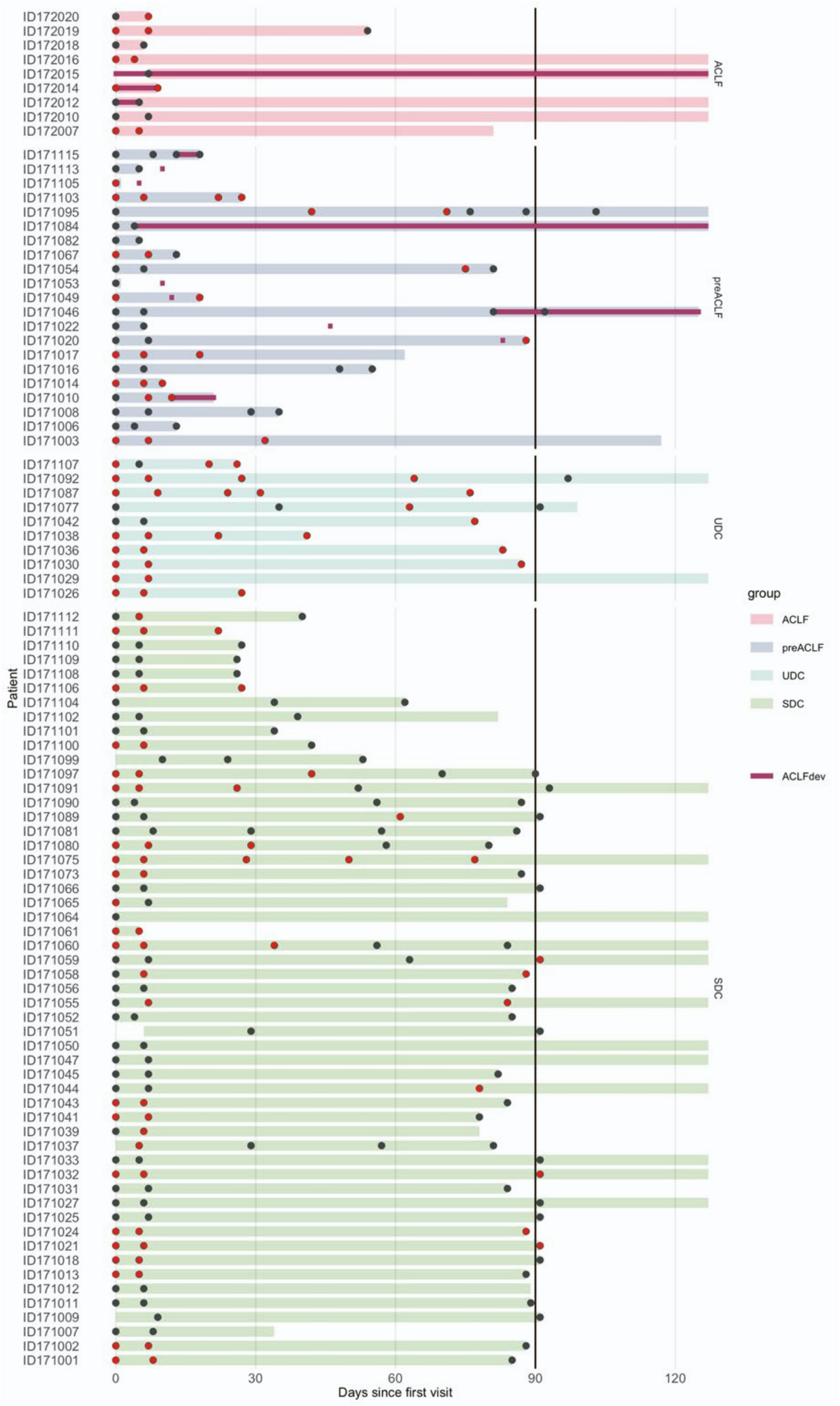

ID172020
ID172019
ID172018
ID172016
ID172015
ID172014
ID172012
ID172010
ID172007
ID171115
ID171113
ID171105
ID171103
ID171095
ID171084
ID171082
ID171067
ID171054
ID171053
ID171049
ID171046
ID171022
ID171020
ID171017
ID171016
ID171014
ID171010
ID171008
ID171006
ID171003
ID171107
ID171092
ID171087
ID171077
ID171042
ID171038
ID171036
ID171030
ID171029
ID171026
ID171112
ID171111
ID171110
ID171109
ID171108
ID171106
ID171104
ID171102
ID171101
ID171100
ID171099
ID171097
ID171091
ID171090
ID171089
ID171081
ID171080
ID171075
ID171073
ID171066
ID171065
ID171064
ID171061
ID171060
ID171059
ID171058
ID171056
ID171055
ID171052
ID171051
ID171050
ID171047
ID171045
ID171044
ID171043
ID171041
ID171039
ID171037
ID171033
ID171032
ID171031
ID171027
ID171025
ID171024
ID171021
ID171018
ID171013
ID171012
ID171011
ID171009
ID171007
ID171002
ID171001
Patient
ACLF
preACLF
UDC
SDC
0
30
60
90
120
Days since first visit
group
ACLF
preACLF
UDC
SDC
ACLFdev

Fig. S1: Overview of longitudinal MUCOSA-PREDICT cohort.

Lines represent the follow-up period of all patients colored by disease group. Dots indicate visits included in virome analysis (visits after 120 days (n = 11) are excluded from this figure for clarity). Purple indicates the presence of ACLF in these patients. Red dots indicate presence of bacterial infection at visit. Black line represents the 90-day follow-up period used to stratify patients with AD into pre-ACLF, unstable and stable decompensated cirrhosis groups. ACLF = acute-on-chronic liver failure; AD = acute decompensation; SDC = stable decompensated cirrhosis; UDC = unstable decompensated cirrhosis.

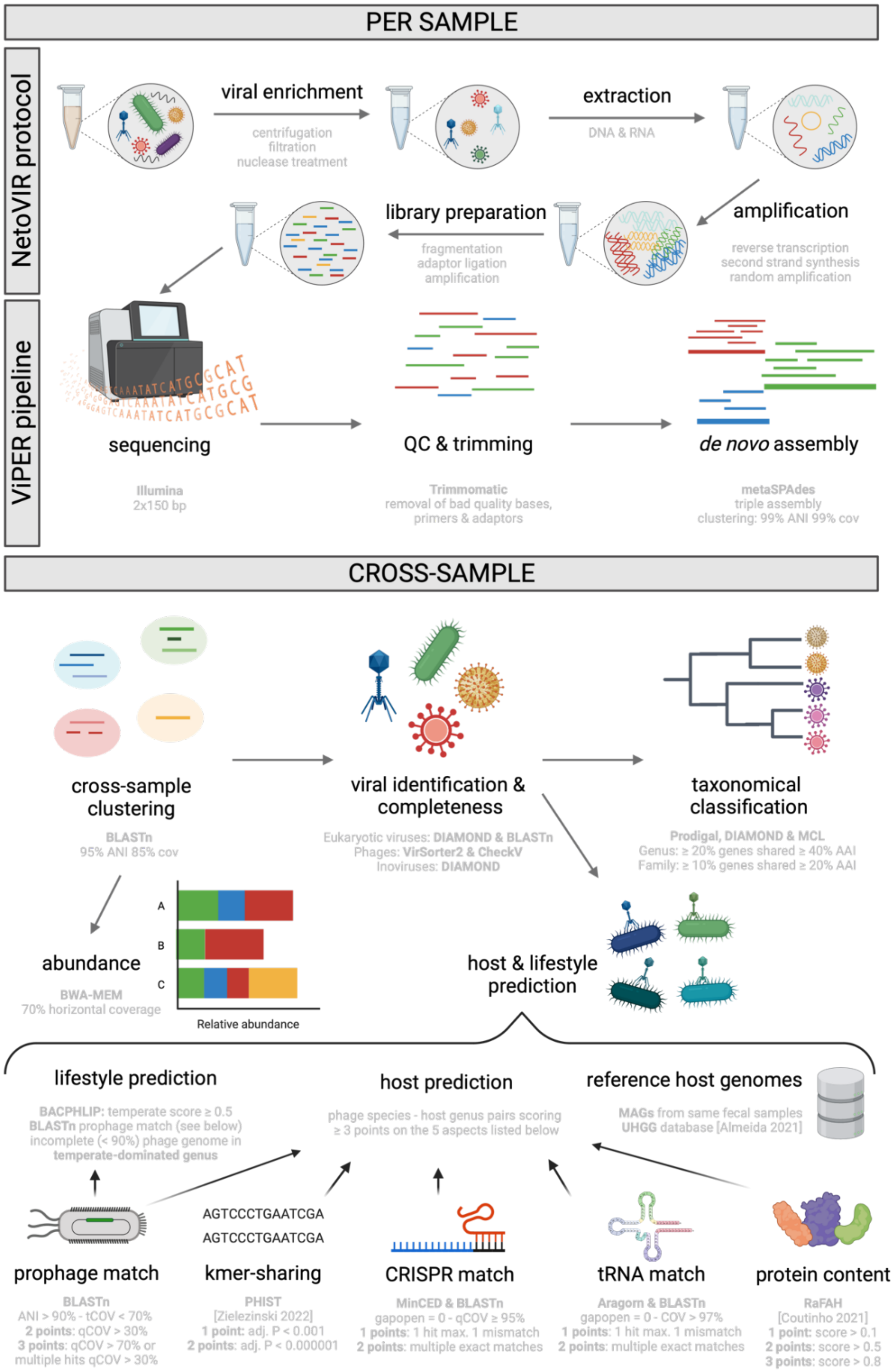

Fig. S2: Overview of fecal sample preparation and bioinformatic processing for virome profiling.

Overview of different steps in the sample preparation, sequencing and bioinformatic processing of the fecal samples for virome profiling. Created with BioRender.

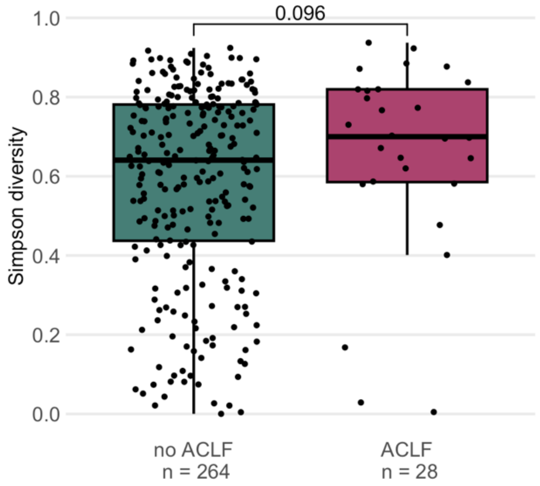

Fig. S3: Phage Simpson diversity associated with ACLF.

Comparison of phage alpha-diversity in samples with and without ACLF (Wilcoxon rank-sum test). ACLF = acute-on-chronic liver failure.

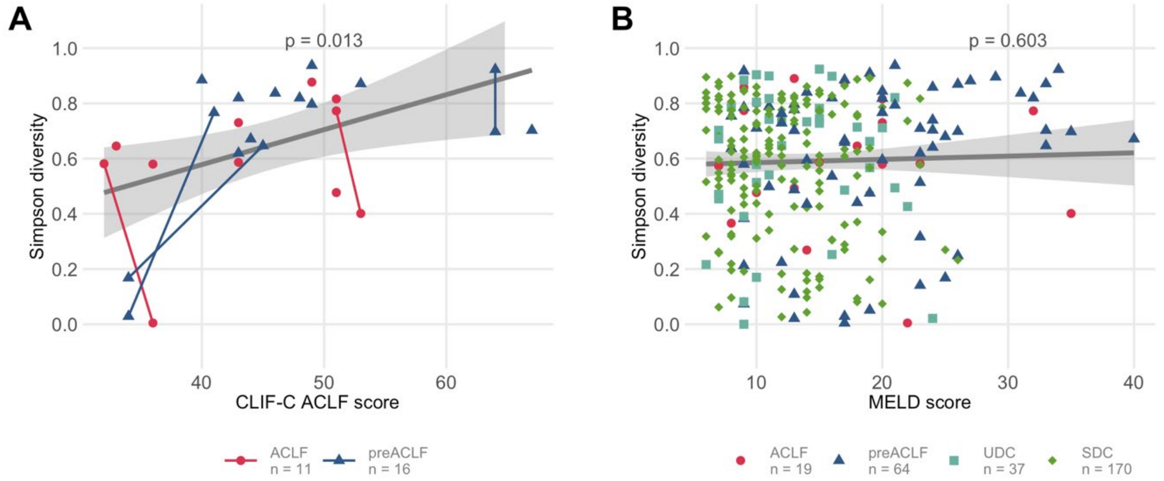

Fig. S4: Phage Simpson diversity associated with CLIF-C ACLF, but not MELD score

(A) Relationship between phage alpha-diversity and CLIF-C ACLF score across all ACLF visits of all (pre)ACLF patients (Pearson's correlation coefficient = 0.47). Samples from the same patient are connected. (B) Relationship between phage alpha-diversity and MELD score across all visits of all patients (Pearson's correlation coefficient = 0.03 (ns)). ACLF = acute-on-chronic liver failure; UDC = unstable decompensated cirrhosis; SDC = stable decompensated cirrhosis.

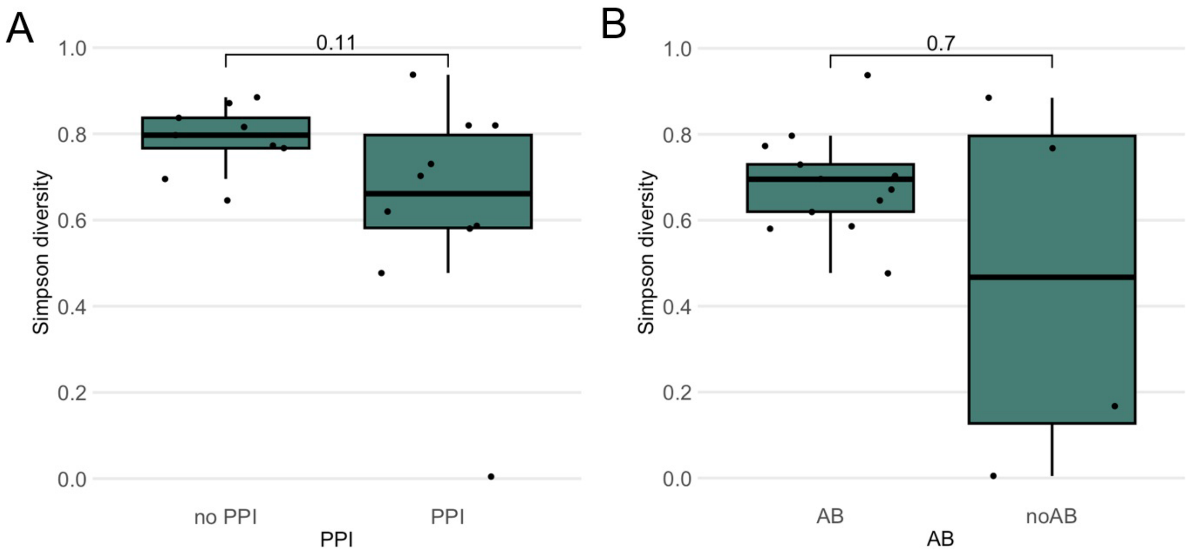

Fig. S5: Phage Simpson diversity in relation to proton pump inhibitor and antibiotic use. (A) Comparison of phage alpha-diversity in samples from patients with ACLF with or without concurrent use of PPI (Wilcoxon rank-sum test). (B) Comparison of phage alpha-diversity in samples from patients with ACLF with or without concurrent use of antibiotics (Wilcoxon rank-sum test). AB = antibiotics; PPI = proton pump inhibitors; ACLF = acute-on-chronic liver failure.

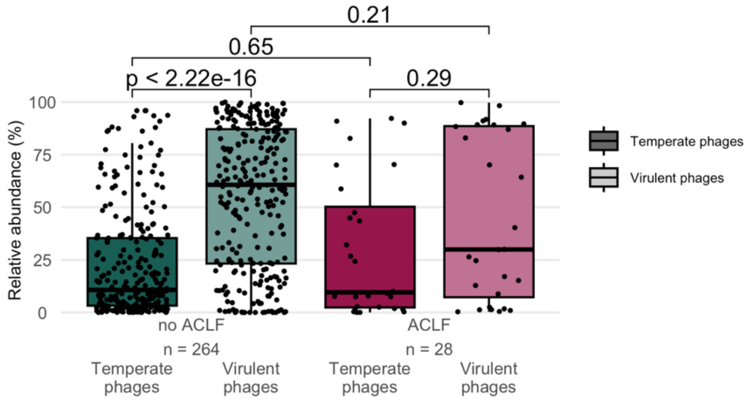

Fig. S6: Phageome composition in decompensated cirrhosis and ACLF. Phageome composition in terms of phage lifestyle in all samples with and without ACLF (Wilcoxon rank-sum test). ACLF = acute-on-chronic liver disease.

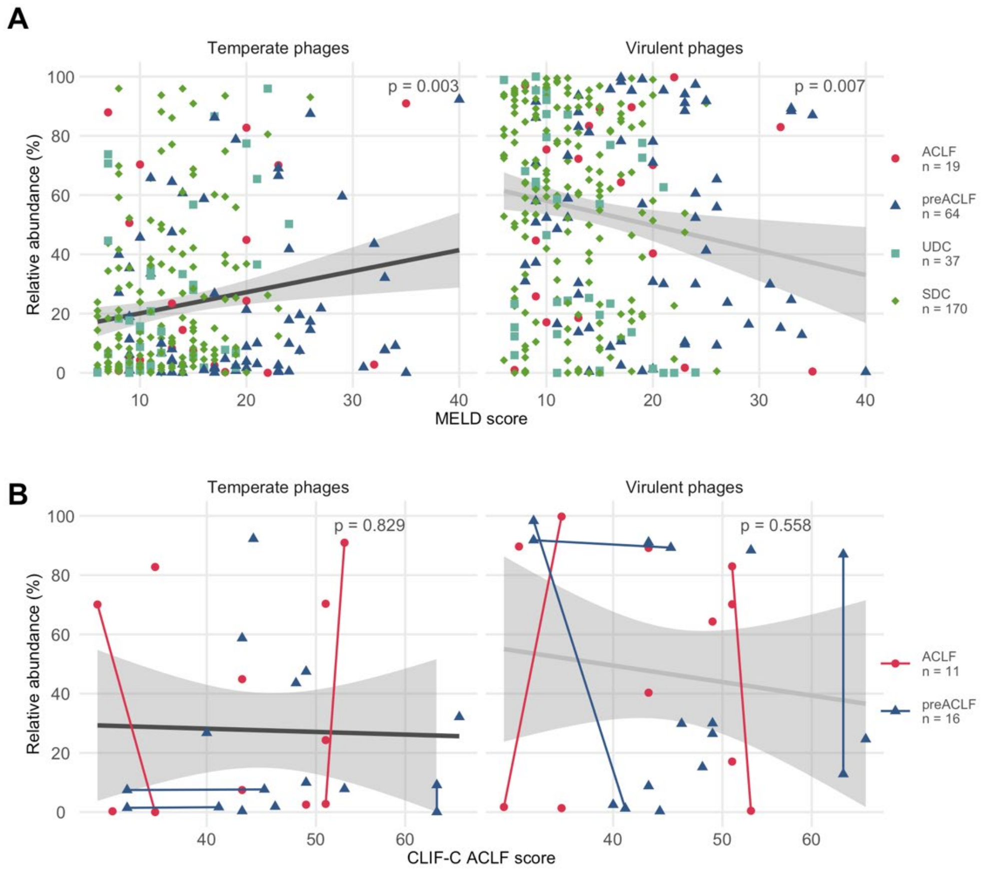

Fig. S7: Phage Simpson diversity associated with CLIF-C ACLF, but not MELD score

(A) Relationship between the relative abundance of temperate and virulent phages and MELD score across all visits of all patients (Pearson's correlation coefficient = 0.17 and -0.17). (B) Relationship between the relative abundance of temperate and virulent phages and CLIF-C ACLF score across all ACLF visits of all (pre)ACLF patients (Pearson's correlation coefficient = -0.04 (ns) and -0.12 (ns)). Samples from the same patient are connected.

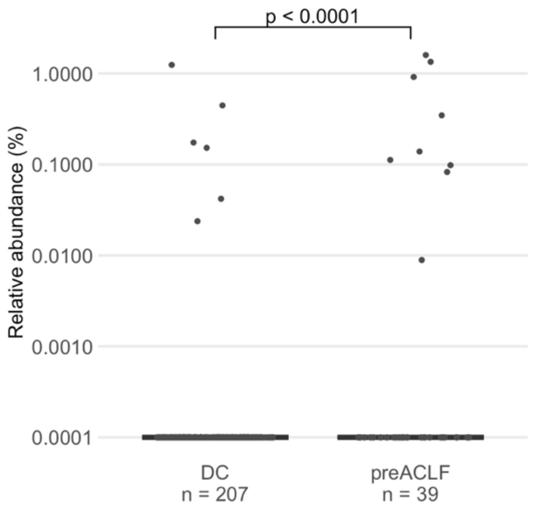

Fig. S8: *Lactococcus A* phages associated with pre-ACLF.

Relative abundance of *Lactococcus A* phages in the all samples of patients with decompensated cirrhosis vs. all samples of pre-ACLF patients before ACLF onset (Wilcoxon rank-sum test). DC = decompensated cirrhosis; ACLF = acute-on-chronic liver failure.

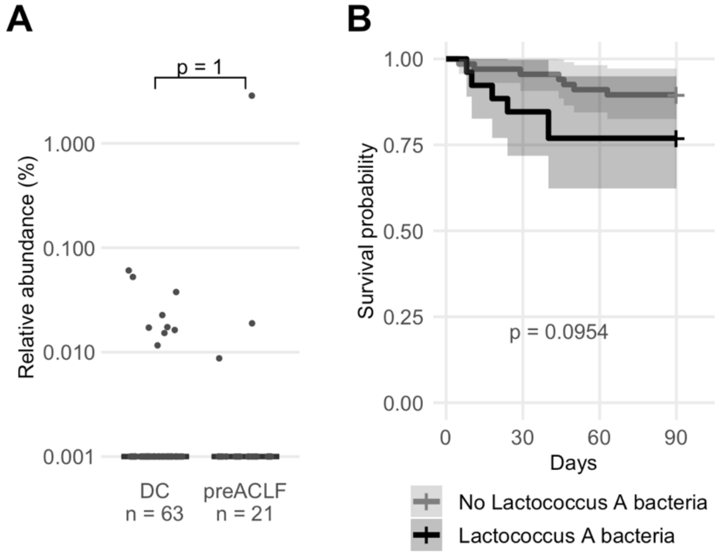

Fig. S9: *Lactococcus A* bacteria associated with higher short-term mortality, but not pre-ACLF.

(A) Relative abundance of *Lactococcus A* bacteria in the first visit of decompensated cirrhosis vs. pre-ACLF patients (Wilcoxon rank-sum test). (B) 90-day survival analysis of patients with and without *Lactococcus A* bacteria (first sample with these bacteria within 90 days from first sample; n = 26 vs.

first sample; n = 67) (Log-rank test). DC = decompensated cirrhosis; ACLF = acute-on-chronic liver failure.

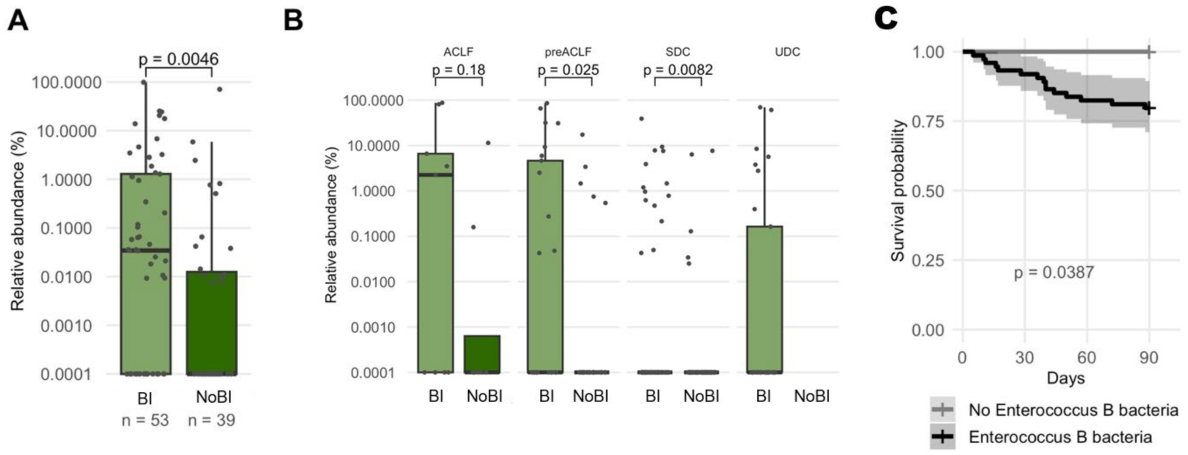

Fig. S10: *Enterococcus B* bacteria associated with bacterial infection and worse short-term survival.

(A) Relative abundance of *Enterococcus B* bacteria in patients with and without bacterial infection (Wilcoxon rank-sum test). (B) Relative abundance of *Enterococcus B* bacteria in patients with and without bacterial infection in patients with ACLF, preACLF, UDC and SDC (ACLF = acute-on-chronic liver failure; UDC = unstable decompensated cirrhosis; SDC = stable decompensated cirrhosis.) (C) 90-day survival analysis of patients with (n = 74) and without (n = 19) *Enterococcus B* bacteria (first sample with these bacteria within 90 days from first sample vs. first sample) (Log-rank test).

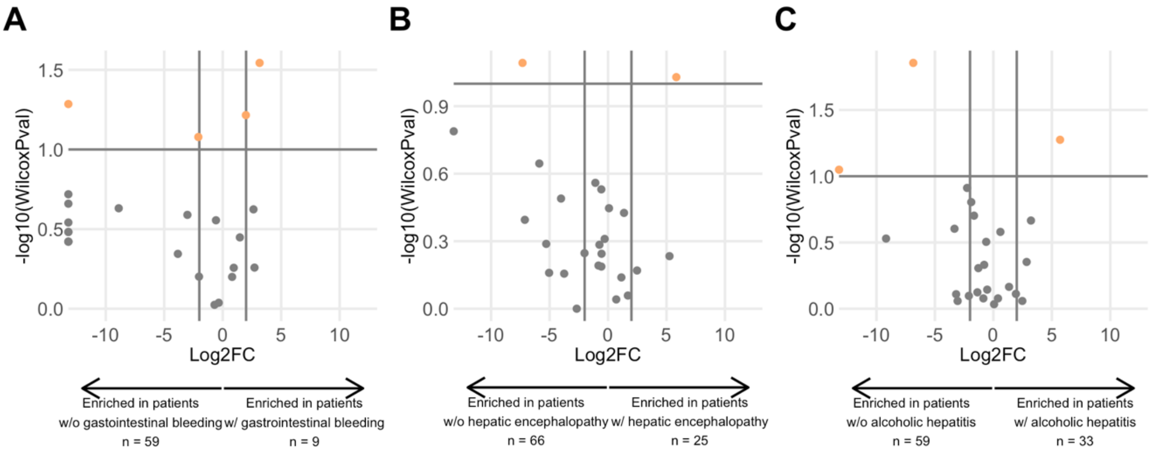

Fig. S11: Phage-host groups are not associated with any other precipitating events.

(A) Volcano plots to visualize changes in abundance of phage-host groups (n = 24) between patients with and without gastrointestinal bleeding present in at least 5/68 samples (first sample with gastrointestinal bleeding vs. first sample of patients without gastrointestinal bleeding). (B)

Volcano plots to visualize changes in abundance of phage-host groups (n = 25) between patients with and without hepatic encephalopathy present in at least 5/91 samples (first sample with hepatic encephalopathy vs. first sample of patients without hepatic encephalopathy). (C) Volcano plots to visualize changes in abundance of phage-host groups (n = 25) between patients with and without alcoholic hepatitis present in at least 5/92 samples (first sample with alcoholic hepatitis vs. first sample of patients without alcoholic hepatitis). (Wilcoxon rank-sum tests; orange dots = $p < 0.1$ and FDR < 0.1).

Supplementary Tables

Variable	Category	Univariate dbRDA					Multivariate dbRDA (n = 268)		
		n	R2	AdjR2	P	AdjP	AdjR2	AdjR2_0	P
ACLF	Disease	292	0.00379472	0.00035953	0.381	0.41189	NA	NA	NA
Active Alcoholism	Demographic	292	0.00332125	-0.0001156	0.64	0.64000	NA	NA	NA
Age	Demographic	292	0.00589354	0.00246558	0.008	**0.01778**	NA	NA	NA
Albumin - blood (sqrt)	Lab_Liver	285	0.00676459	0.00325492	0.001	**0.00400**	0.3244268	0.00411362	0.002
Albumin - medication	Medication	292	0.00545382	0.00202435	0.027	**0.04320**	NA	NA	NA
ALP (log)	Lab_Liver	284	0.00596202	0.00243706	0.019	**0.03455**	NA	NA	NA
ALT (log)	Lab_Liver	286	0.0055382	0.00203657	0.024	**0.04000**	NA	NA	NA
Antibiotics	Medication	292	0.00675498	0.00333	0.002	**0.00533**	NA	NA	NA
Ascites	Disease	292	0.00479708	0.00136535	0.061	**0.08414**	NA	NA	NA
Alcholic steatohepatitis	Disease	292	0.0041072	0.00067309	0.238	0.28000	NA	NA	NA
AST (log)	Lab_Liver	287	0.00630497	0.00281832	0.001	**0.00400**	NA	NA	NA
Bilirubin (log)	Lab_Liver	292	0.00704219	0.0036182	0.002	**0.00533**	NA	NA	NA
Bacterial infection	Disease	292	0.00726626	0.00384304	0.001	**0.00400**	NA	NA	NA
BMI	Demographic	292	0.00393887	0.00050417	0.309	0.35314	NA	NA	NA
Cardiologic failure	Disease	292	0.00382875	0.00039368	0.348	0.38667	NA	NA	NA
Cerebral failure	Disease	292	0.00347652	4.0236E-05	0.535	0.56316	NA	NA	NA
Coagulatory failure	Disease	292	0.00451563	0.00108293	0.126	0.15750	NA	NA	NA
Creatinine (log)	Lab_Liver	291	0.00476581	0.00132209	0.074	**0.09548**	NA	NA	NA
CRP (log)	Lab_Inflammation	292	0.00668853	0.00326332	0.004	**0.01000**	NA	NA	NA
Etiology	Disease	292	0.02543169	0.00839378	0.001	**0.00400**	NA	NA	NA
Gastrointestinal failure	Disease	292	0.00412347	0.00068941	0.23	0.27879	NA	NA	NA
GGT (log)	Lab_Liver	276	0.00541847	0.00178861	0.048	**0.07111**	NA	NA	NA
Group	Disease	292	0.01674557	0.00650334	0.001	**0.00400**	NA	NA	NA
Hepatic encephalopathy	Disease	292	0.00581041	0.00238217	0.011	**0.02095**	NA	NA	NA
PatientID	Demographic	292	0.52857428	0.31062872	0.001	**0.00400**	0.3203132	0.32031318	0.001
INR (inv)	Lab_Liver	290	0.00563943	0.00218679	0.024	**0.04000**	0.3275784	0.00315162	0.014
Liver dysfunction	Disease	292	0.00628629	0.00285969	0.007	**0.01647**	NA	NA	NA
Lymphocytes (log)	Lab_Inflammation	292	0.0048527	0.00142115	0.061	**0.08414**	NA	NA	NA
MELD score	Disease	290	0.00779135	0.00434618	0.002	**0.00533**	NA	NA	NA
MELD-Na score	Disease	290	0.00845563	0.00501277	0.001	**0.00400**	NA	NA	NA
Monocytes (log)	Lab_Inflammation	292	0.00612467	0.00269751	0.011	**0.02095**	NA	NA	NA
Sodium	Lab_Kidney	291	0.00952583	0.00609858	0.001	**0.00400**	NA	NA	NA
Neutrophils (log)	Lab_Inflammation	292	0.00738795	0.00396515	0.002	**0.00533**	NA	NA	NA
Protein-pump inhibitors	Medication	292	0.00661151	0.00318603	0.002	**0.00533**	NA	NA	NA
Renal dysfunction	Disease	291	0.00670037	0.00326334	0.001	**0.00400**	NA	NA	NA
Rifaximin	Medication	292	0.00506184	0.00163101	0.044	**0.06769**	NA	NA	NA
Sex	Demographic	292	0.00601041	0.00258286	0.009	**0.01895**	NA	NA	NA
Lactulose	Medication	292	0.00336686	-6.981E-05	0.613	0.62872	NA	NA	NA
Visit Day	Demographic	292	0.00485722	0.00142569	0.064	**0.08533**	0.3322015	0.00231562	0.049
WBC (log)	Lab_Inflammation	292	0.00768504	0.00426327	0.001	**0.00400**	0.3298859	0.0023075	0.02

Table S1: Individual effect size of each covariate for determing the high inter-sample diversity in phage community using univariate distance-based redundancy analysis (dbRDA).

Host_Genus	Prevalence				Fisher test for equal proportions			Wilcoxon rank-sum test		
	preACLF (n = 21)		SDC & UDC (n = 63)		OR	P	AdjP	Log2FC	P	AdjP
	n	%	n	%						
Alistipes	8	38.1%	22	34.9%	1.145	0.798	1.000	-1.097	0.759	0.976
Bacteroides	10	47.6%	34	54.0%	0.778	0.625	1.000	-2.488	0.388	0.976
Bifidobacterium	4	19.0%	6	9.5%	2.211	0.259	1.000	2.085	0.283	0.976
Blautia_A	0	0.0%	7	11.1%	0.000	0.184	1.000	-Inf	0.116	0.961
Chlamydia	4	19.0%	17	27.0%	0.640	0.570	1.000	-2.168	0.674	0.976
Dysosmobacter	2	9.5%	6	9.5%	1.000	1.000	1.000	-1.064	0.976	0.976
Enterocloster	2	9.5%	11	17.5%	0.501	0.502	1.000	0.593	0.403	0.976
Enterococcus_B	3	14.3%	7	11.1%	1.329	0.705	1.000	-4.746	0.854	0.976
Escherichia	4	19.0%	17	27.0%	0.640	0.570	1.000	1.175	0.625	0.976
Faecalibacterium	5	23.8%	15	23.8%	1.000	1.000	1.000	-2.849	0.945	0.976
Flavonifractor	3	14.3%	8	12.7%	1.144	1.000	1.000	-0.866	0.846	0.976
Fusicatenibacter	1	4.8%	4	6.3%	0.740	1.000	1.000	-6.301	0.762	0.976
Gemmiger	1	4.8%	5	7.9%	0.583	1.000	1.000	0.135	0.644	0.976
Klebsiella	2	9.5%	7	11.1%	0.844	1.000	1.000	2.061	0.908	0.976
Lactococcus	6	28.6%	20	31.7%	0.862	1.000	1.000	1.759	0.895	0.976
Lactococcus_A	4	19.0%	1	1.6%	14.001	0.013	0.322	7.090	0.003	**0.080**
Lawsonibacter	2	9.5%	3	4.8%	2.084	0.595	1.000	-2.733	0.450	0.976
Leuconostoc	5	23.8%	7	11.1%	2.469	0.164	1.000	0.784	0.164	0.961
Mediterraneibacter	0	0.0%	8	12.7%	0.000	0.192	1.000	-Inf	0.090	0.961
Odoribacter	3	14.3%	9	14.3%	1.000	1.000	1.000	-4.011	0.919	0.976
Parabacteroides	8	38.1%	20	31.7%	1.319	0.603	1.000	-0.131	0.763	0.976
Prevotella	2	9.5%	13	20.6%	0.409	0.336	1.000	0.730	0.330	0.976
Ruminococcus_B	1	4.8%	5	7.9%	0.583	1.000	1.000	-2.548	0.627	0.976
Streptococcus	4	19.0%	7	11.1%	1.867	0.455	1.000	-3.794	0.449	0.976
Veillonella	1	4.8%	10	15.9%	0.268	0.277	1.000	-3.696	0.192	0.961

Table S2: Phage-host groups in patients with preACLF compared to patients with SDC and UDC. ACLF = acute-on-chronic liver failure; UDC = unstable decompensated cirrhosis; SDC = stable decompensated cirrhosis.

Length	Estimated completeness	Genus	Family	Host prediction method (exc. Prophage)	Prophage species hits	BACPHLIP temperate	Lifestyle
19 kb	51%	Caudoviricetes	Family_2	PHIST + tRNA		Yes	Temperate
19 kb	56%	Caudoviricetes	Family_2	PHIST	L. laudensis (n = 1)	No	Temperate
* Species level host prediction only possible if prophage hit to a high-quality bacterial MAG.							

Table S3: Temperateness of *Lactococcus A* phages associated with future ACLF development

Host_Genus	Group sizes (n)		Observed events (n)		Expected events (n)		Cox regression model		
	Absent	Present	Absent	Present	Absent	Present	ChiSq	P	AdjP
Alistipes	54	39	7	6	7.454	5.546	0.065	0.799	0.940
Anaerobutyricum	88	5	13	0	12.252	0.748	0.795	0.373	0.638
Anaerostipes	88	5	13	0	12.252	0.748	0.795	0.373	0.638
Bacteroides	31	62	5	8	4.200	8.800	0.225	0.635	0.819
Barnesiella	88	5	12	1	12.347	0.653	0.194	0.659	0.824
Bifidobacterium	78	15	9	4	11.063	1.937	2.590	0.108	0.450
Blautia_A	79	14	12	1	10.906	2.094	0.683	0.409	0.654
Chlamydia	69	24	11	2	9.622	3.378	0.761	0.383	0.638
Clostridium_AQ	84	9	11	3	12.709	1.291	2.499	0.114	0.450
Dorea_A	84	9	11	4	13.659	1.341	5.823	0.016	0.209
Dysosmobacter	83	10	10	3	11.727	1.273	2.604	0.107	0.450
Enterobacter	86	7	13	0	11.953	1.047	1.141	0.285	0.616
Enterocloster	67	26	11	3	9.895	4.105	0.422	0.516	0.774
Enterococcus	88	5	11	2	12.349	0.651	2.951	0.086	0.450
Enterococcus_B	57	36	4	12	10.183	5.817	10.389	0.001	**0.025**
Erysipelatoclostridium	83	10	13	0	11.504	1.496	1.694	0.193	0.515
Escherichia	59	34	9	4	8.077	4.923	0.278	0.598	0.797
Faecalibacterium	64	29	9	4	8.806	4.194	0.013	0.908	0.981
Faecalimonas	85	8	13	0	11.804	1.196	1.321	0.250	0.589
Flavonifractor	71	22	12	1	9.710	3.290	2.140	0.144	0.450
Fusicatenibacter	88	5	12	1	12.252	0.748	0.090	0.764	0.926
Gemmiger	86	7	12	1	12.059	0.941	0.004	0.950	0.981
Hungatella	87	6	11	2	12.222	0.778	2.045	0.153	0.450
Klebsiella	73	20	9	5	11.231	2.769	2.241	0.134	0.450
Lactobacillus	85	8	13	0	11.804	1.196	1.321	0.250	0.589
Lactococcus	33	60	4	10	4.999	9.001	0.311	0.577	0.796
Lactococcus_A	80	13	6	7	11.564	1.436	24.393	0.000	**0.000**
Lawsonibacter	81	12	12	1	11.300	1.700	0.332	0.564	0.796
Leuconostoc	62	31	5	8	8.872	4.128	5.336	0.021	0.209
Massilioclostridium	88	5	11	2	12.453	0.547	4.039	0.044	0.356
Mediterraneibacter	81	12	12	1	11.205	1.795	0.409	0.522	0.774
Odoribacter	73	20	10	3	10.081	2.919	0.003	0.957	0.981
Parabacteroides	56	37	8	5	7.865	5.135	0.006	0.939	0.981
Prevotella	69	24	10	4	10.330	3.670	0.040	0.841	0.961
Roseburia	87	6	11	2	12.222	0.778	2.047	0.152	0.450
Rothia	88	5	13	0	12.252	0.748	0.795	0.373	0.638
Ruminococcus_B	86	7	12	2	13.009	0.991	1.107	0.293	0.616
Ruminococcus_E	87	6	13	0	12.103	0.897	0.966	0.326	0.638
Streptococcus	71	22	8	5	10.114	2.886	1.998	0.158	0.450
Veillonella	71	22	10	3	10.015	2.985	0.000	0.992	0.992

Table S4: Association between short-term (90-day) mortality and the presence of phage-host groups

Host_Genus	Prevalence				Fisher test for equal proportions			Wilcoxon rank-sum test		
	Bacterial infection (n = 53)		No bacterial infection (n = 39)		OR	P	AdjP	Log2FC	P	AdjP
	n	%	n	%						
Alistipes	22	41.5%	8	20.5%	2.72	0.043	0.543	1.741	0.051	0.641
Bacteroides	27	50.9%	22	56.4%	0.804	0.675	1.000	-0.520	0.381	0.975
Bifidobacterium	4	7.5%	6	15.4%	0.453	0.314	1.000	1.679	0.245	0.975
Blautia_A	5	9.4%	2	5.1%	1.914	0.695	1.000	3.400	0.419	0.975
Chlamydia	12	22.6%	6	15.4%	1.602	0.436	1.000	-1.675	0.462	0.975
Dysosmobacter	5	9.4%	3	7.7%	1.247	1.000	1.000	0.222	0.771	0.975
Enterocloster	10	18.9%	5	12.8%	1.574	0.571	1.000	0.433	0.404	0.975
Enterococcus_B	15	28.3%	1	2.6%	14.677	0.001	**0.037**	10.431	0.001	**0.030**
Escherichia	11	20.8%	9	23.1%	0.874	0.803	1.000	-3.859	0.554	0.975
Faecalibacterium	11	20.8%	9	23.1%	0.874	0.803	1.000	0.835	0.835	0.975
Flavonifractor	8	15.1%	4	10.3%	1.548	0.549	1.000	1.276	0.478	0.975
Fusicatenibacter	3	5.7%	2	5.1%	1.109	1.000	1.000	-0.648	0.936	0.975
Gemmiger	3	5.7%	3	7.7%	0.723	0.696	1.000	-3.579	0.658	0.975
Klebsiella	6	11.3%	2	5.1%	2.341	0.459	1.000	1.671	0.312	0.975
Lactococcus	20	37.7%	14	35.9%	1.081	1.000	1.000	-2.218	0.975	0.975
Lactococcus_A	4	7.5%	3	7.7%	0.98	1.000	1.000	-1.829	0.959	0.975
Lawsonibacter	3	5.7%	3	7.7%	0.723	0.696	1.000	3.484	0.685	0.975
Leuconostoc	8	15.1%	7	17.9%	0.815	0.779	1.000	0.462	0.778	0.975
Mediterraneibacter	5	9.4%	3	7.7%	1.247	1.000	1.000	-0.516	0.808	0.975
Odoribacter	8	15.1%	4	10.3%	1.548	0.549	1.000	1.566	0.548	0.975
Parabacteroides	16	30.2%	13	33.3%	0.866	0.822	1.000	0.947	0.931	0.975
Prevotella	6	11.3%	8	20.5%	0.499	0.253	1.000	-4.159	0.242	0.975
Rothia	4	7.5%	1	2.6%	3.069	0.391	1.000	7.815	0.286	0.975
Streptococcus	7	13.2%	5	12.8%	1.034	1.000	1.000	-1.304	0.957	0.975
Veillonella	6	11.3%	4	10.3%	1.116	1.000	1.000	4.423	0.776	0.975

Table S5: Phage-host groups in patients with a bacterial infection

Length	Estimated completeness	Genus	Family	Host prediction method (exc. Prophage)	Prophage species hits	BACPHLIP temperate	Lifestyle
25 kb	64%	Genus_65	Family_2	RaFAH + PHIST	E. faecium (n = 8) & E. hirae (n = 1)		Temperate
32 kb	82%	Genus_65	Family_2	RaFAH + PHIST	E. faecium (n = 6) & E. hirae (n = 1)		Temperate
33 kb	83%	Genus_65	Family_2	RaFAH + PHIST	E. faecium (n = 6) & E. hirae (n = 1)		Temperate
26 kb	66%	Genus_65	Family_2	RaFAH + PHIST	E. faecium (n = 6) & E. lactis (n = 1)		Temperate
26 kb	66%	Genus_65	Family_2	RaFAH + PHIST + tRNA	E. faecium (n = 8) & E. hirae (n = 1)	Yes	Temperate
34 kb	85%	Genus_65	Family_2	RaFAH + PHIST + tRNA	E. faecium (n = 7) & E. hirae (n = 1)	Yes	Temperate
27 kb	69%	Genus_65	Family_2	RaFAH + PHIST	E. faecium (n = 5) & E. lactis (n = 1)		Temperate
32 kb	83%	Genus_67	Family_2	RaFAH + PHIST	E. faecium (n = 16)		Temperate
72 kb	100%	Genus_67	Family_2	RaFAH + PHIST + tRNA	E. faecium (n = 13)	Yes	Temperate
19 kb	50%	Genus_67	Family_2	RaFAH + PHIST	E. faecium (n = 18)		Temperate
32 kb	83%	Genus_67	Family_2	RaFAH + PHIST	E. faecium (n = 17)	Yes	Temperate
42 kb	100%	Genus_67	Family_2	RaFAH + PHIST + tRNA	E. faecium (n = 14)	Yes	Temperate
38 kb	99%	Genus_67	Family_2	RaFAH + PHIST + tRNA	E. faecium (n = 14)	Yes	Temperate
19 kb	100%	Genus_35	Family_23	RaFAH			Virulent
22 kb	54%	Genus_120	Family_33	RaFAH + PHIST	E. durans (n = 1)	Yes	Temperate
26 kb	62%	Genus_27	Family_33	RaFAH + PHIST			Virulent
26 kb	60%	Genus_120	Family_42	RaFAH + PHIST	E. faecium (n = 2) & E. hirae (n = 1)		Temperate
21 kb	50%	Genus_273	Family_61	RaFAH + PHIST			Virulent
* Species level host prediction only possible if prophage hit to a high-quality bacterial MAG.							

Table S6: Lifestyle prediction of *Enterococcus B* phages

Host_Genus	Prevalence				Fisher test for equal proportions			Wilcoxon rank-sum test		
	Gastrointestinal bleeding (n = 9)		No gastrointestinal bleeding (n = 59)		OR	P	AdjP	Log2FC	P	AdjP
	n	%	n	%						
Alistipes	2	22.2%	20	33.9%	0.562	0.707	1.000	-3.834	0.452	0.603
Bacteroides	2	22.2%	33	55.9%	0.230	0.079	0.545	-2.081	0.084	0.501
Bifidobacterium	0	0.0%	6	10.2%	0.000	1.000	1.000	-Inf	0.330	0.535
Blautia_A	2	22.2%	5	8.5%	3.017	0.230	0.907	-3.019	0.257	0.535
Chlamydia	3	33.3%	14	23.7%	1.595	0.680	1.000	2.719	0.552	0.664
Dysosmobacter	0	0.0%	7	11.9%	0.000	0.582	1.000	-Inf	0.287	0.535
Enterocloster	2	22.2%	7	11.9%	2.094	0.340	0.907	1.470	0.357	0.535
Enterococcus_B	4	44.4%	9	15.3%	4.315	0.060	0.545	3.156	0.029	0.487
Escherichia	4	44.4%	14	23.7%	2.530	0.231	0.907	-0.575	0.278	0.535
Faecalibacterium	1	11.1%	16	27.1%	0.340	0.432	1.000	-8.892	0.234	0.535
Flavonifractor	1	11.1%	6	10.2%	1.103	1.000	1.000	-0.339	0.918	0.945
Fusicatenibacter	0	0.0%	5	8.5%	0.000	1.000	1.000	-Inf	0.379	0.535
Gemmiger	0	0.0%	5	8.5%	0.000	1.000	1.000	-Inf	0.379	0.535
Klebsiella	3	33.3%	6	10.2%	4.280	0.091	0.545	1.982	0.061	0.487
Lactococcus	2	22.2%	18	30.5%	0.655	1.000	1.000	-2.024	0.629	0.689
Lawsonibacter	0	0.0%	5	8.5%	0.000	1.000	1.000	-Inf	0.379	0.535
Leuconostoc	1	11.1%	6	10.2%	1.103	1.000	1.000	-0.699	0.945	0.945
Mediterraneibacter	0	0.0%	6	10.2%	0.000	1.000	1.000	-Inf	0.330	0.535
Odoribacter	0	0.0%	9	15.3%	0.000	0.595	1.000	-Inf	0.219	0.535
Parabacteroides	0	0.0%	19	32.2%	0.000	0.053	0.545	-Inf	0.052	0.487
Prevotella	0	0.0%	10	16.9%	0.000	0.336	0.907	-Inf	0.191	0.535
Ruminococcus_B	1	11.1%	4	6.8%	1.703	0.520	1.000	0.806	0.631	0.689
Streptococcus	2	22.2%	9	15.3%	1.575	0.631	1.000	0.945	0.553	0.664
Veillonella	2	22.2%	6	10.2%	2.480	0.285	0.907	2.633	0.238	0.535

Table S7: Association of phage-host groups with gastrointestinal bleeding

Host_Genus	Prevalence				Fisher test for equal proportions			Wilcoxon rank-sum test		
	Hepatic encephalopathy (n = 25)		No hepatic encephalopathy (n = 66)		OR	P	AdjP	Log2FC	P	AdjP
	n	%	n	%						
Alistipes	10	40.0%	20	30.3%	1.526	0.456	1.000	0.082	0.358	0.825
Bacteroides	15	60.0%	34	51.5%	1.406	0.491	1.000	-0.573	0.650	0.825
Bifidobacterium	2	8.0%	8	12.1%	0.633	0.721	1.000	2.480	0.676	0.825
Blautia_A	1	4.0%	6	9.1%	0.420	0.669	1.000	-7.116	0.403	0.825
Chlamydia	6	24.0%	15	22.7%	1.073	1.000	1.000	-2.678	1.000	1.000
Dysosmobacter	1	4.0%	8	12.1%	0.305	0.435	1.000	-5.880	0.226	0.825
Enterocloster	4	16.0%	9	13.6%	1.204	0.747	1.000	1.140	0.726	0.825
Enterococcus_B	1	4.0%	12	18.2%	0.190	0.104	1.000	-7.316	0.081	0.825
Escherichia	7	28.0%	14	21.2%	1.438	0.579	1.000	-0.817	0.643	0.825
Faecalibacterium	4	16.0%	18	27.3%	0.511	0.411	1.000	-0.575	0.295	0.825
Flavonifractor	4	16.0%	7	10.6%	1.596	0.486	1.000	-0.290	0.490	0.825
Fusicatenibacter	0	0.0%	5	7.6%	0.000	0.317	1.000	-Inf	0.163	0.825
Gemmiger	1	4.0%	5	7.6%	0.512	1.000	1.000	-5.288	0.515	0.825
Klebsiella	1	4.0%	8	12.1%	0.305	0.435	1.000	-1.087	0.276	0.825
Lactococcus	9	36.0%	20	30.3%	1.290	0.622	1.000	1.366	0.375	0.825
Lawsonibacter	1	4.0%	5	7.6%	0.512	1.000	1.000	5.255	0.584	0.825
Leuconostoc	3	12.0%	9	13.6%	0.865	1.000	1.000	1.698	0.874	0.947
Mediterraneibacter	1	4.0%	7	10.6%	0.354	0.437	1.000	-4.020	0.324	0.825
Odoribacter	3	12.0%	10	15.2%	0.766	1.000	1.000	-3.754	0.699	0.825
Parabacteroides	7	28.0%	21	31.8%	0.835	0.803	1.000	0.701	0.909	0.947
Prevotella	4	16.0%	12	18.2%	0.859	1.000	1.000	-5.041	0.693	0.825
Rothia	3	12.0%	2	3.0%	4.279	0.125	1.000	5.837	0.093	0.825
Ruminococcus_B	1	4.0%	5	7.6%	0.512	1.000	1.000	-0.555	0.570	0.825
Streptococcus	3	12.0%	11	16.7%	0.685	0.750	1.000	-2.013	0.566	0.825
Veillonella	2	8.0%	9	13.6%	0.554	0.721	1.000	-0.735	0.520	0.825

Table S8: Association of phage-host groups with hepatic encephalopathy

Host_Genus	Prevalence				Fisher test for equal proportions			Wilcoxon rank-sum test		
	Alcoholic hepatitis (n = 33)		No alcoholic hepatitis (n = 59)		OR	P	AdjP	Log2FC	P	AdjP
	n	%	n	%						
Alistipes	9	27.3%	21	35.6%	0.681	0.491	0.876	-1.287	0.494	0.882
Bacteroides	20	60.6%	29	49.2%	1.583	0.384	0.863	-0.617	0.313	0.711
Bifidobacterium	6	18.2%	5	8.5%	2.376	0.193	0.863	-1.657	0.199	0.711
Blautia_A	3	9.1%	4	6.8%	1.370	0.698	1.000	1.338	0.684	0.911
Chlamydia	3	9.1%	18	30.5%	0.231	0.021	0.522	-6.854	0.014	0.349
Dysosmobacter	3	9.1%	4	6.8%	1.370	0.698	1.000	-3.186	0.777	0.911
Enterocloster	5	15.2%	8	13.6%	1.137	1.000	1.000	0.058	0.925	0.925
Enterococcus_B	8	24.2%	6	10.2%	2.792	0.127	0.863	5.702	0.053	0.663
Escherichia	8	24.2%	14	23.7%	1.028	1.000	1.000	1.925	0.773	0.911
Faecalibacterium	7	21.2%	13	22.0%	0.953	1.000	1.000	-1.368	0.752	0.911
Flavonifractor	4	12.1%	7	11.9%	1.024	1.000	1.000	2.472	0.874	0.911
Fusicatenibacter	2	6.1%	3	5.1%	1.202	1.000	1.000	0.394	0.836	0.911
Gemmiger	3	9.1%	2	3.4%	2.815	0.346	0.863	0.598	0.263	0.711
Klebsiella	3	9.1%	7	11.9%	0.745	1.000	1.000	-0.528	0.718	0.911
Lactococcus	12	36.4%	15	25.4%	1.667	0.341	0.863	-0.789	0.466	0.882
Lactococcus_A	2	6.1%	3	5.1%	1.202	1.000	1.000	-0.857	0.836	0.911
Lawsonibacter	0	0.0%	5	8.5%	0.000	0.156	0.863	-Inf	0.089	0.711
Leuconostoc	7	21.2%	5	8.5%	2.871	0.109	0.863	-2.248	0.123	0.711
Mediterraneibacter	1	3.0%	7	11.9%	0.235	0.251	0.863	-1.894	0.157	0.711
Odoribacter	7	21.2%	7	11.9%	1.984	0.243	0.863	3.213	0.216	0.711
Parabacteroides	12	36.4%	17	28.8%	1.406	0.489	0.876	-3.060	0.874	0.911
Prevotella	6	18.2%	9	15.3%	1.232	0.772	1.000	-2.100	0.800	0.911
Ruminococcus_B	1	3.0%	5	8.5%	0.341	0.414	0.863	-9.180	0.296	0.711
Streptococcus	6	18.2%	7	11.9%	1.641	0.534	0.890	2.844	0.444	0.882
Veillonella	2	6.1%	8	13.6%	0.415	0.321	0.863	-3.328	0.249	0.711

Table S9: Association of phage-host groups with alcoholic hepatitis

**Multivariable logistic regression - Bacterial infection in TIPS cohort**					
Coefficients:					
	Estimate	Std. Error	z value	Pr(>|z|)	
(Intercept)	1.737737	1.915298	0.907	0.364	
EntB_TIPS$Abundance	-15.94348	7.801731	-2.044	0.041	*
EntB_TIPS$CLIFCAD	0.006111	0.040486	0.151	0.88	
EntB_TIPS$AB2	-0.592219	0.676295	-0.876	0.381	
EntB_TIPS$Indication1	1.510591	1.114812	1.355	0.175	
Significance code: < 0.05 '*'					
(Dispersion parameter for binomial family taken to be 1)					
Null deviance: 75.694 on 83 degrees of freedom					
Residual deviance: 59.010 on 79 degrees of freedom					
(10 observations deleted due to missingness)					
AIC: 69.01					
Number of Fisher Scoring iterations: 6					

Table S10: Multivariable logistic regression for presence of bacterial infections in the validation cohort.

Length	Estimated completeness	Genus	Family	Host prediction method (exc. Prophage)	Prophage species hits	BACPHLIP temperate	Lifestyle
41 kb	99%	Genus_1082	Family_11	RaFAH + PHIST		Yes	Temperate
29 kb	76%	Genus_1213	Family_11	RaFAH + PHIST + tRNA	E. faecium (n = 2)	Yes	Temperate
36 kb	95%	Genus_1964	Family_11	RaFAH + PHIST		Yes	Temperate
43 kb	100%	Genus_317	Family_11	RaFAH + PHIST + tRNA	E. faecium (n = 1) & E. lactis (n = 1)	Yes	Temperate
48 kb	100%	Genus_317	Family_11	RaFAH + PHIST + tRNA	E. faecium (n = 1) & E. lactis (n = 1)	Yes	Temperate
23 kb	62%	Genus_317	Family_11	RaFAH + PHIST	E. faecium (n = 1)	Yes	Temperate
39 kb	100%	Genus_317	Family_11	RaFAH + PHIST + tRNA		Yes	Temperate
37 kb	100%	Genus_317	Family_11	RaFAH + PHIST + tRNA	E. faecium (n = 1) & E. hirae (n = 1)	Yes	Temperate
28 kb	76%	Genus_317	Family_11	RaFAH + PHIST + tRNA		Yes	Temperate
40 kb	100%	Genus_317	Family_11	RaFAH + PHIST	E. faecium (n = 1) & E. lactis (n = 1)	Yes	Temperate
33 kb	83%	Genus_317	Family_11	RaFAH + PHIST	E. faecium (n = 1) & E. lactis (n = 1)		Temperate
33 kb	84%	Genus_317	Family_11	RaFAH + PHIST + tRNA	E. faecium (n = 2) & E. hirae (n = 1)	Yes	Temperate
28 kb	70%	Genus_317	Family_11	RaFAH + PHIST	E. faecium (n = 1) & E. lactis (n = 2)		Temperate
45 kb	100%	Genus_317	Family_11	RaFAH + PHIST + tRNA	E. hirae (n = 1)	Yes	Temperate
26 kb	60%	Genus_1979	Family_11	RaFAH + PHIST			Virulent
22 kb	54%	Efquatrovirus	Family_127	RaFAH + PHIST			Virulent
22 kb	52%	Genus_899	Family_201	RaFAH + PHIST + tRNA	E. faecium (n = 3) & E. lactis (n = 1)		Temperate
* Species level host prediction only possible if prophage hit to a high-quality bacterial MAG.							

Table S11: Temperateness of *Enterococcus B* phages in the validation cohort

BioProject accession	BioSample accession	Run accession
ERP172927	ERS25304041	ERR15372593
ERP172927	ERS25304042	ERR15372805
ERP172927	ERS25304043	ERR15372796
ERP172927	ERS25304044	ERR15372718
ERP172927	ERS25304045	ERR15372822
ERP172927	ERS25304046	ERR15372740
ERP172927	ERS25304047	ERR15372727
ERP172927	ERS25304048	ERR15372838
ERP172927	ERS25304049	ERR15372751
ERP172927	ERS25304050	ERR15372630
ERP172927	ERS25304051	ERR15372588
ERP172927	ERS25304052	ERR15372799
ERP172927	ERS25304053	ERR15372716
ERP172927	ERS25304054	ERR15372812
ERP172927	ERS25304055	ERR15372738
ERP172927	ERS25304056	ERR15372754
ERP172927	ERS25304057	ERR15372674
ERP172927	ERS25304058	ERR15372652
ERP172927	ERS25304059	ERR15372861
ERP172927	ERS25304060	ERR15372743
ERP172927	ERS25304061	ERR15372801
ERP172927	ERS25304062	ERR15372569
ERP172927	ERS25304063	ERR15372678
ERP172927	ERS25304064	ERR15372810
ERP172927	ERS25304065	ERR15372641
ERP172927	ERS25304066	ERR15372722
ERP172927	ERS25304067	ERR15372821
ERP172927	ERS25304068	ERR15372715
ERP172927	ERS25304069	ERR15372850

ERP172927	ERS25304070	ERR15372612
ERP172927	ERS25304071	ERR15372840
ERP172927	ERS25304072	ERR15372755
ERP172927	ERS25304073	ERR15372582
ERP172927	ERS25304074	ERR15372638
ERP172927	ERS25304075	ERR15372785
ERP172927	ERS25304076	ERR15372592
ERP172927	ERS25304077	ERR15372845
ERP172927	ERS25304078	ERR15372833
ERP172927	ERS25304079	ERR15372573
ERP172927	ERS25304080	ERR15372865
ERP172927	ERS25304081	ERR15372711
ERP172927	ERS25304082	ERR15372784
ERP172927	ERS25304083	ERR15372645
ERP172927	ERS25304084	ERR15372752
ERP172927	ERS25304085	ERR15372766
ERP172927	ERS25304086	ERR15372618
ERP172927	ERS25304087	ERR15372848
ERP172927	ERS25304088	ERR15372611
ERP172927	ERS25304089	ERR15372579
ERP172927	ERS25304090	ERR15372675
ERP172927	ERS25304092	ERR15372683
ERP172927	ERS25304093	ERR15372607
ERP172927	ERS25304094	ERR15372844
ERP172927	ERS25304095	ERR15372590
ERP172927	ERS25304096	ERR15372596
ERP172927	ERS25304097	ERR15372729
ERP172927	ERS25304098	ERR15372717
ERP172927	ERS25304099	ERR15372773
ERP172927	ERS25304100	ERR15372719
ERP172927	ERS25304101	ERR15372581
ERP172927	ERS25304102	ERR15372837
ERP172927	ERS25304103	ERR15372636
ERP172927	ERS25304104	ERR15372650
ERP172927	ERS25304105	ERR15372673
ERP172927	ERS25304106	ERR15372774
ERP172927	ERS25304107	ERR15372798
ERP172927	ERS25304108	ERR15372586
ERP172927	ERS25304109	ERR15372699
ERP172927	ERS25304110	ERR15372760
ERP172927	ERS25304111	ERR15372742
ERP172927	ERS25304112	ERR15372648
ERP172927	ERS25304113	ERR15372666
ERP172927	ERS25304114	ERR15372866

ERP172927	ERS25304115	ERR15372658
ERP172927	ERS25304116	ERR15372827
ERP172927	ERS25304117	ERR15372599
ERP172927	ERS25304118	ERR15372747
ERP172927	ERS25304119	ERR15372728
ERP172927	ERS25304120	ERR15372712
ERP172927	ERS25304121	ERR15372655
ERP172927	ERS25304122	ERR15372775
ERP172927	ERS25304123	ERR15372709
ERP172927	ERS25304124	ERR15372707
ERP172927	ERS25304125	ERR15372772
ERP172927	ERS25304126	ERR15372576
ERP172927	ERS25304127	ERR15372736
ERP172927	ERS25304128	ERR15372620
ERP172927	ERS25304129	ERR15372705
ERP172927	ERS25304130	ERR15372831
ERP172927	ERS25304131	ERR15372830
ERP172927	ERS25304132	ERR15372813
ERP172927	ERS25304133	ERR15372696
ERP172927	ERS25304134	ERR15372841
ERP172927	ERS25304135	ERR15372878
ERP172927	ERS25304136	ERR15372687
ERP172927	ERS25304137	ERR15372698
ERP172927	ERS25304138	ERR15372642
ERP172927	ERS25304139	ERR15372803
ERP172927	ERS25304140	ERR15372748
ERP172927	ERS25304141	ERR15372744
ERP172927	ERS25304142	ERR15372859
ERP172927	ERS25304143	ERR15372594
ERP172927	ERS25304144	ERR15372568
ERP172927	ERS25304145	ERR15372703
ERP172927	ERS25304146	ERR15372591
ERP172927	ERS25304147	ERR15372860
ERP172927	ERS25304149	ERR15372834
ERP172927	ERS25304150	ERR15372587
ERP172927	ERS25304151	ERR15372794
ERP172927	ERS25304152	ERR15372706
ERP172927	ERS25304153	ERR15372677
ERP172927	ERS25304154	ERR15372807
ERP172927	ERS25304155	ERR15372790
ERP172927	ERS25304156	ERR15372828
ERP172927	ERS25304157	ERR15372753
ERP172927	ERS25304158	ERR15372676
ERP172927	ERS25304159	ERR15372634

ERP172927	ERS25304160	ERR15372873
ERP172927	ERS25304161	ERR15372768
ERP172927	ERS25304162	ERR15372809
ERP172927	ERS25304163	ERR15372704
ERP172927	ERS25304164	ERR15372782
ERP172927	ERS25304165	ERR15372572
ERP172927	ERS25304166	ERR15372691
ERP172927	ERS25304167	ERR15372720
ERP172927	ERS25304168	ERR15372580
ERP172927	ERS25304169	ERR15372605
ERP172927	ERS25304170	ERR15372758
ERP172927	ERS25304171	ERR15372684
ERP172927	ERS25304172	ERR15372695
ERP172927	ERS25304173	ERR15372786
ERP172927	ERS25304174	ERR15372872
ERP172927	ERS25304175	ERR15372874
ERP172927	ERS25304176	ERR15372765
ERP172927	ERS25304177	ERR15372868
ERP172927	ERS25304178	ERR15372797
ERP172927	ERS25304179	ERR15372616
ERP172927	ERS25304180	ERR15372791
ERP172927	ERS25304181	ERR15372778
ERP172927	ERS25304182	ERR15372700
ERP172927	ERS25304183	ERR15372864
ERP172927	ERS25304184	ERR15372849
ERP172927	ERS25304185	ERR15372629
ERP172927	ERS25304186	ERR15372854
ERP172927	ERS25304187	ERR15372858
ERP172927	ERS25304189	ERR15372603
ERP172927	ERS25304190	ERR15372852
ERP172927	ERS25304191	ERR15372816
ERP172927	ERS25304192	ERR15372686
ERP172927	ERS25304193	ERR15372633
ERP172927	ERS25304194	ERR15372584
ERP172927	ERS25304195	ERR15372857
ERP172927	ERS25304196	ERR15372731
ERP172927	ERS25304197	ERR15372746
ERP172927	ERS25304198	ERR15372876
ERP172927	ERS25304199	ERR15372606
ERP172927	ERS25304200	ERR15372661
ERP172927	ERS25304201	ERR15372811
ERP172927	ERS25304202	ERR15372776
ERP172927	ERS25304203	ERR15372820
ERP172927	ERS25304204	ERR15372628

ERP172927	ERS25304205	ERR15372670
ERP172927	ERS25304206	ERR15372614
ERP172927	ERS25304207	ERR15372631
ERP172927	ERS25304208	ERR15372624
ERP172927	ERS25304209	ERR15372862
ERP172927	ERS25304210	ERR15372763
ERP172927	ERS25304211	ERR15372867
ERP172927	ERS25304212	ERR15372585
ERP172927	ERS25304213	ERR15372804
ERP172927	ERS25304214	ERR15372646
ERP172927	ERS25304215	ERR15372602
ERP172927	ERS25304216	ERR15372832
ERP172927	ERS25304217	ERR15372597
ERP172927	ERS25304218	ERR15372571
ERP172927	ERS25304219	ERR15372623
ERP172927	ERS25304220	ERR15372737
ERP172927	ERS25304221	ERR15372713
ERP172927	ERS25304222	ERR15372745
ERP172927	ERS25304223	ERR15372800
ERP172927	ERS25304225	ERR15372808
ERP172927	ERS25304226	ERR15372575
ERP172927	ERS25304227	ERR15372651
ERP172927	ERS25304228	ERR15372749
ERP172927	ERS25304229	ERR15372643
ERP172927	ERS25304230	ERR15372759
ERP172927	ERS25304231	ERR15372610
ERP172927	ERS25304232	ERR15372788
ERP172927	ERS25304233	ERR15372644
ERP172927	ERS25304234	ERR15372793
ERP172927	ERS25304235	ERR15372725
ERP172927	ERS25304236	ERR15372853
ERP172927	ERS25304237	ERR15372781
ERP172927	ERS25304238	ERR15372870
ERP172927	ERS25304239	ERR15372787
ERP172927	ERS25304240	ERR15372679
ERP172927	ERS25304242	ERR15372780
ERP172927	ERS25304243	ERR15372627
ERP172927	ERS25304244	ERR15372653
ERP172927	ERS25304245	ERR15372697
ERP172927	ERS25304246	ERR15372665
ERP172927	ERS25304247	ERR15372847
ERP172927	ERS25304248	ERR15372789
ERP172927	ERS25304249	ERR15372734
ERP172927	ERS25304250	ERR15372672

ERP172927	ERS25304251	ERR15372732
ERP172927	ERS25304252	ERR15372735
ERP172927	ERS25304253	ERR15372649
ERP172927	ERS25304254	ERR15372682
ERP172927	ERS25304255	ERR15372818
ERP172927	ERS25304256	ERR15372771
ERP172927	ERS25304257	ERR15372777
ERP172927	ERS25304258	ERR15372730
ERP172927	ERS25304259	ERR15372680
ERP172927	ERS25304260	ERR15372656
ERP172927	ERS25304261	ERR15372842
ERP172927	ERS25304262	ERR15372823
ERP172927	ERS25304263	ERR15372640
ERP172927	ERS25304264	ERR15372815
ERP172927	ERS25304265	ERR15372615
ERP172927	ERS25304266	ERR15372669
ERP172927	ERS25304267	ERR15372681
ERP172927	ERS25304268	ERR15372843
ERP172927	ERS25304269	ERR15372604
ERP172927	ERS25304270	ERR15372617
ERP172927	ERS25304271	ERR15372608
ERP172927	ERS25304272	ERR15372625
ERP172927	ERS25304273	ERR15372689
ERP172927	ERS25304274	ERR15372702
ERP172927	ERS25304275	ERR15372622
ERP172927	ERS25304276	ERR15372814
ERP172927	ERS25304277	ERR15372741
ERP172927	ERS25304278	ERR15372802
ERP172927	ERS25304279	ERR15372871
ERP172927	ERS25304280	ERR15372708
ERP172927	ERS25304281	ERR15372574
ERP172927	ERS25304282	ERR15372756
ERP172927	ERS25304283	ERR15372632
ERP172927	ERS25304284	ERR15372877
ERP172927	ERS25304285	ERR15372726
ERP172927	ERS25304286	ERR15372839
ERP172927	ERS25304287	ERR15372764
ERP172927	ERS25304288	ERR15372767
ERP172927	ERS25304289	ERR15372733
ERP172927	ERS25304290	ERR15372663
ERP172927	ERS25304291	ERR15372826
ERP172927	ERS25304292	ERR15372693
ERP172927	ERS25304293	ERR15372829
ERP172927	ERS25304294	ERR15372577

ERP172927	ERS25304295	ERR15372701
ERP172927	ERS25304296	ERR15372750
ERP172927	ERS25304298	ERR15372770
ERP172927	ERS25304299	ERR15372783
ERP172927	ERS25304300	ERR15372667
ERP172927	ERS25304301	ERR15372819
ERP172927	ERS25304302	ERR15372757
ERP172927	ERS25304303	ERR15372710
ERP172927	ERS25304304	ERR15372637
ERP172927	ERS25304305	ERR15372792
ERP172927	ERS25304306	ERR15372635
ERP172927	ERS25304307	ERR15372578
ERP172927	ERS25304308	ERR15372664
ERP172927	ERS25304310	ERR15372654
ERP172927	ERS25304311	ERR15372668
ERP172927	ERS25304312	ERR15372671
ERP172927	ERS25304313	ERR15372856
ERP172927	ERS25304314	ERR15372639
ERP172927	ERS25304315	ERR15372685
ERP172927	ERS25304316	ERR15372863
ERP172927	ERS25304317	ERR15372595
ERP172927	ERS25304318	ERR15372613
ERP172927	ERS25304320	ERR15372721
ERP172927	ERS25304321	ERR15372657
ERP172927	ERS25304322	ERR15372659
ERP172927	ERS25304323	ERR15372851
ERP172927	ERS25304324	ERR15372855
ERP172927	ERS25304325	ERR15372714
ERP172927	ERS25304326	ERR15372609
ERP172927	ERS25304327	ERR15372589
ERP172927	ERS25304328	ERR15372601
ERP172927	ERS25304329	ERR15372660
ERP172927	ERS25304330	ERR15372836
ERP172927	ERS25304331	ERR15372662
ERP172927	ERS25304332	ERR15372806
ERP172927	ERS25304333	ERR15372583
ERP172927	ERS25304334	ERR15372692
ERP172927	ERS25304335	ERR15372739
ERP172927	ERS25304336	ERR15372835
ERP172927	ERS25304337	ERR15372621
ERP172927	ERS25304338	ERR15372817
ERP172927	ERS25304339	ERR15372869
ERP172927	ERS25304340	ERR15372875
ERP172927	ERS25304341	ERR15372769

ERP172927	ERS25304342	ERR15372761
ERP172927	ERS25304343	ERR15372688
ERP172927	ERS25304344	ERR15372694
ERP172927	ERS25304345	ERR15372762
ERP172927	ERS25304346	ERR15372570
ERP172927	ERS25304347	ERR15372647
ERP172927	ERS25304348	ERR15372619
ERP172927	ERS25304349	ERR15372795

Table S12: Sequencing data are publicly available at: https://www.ebi.ac.uk/ena . The ENA accession numbers are provided in this table. ENA – European Nucleotide Archive.

Supplementary References

[1] **Conceição-Neto, N, Zeller, M**, Lefrère, H, et al. Modular approach to customise sample preparation procedures for viral metagenomics: a reproducible protocol for virome analysis. *Nat. Publ. Group*. 2015

[2] Bolger, AM, Lohse, M, and Usadel, B. Trimmomatic: A flexible trimmer for Illumina sequence data. *Bioinformatics*. 2014;30. 2114–2120

[3] Bankevich, A, Nurk, S, Antipov, D, et al. SPAdes: A New Genome Assembly Algorithm and Its Applications to Single-Cell Sequencing. *J. Comput. Biol.* 2012;19. 455–477

[4] Shen, W, Le, S, Li, Y, et al. SeqKit: A cross-platform and ultrafast toolkit for FASTA/Q file manipulation. *PLoS ONE*. 2016;11. 1–10

[5] Camacho, C, Coulouris, G, Avagyan, V, et al. BLAST+: architecture and applications. *BMC Bioinformatics*. 2009;10. 421

[6] **Nayfach, S, Camargo, AP**, Schulz, F, et al. CheckV assesses the quality and completeness of metagenome-assembled viral genomes. *Nat. Biotechnol.* 2020. 1–8

[7] Wood, DE, Lu, J, and Langmead, B. Improved metagenomic analysis with Kraken 2. *Genome Biol.* 2019;20. 1–13

[8] Buchfink, B, Xie, C, and Huson, DH. Fast and sensitive protein alignment using DIAMOND. *Nat. Methods*. 2014;12. 59–60

[9] Ondov, BD, Bergman, NH, and Phillippy, AM. Interactive metagenomic visualization in a Web browser. *BMC Bioinformatics*. 2011;12. 385

[10] **Guo, J, Bolduc, B**, Zayed, AA, et al. VirSorter2: a multi-classifier, expert-guided approach to detect diverse DNA and RNA viruses. *Microbiome*. 2021. 1–13

[11] Li, H, and Durbin, R. Fast and accurate short read alignment with Burrows-Wheeler transform. *Bioinformatics*. 2009;25. 1754–1760

[12] Li, H, Handsaker, B, Wysoker, A, et al. The Sequence Alignment/Map format and SAMtools. *Bioinformatics*. 2009;25. 2078–2079

[13] Davis, NM, Proctor, DM, Holmes, SP, et al. Simple statistical identification and removal of contaminant sequences in marker-gene and metagenomics data. *Microbiome*. 2018;6. 226

[14] McMurdie, PJ, and Holmes, S. phyloseq: An R package for reproducible interactive analysis and graphics of microbiome census data. *PLoS ONE*. 2013;8. e61217

[15] Nayfach, S, Paez-Espino, D, Strozzi, F, et al. Metegenomic Compendium of 189,680 DNA Viruses From the Human Gut Microbiome. *Nat. Biotechnol.* 2021;39

[16] Hyatt, D, Chen, G-L, LoCascio, PF, et al. Prodigal: prokaryotic gene recognition and translation initiation site identification. *BMC Bioinformatics*. 2010;11. 119

[17] Haft, DH, DiCuccio, M, Badretdin, A, et al. RefSeq: An update on prokaryotic genome annotation and curation. *Nucleic Acids Res.* 2018;46. D851–D860

[18] Coelho, LP, Alves, R, Monteiro, P, et al. NG-meta-profiler: fast processing of metagenomes using NGLess, a domain-specific language. *Microbiome*. 2019;7. 84

[19] Li, D, Liu, C-M, Luo, R, et al. MEGAHIT: an ultra-fast single-node solution for large and complex metagenomics assembly via succinct de Bruijn graph. *Bioinformatics*. 2015;31. 1674–1676

[20] Kang, DD, Li, F, Kirton, E, et al. MetaBAT 2: an adaptive binning algorithm for robust and efficient genome reconstruction from metagenome assemblies. *PeerJ*. 2019;7. e7359

[21] **Almeida, A, Nayfach, S**, Boland, M, et al. A unified catalog of 204,938 reference genomes from the human gut microbiome. *Nat. Biotechnol.*

[22] Chaumeil, P-A, Mussig, AJ, Hugenholtz, P, et al. GTDB-Tk: a toolkit to classify genomes with the Genome Taxonomy Database. *Bioinformatics*. 2019. btz848

[23] Zielezinski, A, Deorowicz, S, and Gudyś, A. PHIST: fast and accurate prediction of prokaryotic hosts from metagenomic viral sequences. *Bioinformatics*. 2022;38. 1447–1449

[24] Coutinho, FH, Zaragoza-Solas, A, López-Pérez, M, et al. RaFAH: Host prediction for viruses of Bacteria and Archaea based on protein content. *Patterns*. 2021;2. 100274

[25] **Gregory, AC, Zablocki, O**, Zayed, AA, et al. The Gut Virome Database Reveals Age-Dependent Patterns of Virome Diversity in the Human Gut. *Cell Host Microbe*. 2020. 1–17

[26] Bland, C, Ramsey, TL, Sabree, F, et al. CRISPR Recognition Tool (CRT): a tool for automatic detection of clustered regularly interspaced palindromic repeats. *BMC Bioinformatics*. 2007;8. 209

[27] Laslett, D, and Canback, B. ARAGORN, a program to detect tRNA genes and tmRNA genes in nucleotide sequences. *Nucleic Acids Res.* 2004;32. 11–16

[28] Hockenberry, AJ, and Wilke, CO. BACPHLIP: predicting bacteriophage lifestyle from conserved protein domains. *PeerJ*. 2021;9. e11396

[29] Ruscheweyh, H-J, Milanese, A, Paoli, L, et al. mOTUs: Profiling Taxonomic Composition, Transcriptional Activity and Strain Populations of Microbial Communities. *Curr. Protoc.* 2021;1. e218

[30] Mende, DR, Letunic, I, Maistrenko, OM, et al. proGenomes2: an improved database for accurate and consistent habitat, taxonomic and functional annotations of prokaryotic genomes. *Nucleic Acids Res.* 2019;48. D621–D625
